# Impact of target site distribution for Type I restriction enzymes on the evolution of methicillin-resistant *Staphylococcus aureus* (MRSA) populations

**DOI:** 10.1093/nar/gkt535

**Published:** 2013-06-14

**Authors:** Gareth A. Roberts, Patrick J. Houston, John H. White, Kai Chen, Augoustinos S. Stephanou, Laurie P. Cooper, David T.F. Dryden, Jodi A. Lindsay

**Affiliations:** ^1^EaStCHEM School of Chemistry, University of Edinburgh, The King’s Buildings, Edinburgh EH9 3JJ, UK and ^2^Division of Clinical Sciences, St. George’s, University of London, Cranmer Terrace, London, SW17 0RE, UK

## Abstract

A limited number of Methicillin-resistant *Staphylococcus aureus* (MRSA) clones are responsible for MRSA infections worldwide, and those of different lineages carry unique Type I restriction-modification (RM) variants. We have identified the specific DNA sequence targets for the dominant MRSA lineages CC1, CC5, CC8 and ST239. We experimentally demonstrate that this RM system is sufficient to block horizontal gene transfer between clinically important MRSA, confirming the bioinformatic evidence that each lineage is evolving independently. Target sites are distributed randomly in *S. aureus* genomes, except in a set of large conjugative plasmids encoding resistance genes that show evidence of spreading between two successful MRSA lineages. This analysis of the identification and distribution of target sites explains evolutionary patterns in a pathogenic bacterium. We show that a lack of specific target sites enables plasmids to evade the Type I RM system thereby contributing to the evolution of increasingly resistant community and hospital MRSA.

## INTRODUCTION

*Staphylococcus aureus* is a common pathogenic bacterium particularly noted for its acquisition of resistance to antibiotics. The worldwide problem of Methicillin-resistant (MRSA) strains is now not only confined to hospitals but is also increasing in the wider community owing to the emergence of new clones such as *S. aureus* USA300 (1–3). *Staphylococcus aureus* is the leading cause of bacterial infections involving the bloodstream, lower respiratory tract and skin and soft tissue in many developed countries, including the USA (4).

Approximately 10 lineages of *S. aureus* dominate in humans and isolates of the same lineage exchange DNA at higher frequency than isolates belonging to different lineages (5). This may explain the observation from bioinformatic studies that the distribution of mobile genetic elements (MGEs) such as plasmids and bacteriophage is lineage dependent (6–8) and suggests each lineage is evolving relatively independently. As these MGEs encode many virulence and resistance genes, the lack of gene exchange between the dominant MRSA lineages has likely delayed the evolution of new clones (1). When MRSA clones acquire new MGEs, they can colonize new niches and host groups. Recently, this has resulted in hospital-associated MRSA, community-associated MRSA and livestock-associated MRSA clones that pose significant new challenges for healthcare and agriculture (1).

Despite its notoriety, *S. aureus* is actually a difficult organism to transform; therefore, the acquisition of MGEs encoding antibiotic resistance is, perhaps fortunately for its hosts, a rather slow process. This low efficiency of horizontal gene transfer is due in part to the presence of DNA restriction and modification (RM) systems on the *S. aureus* genome such as the Type I RM system (given the generic name of Sau1) in which the modification methyltransferase (MTase) sustains the methylation of defined target recognition sequences (TRS) on host DNA and the restriction endonuclease cleaves foreign DNA containing unmethylated copies of the TRS (9).

**Figure 1.**
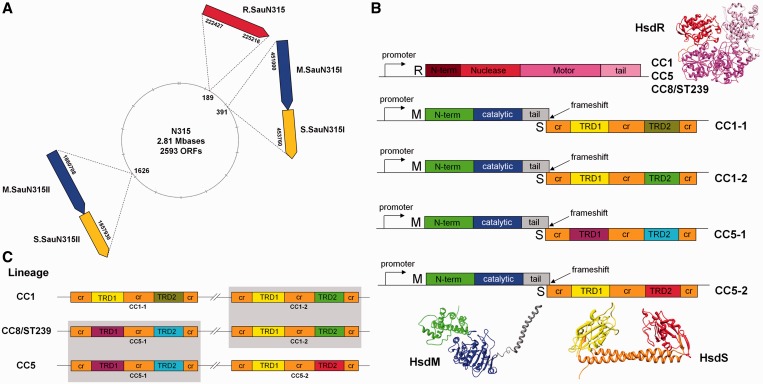
The *hsdR*, *hsdM* and *hsdS* genes of *S. aureus*. (**A**) Genome organization of the RM genes in *S. aureu*s showing the unusual arrangement of the *hsdR* gene that is separate from two copies of the *hsdMS* genes. Genome N315 (CC5) is shown, with restriction genes (*hsdR*) as red arrows, modification genes (*hsdM*) as blue arrows and specificity genes (*hsdS*) as yellow arrows. The numbers inside the circle refer to the ORF number and numbers outside the circle are the genome coordinates. Figures derived from whole-genome sequence and information in REBASE (11,12). (**B**) Domain structure of the proteins encoded by *hsd* genes. Each lineage carries a single *hsdR* gene the product of which carries an N-terminal domain (brown), a nuclease domain (red), a motor domain (cyan) and a tail domain (pink), which can function with the products of either of the two copies of the *hsdS* and *hsdM* genes. The gene organization for expression of the M and S subunits of a typical Type I RM system features a single promoter to drive expression, and there is a frameshift at the junction between the two open reading frames. The M subunit contains an N-terminal domain (green), a catalytic domain (blue) and a C-terminal tail (grey). The S subunit contains conserved regions (cr, orange) around the two TRDs (various colours). Ribbon cartoon models of each subunit of the EcoR124I Type I RM enzyme (13) with domains coloured as above are also shown. The EcoR124I amino acid sequences show homology with those of the *S. aureus* Type I RM enzymes (Supplementary Figures S3 and S4). Each S subunit in this work has a C-terminal EGFP and hexa-His tag added to allow the MTase to be easily purified, but this is not shown here for clarity (14). (**C**) *hsdS* gene variant distribution. Each of the *S. aureus* lineages encode two copies of the *hsdS* gene that are different from each other in the TRDs (various colours). Identical *hsdS* genes conserved between different lineages have matching TRD colours and are shaded. The *hsdS* variants in lineages CC8 (which are the same as for ST239) are homologous to one of those carried in lineages CC1 and CC5, respectively. *hsdR* and *hsdM* genes are highly conserved across the lineages.

Of note was the discovery that the Sau1 systems found in strains of *S. aureus*, correlated perfectly with the lineages (and Clonal Complex or ‘CC’ groups) into which *S. aureus* strains are divided (5,10). The Sau1 systems, encoded by the genes ‘*host specificity for DNA*’ (*hsd*), have conserved Restriction (R) subunits and conserved Modification (M) subunits, but the Sequence specificity (S) subunits vary depending on the CC group (Figure 1A). Each lineage or CC group typically has a single *hsdR* gene distant from two copies of the *hsdM* and *hsdS* genes (Table 1 shows the genes and genome coordinates for the systems studied in this work). The genes for Type I RM systems usually comprise an *hsdR* gene with its own promoter and a separate promoter for the *hsdM* and *hsdS* genes, the open reading frames of which usually overlap by a small number of nucleotides (Figure 1B). This organization is observed for the Sau1 Type I RM systems (5,10,11,15). The R subunits and the M subunits are 99% identical between different CC groups (Supplementary Figures S1 and S2), thus allowing a single R subunit to function with each pair of M and S subunits. They show considerable homology to the R and M subunits of the EcoR124I Type I RM system, although the level of identity is too low to indicate that the Sau1 RM systems are in the same Type IC family as EcoR124I but instead form their own family (Supplementary Figures S3 and S4). Not only are the two pairs of *hsdM-hsdS* genes distant from the *hsdR* gene, they are also distant from each other on the chromosome (Figure 1A and Table 1) and lie in two genomic islands (5,10,11). This collection of five genes allows each lineage to recognize two different TRS (Figure 1B), but why the *hsdR* gene has come to be so distant from the other genes is not clear. *Lactococcus lactis* also shows separation of the Type I RM genes with extra copies of different *hsdS* lying on plasmids complementing the *hsdR*, *hsdM* and *hsdS* genes on the chromosome (16).

**Table 1.** The *S. aureus* strains examined in this work showing their CC identification, the RM system expressed and the TRSs for the Type I RM system (Y = C or T, *N* = any base)

*S. aureus* Clonal Complex	DNA cloned from isolate	Official REBASE names (12) and GenBank Protein ID	Genome coordinates	Proposed names for complete RM enzyme	Type I RM system	TRS (5′–3′)
CC1	MW2	M.SauMWORF392P and S.SauMWORF392P; BAB94257.1 and BAB94258.1	440310-441866 and 441859-443118	SauMW2I	CC1-1	CCAY(N)_5_TTAA
		M.SauMWORF1751P and S.SauMWORF1751P; BAB95616.1 and BAB95615.1	1901740-1903296 and 1900548-1901747	SauMW2II	CC1-2	CCAY(N)_6_TGT
		R.SauMWORF169P	200816-203605			
CC5	N315	M.SauN315ORF391P and S.SauN315ORF391P; BAB41620.1 and BAB41621.1	451000-452556 and 452549-453760	SauN315I	CC5-1	ATC(N)_5_CCT
		M.SauN315ORF1626P and S.SauN315ORF1626P; BAB42894.1 and BAB42893.1	1859152-1860708 and 1857930-1859159	SauN315II	CC5-2	CCAY(N)_6_GTA
		R. SauN315ORF189P and BAB41217.1	222427-225216			
CC8/ST239					CC1-2	CCAY(N)_6_TGT
					CC5-1	ATC(N)_5_CCT

S subunits are of mosaic structure with two target recognition domains (TRDs) flanked by highly conserved amino acid sequences (Figure 1B and Supplementary Figure S5) (9). The DNA TRS recognized by an S subunit typically consists of 3 or 4 defined base pairs followed by a non-specific spacer of ∼6 bp followed by a second set of 3–5 defined base pairs. (9) The first TRD recognizes the first part of the TRS, the second TRD recognizes the second part of the TRS and the conserved amino acid sequence separating the TRDs defines the length of the non-specific spacer in the TRS (9). If TRDs recognize the same DNA sequence, then they show a high degree of amino acid identity, but the level of identity is low if they recognize different sequences (Supplementary Figure S5). The TRD amino acid sequences all show a bias towards basic residues resulting in estimated values for the isoelectric point for the S subunits of between 9.2 and 9.5, as one would anticipate for a DNA-binding protein (17). The mosaic structure most probably derives from an ancestral ‘half-S’ gene, which underwent multiple duplication and recombination events to produce the range of mosaics observed today. Functional half-S subunits can be generated experimentally and recognize symmetrical TRS (18,19). This mosaic structure is not only obvious in *S. aureus* (5,11,15) but also in extensive genome analyses of *Mycoplasma pulmonis* (20), *Neisseria meningitidis* (21), *Helicobacter pylori* (22) and *Bacteroides fragilis* (23). Inversions are also evident allowing phase variation and the generation of multiple TRS within a single bacterial population (20,23).

*Staphylococcus aureus* genomes usually contain two copies of *hsdS* that each have a different sequence. Figure 1C shows the distribution of the *hsdS* gene variants, as determined from mosaic structure of their TRD sequences, in whole-genome sequences of MRSA from the major lineages CC1, CC8/ST239 and CC5. Lineage CC8 *hsdS* genes show strong homology with the CC5-1 and CC1-2 genes that are found in CC5 and CC1 isolates, respectively. ST239 isolates have evolved from CC8 isolates and have maintained the same *hsdS* genes (5,10). The predicted protein sequence of the CC5-1 S subunit is 100% conserved over 403 aa between CC5 and CC8 isolates, including representative isolates from CC5 (*S. aureus* N315 and Mu50), CC8 (*S. aureus* 8325, USA300 FPR3757, Newman and COL) and ST239 (*S. aureus* TW20). The CC1-2 S subunit is 99% conserved over the entire 399aa between CC1 and CC8 isolates, including representative isolates from CC1 (*S. aureus* MW2, MSSA476), CC8 (*S. aureus* 8325, USA300 FPR3757, Newman and COL) and ST398 (*S. aureus* TW20). The two amino acid changes are located outside of the TRDs.

The TRS for Type I RM systems are extremely difficult to determine because their restriction endonucleases do not cut DNA at their TRS but at random sequences distant from the TRS. Their determination has relied on the comparison of transformation efficiency or restriction of a library of DNA fragments followed by computer analysis. Methylation occurs at defined positions in the target site, but finding the location is difficult, although single-molecule real-time sequencing methods for the analysis of whole-genome modification patterns are being developed (24).

In this article, we have identified the specific DNA sequence targets for the dominant MRSA lineages CC1, CC5, CC8 and ST239. Identification of the TRS for the Sau1 Type I RM system and their distribution in *S. aureus* genomes allows the understanding and prediction of how they contribute to the evolution of *S. aureus* populations. In particular, the spread of MGEs encoding virulence and resistance genes is fundamental to the development of newly evolving MRSA clones and will be dependent on the TRS they carry. Furthermore, identification of TRS enables strategic design of genetic vectors that can overcome Type I RM systems and will enable genetic manipulation of clinically relevant pathogenic strains in the research laboratory.

## MATERIALS AND METHODS

### Preparation of enzymes

The *hsd* genes for the Sau1 MTases (Table 1) were amplified from genomic DNA and used to replace the genes for the EcoKI MTase in the expression plasmid pJFMSEGFP (14). All four Sau1 MTase operons lacked BamHI restriction sites in their open reading frames, thus allowing a common cloning strategy using only one vector. Following the success of producing EcoKI MTase with the S subunit tagged with the enhanced green fluorescent protein (EGFP) and a hexa-Histidine tag from plasmid pJFMSEGFP (14), we engineered an expression construct for all four MTases. Through the use of the polymerase chain reaction (PCR), using oligonucleotides pJFMSEGFPhisBS (5′GAGTGAATCCCCGGGGATCCGTCGACC 3′) and pJFMSEGFPhisTS (5′AGTCAGTCAGGGATCCATGGTGAGCAAGGGCGAGGAGCTG3′) with pJFMSEGFP as template, we obtained a linear PCR derivative of the vector. The resulting PCR product allows a coding sequence to be introduced downstream of the tac promoter and fused in frame with EGFP-His-tag following digestion of the ends of the coding sequence with BamHI. The *hsdM* -*hsdS* operon was amplified from the appropriate *S. aureus* genomic DNA by PCR, using a universal Sau1M oligonucleotide, (5′AGTCAGTCAGGGATCCAAGAAGGAGATATACATATGTCTATTACTGAAAAACAACG3′) in every reaction, in combination with a locus-specific oligonucleotide, homologous to the end of the appropriate *hsdS* as follows:

CC5-1 BS (5′GATCGAATTCCGGATCCTAAGAACATTTTTTGTAAAAAGG3′),
CC5-2 BS (5′GATCGAATTCCGGATCCAACAAACATTTTTTGTAATAGTTC3′),
CC1-1 BS (5′GATCGAATTCCGGATCCAATAAACATTTTCTGTAAAAACGCC3′),

CC1-2 BS (5′GATCGAATTCCGGATCCAATAAACATTTTTTGTAATAGTTC3′). The resulting PCR products were purified, cut with BamHI and ligated into the BamHI interval of the vector PCR product. The universal Sau1M oligonucleotide assumes that the M subunit commences with the sequence MSITEKQRQQQ and ignores unconserved sequences upstream of the conserved ATG start codon for methionine. The plasmids were named pCCX-Y where X is the number of the clonal complex and Y of the loci. The *hsdR* gene (Genbank BAB41410.1) was amplified from isolate N315 (CC5) by PCR using oligonucleotides as follows:

Sau1 hsdRFOR (5′AAGGAGATATACCATGGCATACCAAAGTGAATACGC3′) and

Sau1 hsdRREV (5′GAATTCGGATCCTTACACACCGTATTTTTCAGTTG3′). The fragment was cut with NcoI and BamHI and ligated into the NcoI - BamHI interval of pRSFDuet-1. The DNA sequence of the chosen clone agreed perfectly with the desired sequence. The plasmid was named phsdR. In each case, the DNA sequences of individual clones were confirmed.

**Figure 2.**
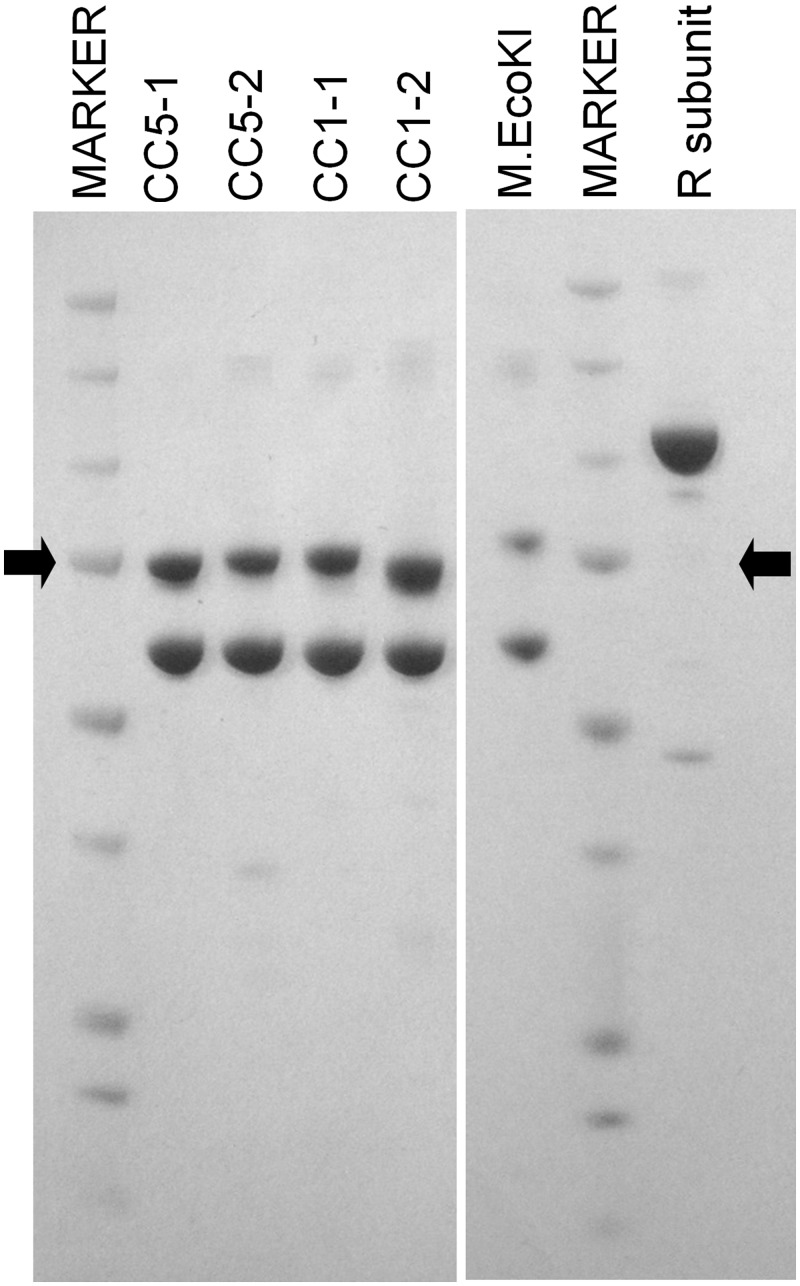
SDS–PAGE analysis of purified proteins. The upper band in the MTase preparations is the EGFP-His-tagged S subunit and the lower band is the M subunit. The EcoKI MTase with the EGFP-His-tagged S subunit is shown for comparison. The purified R subunit is also shown. The markers have molecular masses of 250, 150, 100, 75, 50, 37, 25 and 20 kDa (Biorad precision plus protein standards). The arrows indicate the 75 kDa size.

Protein expression was induced by adding isopropyl β-D-1-thiogalactopyranoside to 1 mM to transformed *Escherichia coli* BL21(DE3) cultures growing at 37°C. Cells containing either the pCCX-Y plasmids expressing the MTase or cells containing the phsdR were grown. The MTases and the R subunit were purified separately. Induction was for 3–4 h at 25°C or 30°C. The cells were disrupted by intermittent sonication for ∼20 min with cooling on ice using a Soniprep 150 sonicator (Sanyo, Tokyo, Japan) fitted with a 9 mm diameter probe and then centrifuged at 20 000*g* for 90 min at a temperature of 4°C. Proteins were purified by HisTrap chromatography, size exclusion chromatography, diethylaminoethyl (DEAE) anion exchange chromatography and, if necessary, Heparin HiTrap chromatography (GE Healthcare, Uppsala, Sweden) and were >98% pure as judged by Coomassie Blue staining of SDS–polyacrylamide gels. The purified proteins were stored at −20°C after addition of glycerol to 50% (v/v). NaCl was also added to 0.2M for the CC1-1, CC1-2 and CC5-1 MTase preparations and to 0.5 M for the CC5-2 MTase and R subunit preparations to maintain protein solubility. Extinction coefficients were calculated at 280 nm (25) and assuming an M**_2_**S**_1_** stoichiometry for the MTases (and including the EGFP and hexa-His tag on the S subunit) and a monomeric R subunit (13,26). The purified proteins were analysed by SDS–PAGE and estimated to be >95% pure (Figure 2). To form an active restriction enzyme, the MTase preparations were mixed with the R subunit.

### Target recognition sites

Endonuclease cleavage assays were performed by incubating a library of plasmids, based on insertion of known DNA sequences ligated into the EcoRI-BamHI interval of pUC19, with MTase and R subunit for 15 min at 37°C and analysed using agarose gel electrophoresis. Cleavage sites are distant from the target site for these enzymes; therefore, a computer program, RMsearch, was used to search for target sequences present in plasmids cut by the enzyme and not present in uncut plasmids (27,28). The main set of plasmids were based on the DNA sequence of phage PhiED1 (a kind gift from Dr Garry Blakely, Edinburgh) as described in Supplementary Materials and Methods. Also described in the Supplementary Materials and Methods are sets of plasmids containing smaller fragments of phage PhiED1 and inserts from phage lambda (a kind gift from Iain Murray of New England Biolabs). Typically, ∼40 plasmids were analysed for each enzyme. Cleavage sites were then confirmed by inserting a short defined oligonucleotide sequence containing the putative target into pUC19 (sequences described in Supplementary Materials and Methods). Reaction digests had a total volume of 50 µl and a typical digest was prepared using 5 µl of 10× NEBuffer 4 [New England Biolabs; 50 mM potassium acetate, 20 mM Tris–acetate, 10 mM magnesium acetate, 1 mM dithiothreitol (pH 7.9)], 2 mM ATP, 0.64 µM S-adenosyl-L-methionine, 0.01 mg of bovine serum albumin and 10 µl of the enzyme stock. The enzyme stock was prepared in a volume of 50 µl with 5 µl of 10xNEBuffer 4 with final concentrations of 1.16 µM R subunit and 0.42 µM MTase, thus ensuring an excess of R over the MTase to give formation of the R**_2_**M**_2_**S**_1_** RM enzyme.

### Staphylococcus aureus strains and plasmids

JE2 is a CC8 MRSA and belongs to the USA300 clonal group. It is plasmid negative, has been derived from strain LAC and has high genome similarity to FPR3757 (29). JE2 mutants in *hsdS* (NE1258, NE982), *hsdR* (NE667) and Type IV restriction endonuclease (NE513) were generated by mariner transposon mutagenesis. All JE2-derived isolates were obtained from NARSA and are the original isolates constructed at the University of Nebraska (31). N315 is a CC5 MRSA (11). The shuttle vector pCN36 (31) was used in all transfer experiments. It carries a *tetM* selectable marker and has two CC5-1 TRS, one CC1-2 TRS and no CC5-2 TRS.

### Electroporation

Plasmid DNA was prepared from *S. aureus* using Wizard® Plus SV Minipreps DNA Purification (Promega, UK) with an additional lysostaphin (L4402, Sigma-Aldrich, UK) treatment step and concentration measured by UV spectrometry. Electroporation was carried out essentially as described previously (32) and transformants selected on agar supplemented with tetracycline at 5 µg/ml.

### Bioinformatics

TRS distribution was analysed using NCBI Sequence Viewer 2.21 (http://www.ncbi.nlm.nih.gov/projects/sviewer/). Annotated *S. aureus* whole-genomes (*n* = 18) (7), and the MGEs bacteriophage (*n* = 50) (8), Staphylococcal Cassette Chromosomes carrying the *mecA* gene (*n* = 35) (33) and plasmids (*n* = 233) (6) were analysed; these genomes and MGEs are listed in Supplementary Table S1. We manually checked the whole genome of the representative MRSA252 isolate and found no evidence that target sites were dismissed owing to target overlap using this method. Values were expressed as the average TRS per kb of each type of genome analysed. Statistical comparison of TRS frequency using the Mann–Witney two-tailed test was calculated by dividing observed TRS per genome by expected numbers of TRS per genome (based on whole genome TRS frequency). Comparing the numbers of genomes with zero TRS used the chi-square test. TRS per plasmid was visualized using Excel. Protein extinction coefficients and isoelectric points were calculated using http://www.scripps.edu/∼cdputnam/protcalc.html.

## RESULTS

### Protein preparation

The purified proteins were analysed by SDS–PAGE and estimated to be >95% pure (Figure 2). To form an active restriction enzyme, the MTase preparations were mixed with an excess of the R subunit.

### Naming of the Sau1 systems

As the putative open reading frames for the Sau1 systems from CC1 and CC5 have been overexpressed and shown to be active, they can be assigned formal names according to the convention (34). These names are given in Table 1 as SauMW2I, SauMW2II, SauN315I and SauN315II. However, as these names are specific to the particular *S. aureus* strain rather than to the lineage containing the strain, they are not useful when trying to determine the TRS for Type I RM systems shared with other lineages. The naming difficulty is compounded when a single R subunit from a single strain can be used to complement the MTases from any other strain. Thus, although the formal names should be used for descriptions of the individual enzymes, it is easier to use names based on the CC groups when comparing groups of these *S. aureus* enzymes. Table 1 also gives our suggested names based on CC groups, which we will use in the remainder of this article, these being CC1-1, CC1-2, CC5-1 and CC5-2 for SauMW2I, SauMW2II, SauN315I and SauN315II, respectively. Using these lineage-based names, the Type I enzymes are referred to as the CCX-Y MTase for the M_2_S_1_ complex and the CCX-Y RM enzyme for the mixture of the M_2_S_1_ MTase with an excess of the R subunit to form the R_2_M_2_S_1_ complex. The X refers to the clonal complex, and the Y refers to the proximity of the *hsdM-hsdS* genes to the start of the genome sequence, with 1 indicating genes closer to the first nucleotide in the genome sequence than those labelled 2. This nomenclature also allows the TRS to be referred to as CCX-Y sequences or sites.

### Endonuclease activity and target recognition site determination

**Figure 3.**
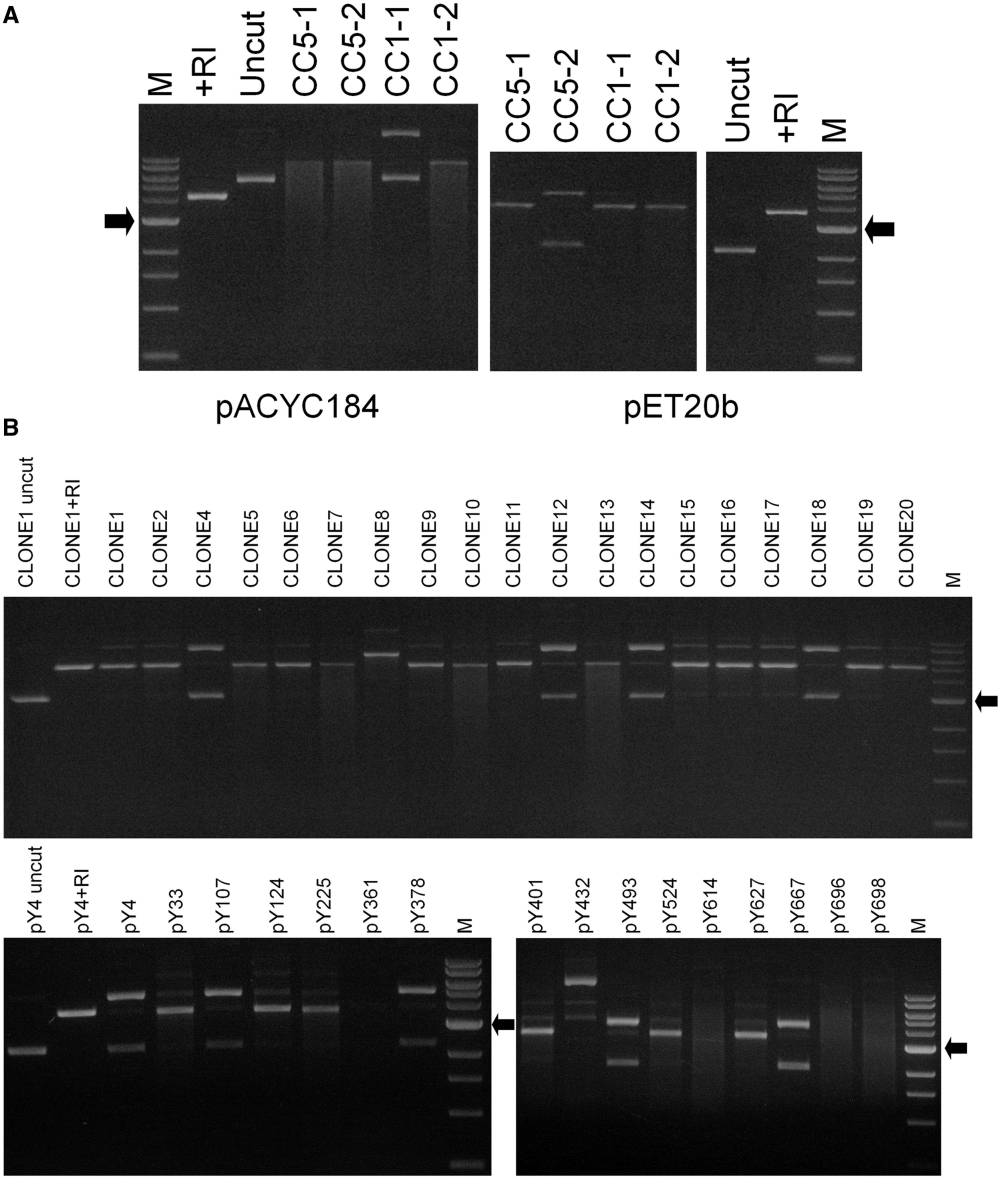
(**A**) Agarose gel analysis of DNA cleavage activity of the prepared Sau1 restriction enzymes using plasmids pACYC184 and pET20b. CC1-1, CC1-2, CC5-1 and CC5-2 indicate the Type I restriction enzyme used. pACYC184 has no site for CC1-1 but multiple sites for the other Type I enzymes as indicated by the smearing of the cleaved DNA. CC1-1 nicks the plasmid, but this is non-specific. pET20b has no site for CC5-2 and a single site for the other Type I enzymes as indicated by the linearization of the plasmid. CC5-2 nicks the plasmid, but this is non-specific. (**B**) Example cleavage assay using the CC5-1 enzyme against the 2 kb CLONE1-20 library and the pY library described in Supplementary Material. CLONE8, pY361, pY432, pY614, pY696 and pY698 either showed unexpected molecular masses or too many sites resulting in an uninterpretable smear and were not included in our analyses. M = 1 kb markers with the arrow indicating the 3 kb size (New England Biolabs), uncut indicates the supercoiled plasmid, +RI indicates the plasmid linearized by EcoRI.

*Escherichia coli* was transformed with plasmids expressing the *hsdM* and *hsdS* gene variants and the MTase complex purified and combined with purified R subunit. Figure 3 shows the ability of the RM enzymes to cleave a selection of plasmids from our library of plasmids and Supplementary Table S2 summarizes the ability of the Sau1 RM enzymes to cleave the full library of plasmids. The plasmid preparations usually showed only supercoiled closed circular DNA but when incubated with the Sau1 RM enzymes, varying amounts of nicked open circular DNA were produced even in the absence of ATP. We attribute this nicking activity to the presence of a small amount of a contaminating nuclease in our enzyme preparations. The key indicator of cleavage by the Sau1 RM enzymes is the ATP-dependent production of linearized DNA if the plasmid contains a single TRS or a smear of products of different lengths if the plasmid contains multiple TRS. The smearing occurs because Type I RM enzymes cleave at random distances from their TRS rather than at the TRS.

The pUC19 plasmid was not cut by the CC1-1, CC5-1 and CC5-2 RM enzymes; thus, any cleavage of pUC19 containing an inserted fragment of DNA indicated the presence of a TRS in the insert. A computer comparison of the sequences of cleavable and uncleavable plasmids allowed determination of the candidate TRS for these enzymes. To aid this process, subsets of pUC19 with shorter and shorter DNA inserts had to be constructed and lastly confirmation of the proposed TRS was obtained using a short synthetic oligonucleotide sequence inserted into pUC19.

The CC1-2 RM enzyme cleaved pUC19 DNA to a linear form; thus, it has a single TRS for CC1-2. To use the plasmid library based on pUC19, the DNA was first cut with either BamHI or EcoRI to a linear form. Subsequent incubation with CC1-2 RM enzyme would leave the DNA in a linear form if no additional TRS were present in the DNA, but a smear would result if the insert had a TRS. This smearing is the result expected if the linear DNA contains two or more copies of the TRS. Computer analysis and the pUC19 plasmid subsets allowed identification of the TRS for the CC1-2 RM enzyme.

Table 1 shows the TRS determined for lineages CC1 and CC5. These are typical of target sequences for Type I RM systems, although that for CC5-1 RM enzyme is one of the shortest yet found. Our data do not define which adenine nucleotides are the target for methylation by the enzymes, but apart from the sequences TTAA and TGT, only a single location in each part of the TRS is possible (i.e CCAY, ATC, CCT, GTA will be methylated at the underlined positions either on the A shown or on the A on the complementary strand).

### Distribution of target recognition sites in genomes and plasmids

**Figure 4.**
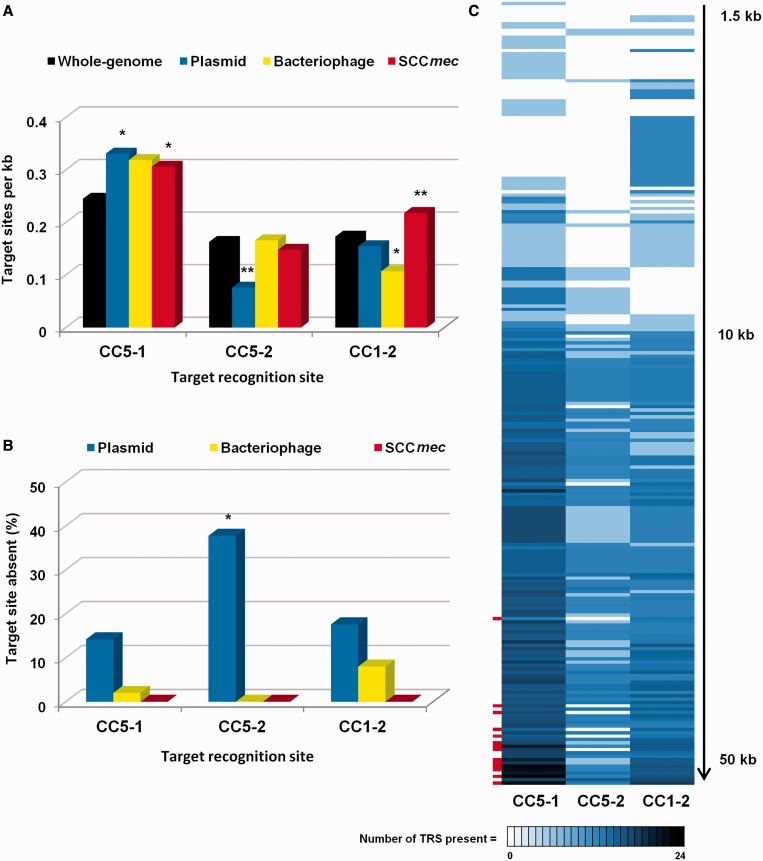
Plasmids have fewer CC5-2 target sites than expected. (**A**) Average target recognition sites (TRS) per kb for CC5-1, CC5-2 and CC1-2 enzymes in *S. aureus* sequences of whole-genomes (*n* = 18), plasmid (*n* = 233), bacteriophage (*n* = 50) and Staphylococcal cassette chromosomes with *mecA* (*n* = 35). By Mann–Witney two-tailed test, enzyme 5-1 had significantly more TRS in plasmids, and SCC*mec* than genomes (*P* < 0.01); enzyme 5-2 had significantly less TRS in plasmids than genomes (*P* < 0.0001); enzyme 1-2 had significantly less TRS in phage (*P* < 0.01) but significantly more TRS in SCC*mec* (*P* < 0.0001). Asterisk indicates significant, *P* < 0.01, Double asterisk indicates significant, *P* < 0.0001. (**B**) Percentage of MGEs lacking target sites for CC5-1, CC5-2 and CC1-2 in sequences of plasmid (*n* = 233), bacteriophage (*n* = 50) and Staphylococcal cassette chromosomes with *mecA* (*n* = 35). There are significantly more plasmids missing TRS for 5-2 than missing TRS for 5-1 or 1-2 (Chi square, *P* < 0.0001, indicated by asterisk) (**C**) TRS distribution profile of plasmid sequences (*n = *233) ordered by size shows small plasmids (<10 kb) are more likely to be missing a CC5-2 TRS than missing a CC5-1 or CC1-2 TRS (Chi square, *P* < 0.0001), and that large conjugative plasmids (*tra*+; indicated by red dash, *n* = 14) are more likely to have zero CC5-2 TRS than zero CC5-1 or CC1-2 TRS (Chi square, *P* < 0.001). Each horizontal line represents a plasmid and is shaded according to the number of TRS.

The distribution of each identified TRS in sequenced *S. aureus* genomes (Figure 4A) revealed a random distribution of each amongst whole genomes of *S. aureus* from various lineages, as well as several MGEs. The exceptions were plasmids, which specifically harboured fewer sites for the CC5-2 RM enzyme and often lacked these sites altogether (Figure 4B). A direct comparison of 233 plasmids showed that this was not confined to small plasmids, which might be expected to carry fewer TRS by chance owing to their small size (Figure 4C). Notably, multiple large conjugative plasmids, identified by the carriage of the *tra* gene locus for transfer (red), were particularly deficient in the TRS for the CC5-2 RM enzyme (Figure 4C). We hypothesized that this represents evolution of the larger plasmids to escape this enzyme and tested this experimentally.

### Transfer of plasmids between lineages

**Figure 5.**
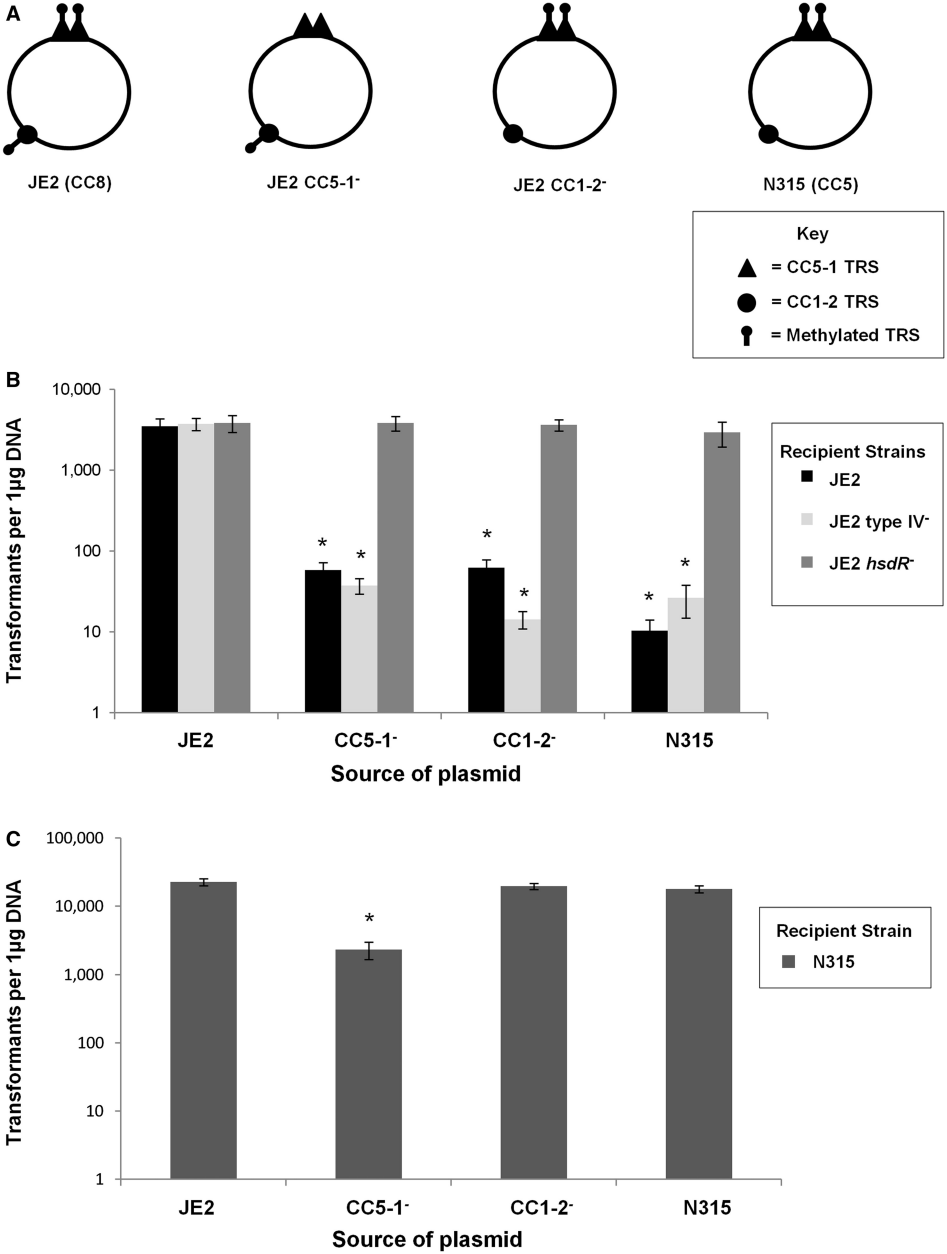
Electroporation of plasmid pCN36 is dependent on Sau1 Modification and Restriction. (**A**) Plasmid methylation profiles of pCN36 when grown in different donor backgrounds. There are two TRS for the CC5-1 enzyme (recognized by both CC8 and CC5 isolates), one target site for CC1-2 (recognized by CC8), and no target sites for CC5-2 (recognized by CC5). CC5-1^− ^and CC1-2^− ^refer to *S. aureus* JE2 (CC8) isolates with mutations in *sau1hsdSCC5-1* and *sau1hsdSCC1-2*, respectively. (**B**) Transformation efficiency of pCN36 (tetracycline resistant colonies per 1 μg DNA) into *S. aureus* JE2 (CC8) is dependent on modification with both CC5-1 and CC1-2 and restriction by *sau1hsdR* (5,6), but not with restriction by the Type IV restriction system (35,36). pCN36 prepared from *S. aureus* N315 (CC5) is not readily accepted by *S. aureus* JE2 (CC8). (**C**) Transformation efficiency of pCN36 from *S. aureus* JE2 (CC8) to *S. aureus* N315 (CC5) is dependent on CC5-1 modification, and not CC5-2. *S. aureus* N315 (CC5) accepts plasmid at high rates from *S. aureus* JE2 (CC8), as pCN36 does not contain a TRS for CC5-2. Data presented represent average transformation efficiency of three experiments ± SD. Asterisk denotes significant difference *P* < 0.001.

Electroporation of plasmids into *S. aureus* JE2, a CC8 SCC*mec*IV USA300 isolate typical of MRSA circulating in the community in the USA, was controlled by the Type I RM system (Figure 5). Plasmids grown in *S. aureus* JE2 donors deficient in each of the two *hsdS* genes recognizing the CC5-1 and CC1-2 TRS were not modified, and when transferred to parental JE2 recipients, these plasmids were recognised as foreign and restricted (Figure 5A and B). Restriction was due to the Type I RM system and *hsdR* dependent, as when this gene was deleted the unmodified plasmid was transferred at high frequency. Transfer was not restored by deleting the Type IV restriction system, showing no role for this system in transfer of plasmids between the MRSA isolates (although it does prevent transformation of cytosine-methylated plasmids prepared from *E.coli* containing the dcm MTase) (35,36). Similarly, plasmids grown in *S. aureus* N315, a clinical MRSA from lineage CC5, were recognized as foreign and digested by the Type I and not the Type IV restriction system (Figure 5B).

A lack of sites for the CC5-2 RM enzyme in plasmids is crucial for their successful horizontal transfer from CC8 to CC5, as the second TRS recognized in CC5, namely, CC5-1, is shared by the lineages and therefore does not act as a barrier (Figure 5). This was confirmed by electroporation of a plasmid carrying TRS for CC1-2 and CC5-1, but lacking the CC5-2 target sequence (Figure 5). As predicted, the efficiency of transfer from CC5 (N315) to CC8 (JE2 and JE2 Type IV^-^) was low (mean 10.3 transformants per µg of plasmid DNA) (Figure 5B), whereas the efficiency of transfer from CC8 to CC5 was high (mean 22892 transformants per µg of plasmid DNA) (Figure 5C).

This can be explained by the distribution of TRS sites. If the plasmid was modified in the CC8 (JE2) background by only the CC1-2 system (JE2 CC5-1^-^ donor), then the CC5-1 TRS were unmodified, and when transferred to the CC5 (N315), the plasmid was recognized as foreign and restricted (Figure 5C). In contrast, if the plasmid was modified in the JE2 background by only the CC5-1 system (JE2 CC1-2^-^ donor), the N315 recipient would recognize the CC5-1 modified TRS as self, and as there are no unmodified targets for the CC5-2 to recognize, the plasmid transfers successfully (Figure 5C). The results suggest that when plasmids are missing the CC5-2 target sites and originate in a CC8 background, CC5 recipients will not recognize them as foreign, as there is no unmodified TRS remaining to be recognized. Therefore, large naturally occurring resistance plasmids can transfer easily from CC8 to CC5, but not the reverse.

### Implications of target recognition site identification on MRSA evolution

This study investigated the Type I RM target sites of three major *S. aureus* lineages, which are also the parental lineages of four of the most successful and prevalent MRSA lineages worldwide (1,2). The CC8 lineage includes the MRSA clone *S. aureus* USA300 responsible for the majority of community-associated (CA-) MRSA in the USA (3,14), and *S. aureus* USA500, which is a common hospital-associated (HA-) MRSA in the USA and Europe (37). MRSA ST239 clones are the most common HA-MRSA in Asia and South America and found worldwide (38). ST239 arose from a recombination of the CC8 and CC30 lineages, with the resultant clone carrying the CC8 *hsdS* genes (39). CC5 clones such as *S. aureus* USA100 are the most common HA-MRSA in the USA and some regions of Europe and Asia (37). The CC1 clone *S. aureus* USA400 was the original CA-MRSA in the USA and is still widespread (37).

Examples of large plasmids that do not have CC5-2 target sites include the 37 kb conjugative plasmid pUSA03 isolated from *S. aureus* USA300 and encoding resistance to erythromycin and mupirocin (29). This plasmid has eleven targets sites for CC5-1, five for CC1-2 and none for CC5-2. SAP082A, also from *S. aureus* USA300, is a 44 kb conjugative plasmid encoding gentamicin resistance and has 13 target sites for CC5-1, eight for CC1-2 and none for CC5-2 (40). Similar plasmids are reported to be carried in CC5 isolates in the USA (39), suggesting there has been horizontal transfer of this plasmid group between clinical MRSA lineages. This is in contrast to the majority of plasmids that have a distribution correlating with lineage (6). The data suggest that CC5 isolates in hospitals in the USA and the new CC8 isolates from the community in the USA, which are now spreading to hospitals, are exchanging multi-drug resistance plasmids at higher frequency than other lineages.

## DISCUSSION

Few Type I RM target recognition sites have been identified. Here, we report three Type I RM TRS, which together account for those found in four of the most clinically important *S. aureus* and MRSA lineages. Construction of genetically manipulated strains of clinical isolates (41,42) belonging to these lineages will now be possible using vectors constructed without these sites.

The genome location of the Sau1 RM enzymes is also unusual and bears further investigation. The two *hsdM-hsdS* gene pairs are located on genomic islands distant from each other and distant from the single copy of the *hsdR* gene (5,11,15). This arrangement is completely different from arrangement of the *hsd* genes in the immigration control region of the archetypal *E. coli* K12 strain. Large-scale genome rearrangements have presumably occurred in *S. aureus*. The fact that multiple Type I HsdM and HsdS proteins functionally interact with Type I HsdR proteins derived from genes in a separate region of the genome has implications for interpreting bacterial whole-genome sequences. Multiple *hsd* genes in single cells, particularly those that have additional Type I systems encoded on MGEs, may lead to complex and highly variable DNA modification patterns.

As more and more TRDs of S subunits become associated with known recognition sequences, then predicting the TRS in new strains will become a simple matter of comparing new TRD amino acid sequences with ones that have known recognition sequences. Structural modelling of TRDs and their interface with DNA may become possible and a recognition code determined for Type I RM enzymes in a manner similar to that used for the Type II restriction enzyme MmeI and its relatives (43). Such modelling could use the three known crystal structures for S subunits (44,45) and the models of Type I RM enzymes bound to DNA (13,47,48), though the absence of a crystal structure of a DNA-S subunit complex might limit the accuracy of such models. Such modelling would also facilitate the prediction of the adenine methylation sites within the TRS, something that requires considerable experimental effort at the moment (24).

RM systems protect host bacteria from foreign DNA such as bacteriophage. Evidence is accumulating that *S. aureus* populations exchange MGE at high frequency, but this is restricted to isolates from related clones and lineages (49,50), and here, we show that in clinically important MRSA isolates, it is controlled by the lineage-associated Sau1 Type I RM system. Our data suggest that large conjugative plasmids carrying antibiotic resistance genes have evolved to reduce the number of Type I RM target recognition sites to enable them to exchange across lineage barriers. The barrier they evade is specifically from lineages CC8 and ST239 to lineage CC5. CC5 MRSA is the most prevalent type of hospital MRSA in the USA. CC8 isolates from the successful community *S. aureus* USA300 clone were not originally reported to be multi-drug resistant but are increasingly found in hospitals in the USA and increasingly drug resistant (37,40). Our results strongly indicate that this mechanism of restriction evasion may account for the recent reports of multi-drug resistant plasmid exchange reported between CC8 and CC5 isolates in the USA (40). The observation of lower number of restriction enzyme target sites on MGEs than expected by chance has been known for many years (51–57), but rarely has this been so clearly linked to a clinical observation as shown in this work.

The ability to exchange DNA contributes to the success of MRSA clones in the hospital setting by spreading resistance genes as well as enabling rapid adaptation to environmental onslaughts (49,50). The avoidance of TRSs on phage DNA is a well-known mechanism for evading a host RM system (51–54). This avoidance, particularly of palindromes, also occurs on the host chromosome (53–55). The avoidance of the asymmetric TRS typical of Type I RM systems is also apparent (56,57), but no computational study to complement the analysis of palindromic TRS has been performed to our knowledge nor has an analysis of avoidance of Type I TRS on plasmids been published. Thus, our data show that plasmids, in addition to phage, can evolve to lose RM target sites for Type I RM systems to spread antibiotic resistance across restriction boundaries in pathogenic bacteria. Whole-genome sequencing of large plasmids from clinically important MRSA is warranted to track and contain the spread of multi-drug resistant plasmids amongst high-risk MRSA populations.

Lastly, our results indicate that determining the target recognition sites for the many Type I RM systems present in other pathogens (12) such as *H. pylori* (22), *N. meningitidis* (21) and *B. fragilis* (23) would be valuable for understanding the spread of multi-drug resistant plasmids in other organisms. This could be performed experimentally, but a computational search for the avoidance of the asymmetric target sites typical of Type I RM systems could also be envisaged.

## SUPPLEMENTARY DATA

Supplementary Data are available at NAR Online: Supplementary Tables 1 and 2, Supplementary Figures 1–5 and Supplementary Materials and Methods.

Supplementary Data
