# Supplementary material for: Impact of target site distribution for Type I restriction enzymes on the evolution of methicillin-resistant Staphylococcus aureus (MRSA) populations
Source: Nucleic Acids Res. 2013 Jun 14;41(15):7472–84. doi: 10.1093/nar/gkt535 (PMC3753647; doi:10.1093/nar/gkt535)
Supplement: Supplementary Data [file supp_41_15_7472__index.html]

Impact of target site distribution for Type I restriction enzymes on the evolution of methicillin-resistant Staphylococcus aureus (MRSA) populations — Impact of target site distribution for Type I restriction enzymes on the evolution of methicillin-resistant Staphylococcus aureus (MRSA) populations — Supplementary Data 

# Impact of target site distribution for Type I restriction enzymes on the evolution of methicillin-resistant *Staphylococcus aureus* (MRSA) populations

## 

files

**Files in this Data Supplement:**

- Supplementary Data - doc file
