# Supplementary material for: Impact of target site distribution for Type I restriction enzymes on the evolution of methicillin-resistant Staphylococcus aureus (MRSA) populations
Source: Nucleic Acids Res. 2013 Jun 14;41(15):7472–84. doi: 10.1093/nar/gkt535 (PMC3753647; doi:10.1093/nar/gkt535)
Supplement: Supplementary Data [file supp_gkt535_nar-00155-h-2013-File007.doc]

**Impact of Target Site Distribution for Type I Restriction Enzymes on the Evolution of Methicillin Resistant *Staphylococcus aureus* (MRSA) Populations.**

Gareth A. Roberts, Patrick J. Houston, John H. White, Kai Chen, Augoustinos S. Stephanou, Laurie P. Cooper, David T.F. Dryden, Jodi A. Lindsay.

**Supplementary information.** Supplementary Figures S1, S2, S3 and S4, supplementary Tables T1 and T2 and supplementary materials and methods.

Supplementary Figure S1. Alignment of R subunit amino acid sequences from lineages CC1 and CC5. R.SauMWORF169P and R. SauN315ORF189P

CLUSTAL 2.1 multiple sequence alignment

SauN315ORF189P MAYQSEYALENEMMNQLEQLGYERVTIRDNKQLLDNFRTILNERHADKLEGNPLTDKEFQ 60

SauMWORF169P MAYQSEYALENEMMNQLEQLGYERVTIRDNKQLLDNFRTILNERHADKLEGNPLTDKEFQ 60

************************************************************

SauN315ORF189P RLLTMIDGKSIFESARILRDKLPLRRDDESEIYLSFLDKKSWCKNKFQVTNQVSVEDTYK 120

SauMWORF169P RLLTMIDGKSIFESARILRDKLPLRRDDESEVYLSFLDTKSWCKNKFQVTNQVSVEDTYK 120

*******************************:******.*********************

SauN315ORF189P ARYDVTILINGLPLVQVELKRRGIDINEAFNQVKRYRKQNYTGLFRYIQMFIISNGVETR 180

SauMWORF169P ARYDVTILINGLPLVQVELKRRGIDINEAFNQVKRYRKQNYTGLFRYIQMFIISNGVETR 180

************************************************************

SauN315ORF189P YFSNNDSELLKSHMFYWSDKQNNRINTLQSFAESFMRPCQLAKMISRYMIINETDRILMA 240

SauMWORF169P YFSNNDSELLKSHMFYWSDKQNNRINTLQSFAESFMRPCQLAKMISRYMIINETDRILMA 240

************************************************************

SauN315ORF189P MRPYQVYAVEALIQQATETGNNGYVWHTTGSGKTLTSFKASQILSQQDDIKKVIFLVDRK 300

SauMWORF169P MRPYQVYAVEALIQQATETGNNGYVWHTTGSGKTLTSFKASQILSQQDDIKKVIFLVDRK 300

************************************************************

SauN315ORF189P DLDSQTEEEFNKFAKGAVDKTFNTSQLVRQLNDKSLPLIVTTIQKMAKAIQGNAHLLEQY 360

SauMWORF169P DLDSQTEEEFNKFAKGAVDKTFNTSQLVRQLNDKSLPLIVTTIQKMAKAIQGNAHLLEQY 360

************************************************************

SauN315ORF189P KTNKVVFIIDECHRSQFGDMHRLVKQHFKNAQYFGFTGTPRFPENSSQDGRTTADIFGRC 420

SauMWORF169P KTNKVVFIIDECHRSQFGDMHRLVKQHFKNAQYFGFTGTPRFPENSSQDGRTTADIFGRC 420

************************************************************

SauN315ORF189P LHTYLIRDAIHDGNVLGFSVDYINTFKNKALKAEDNSMVEAIDTEEVWLADKRVELVTRH 480

SauMWORF169P LHTYLIRDAIHDGNVLGFSVDYINTFKNKALKAEDNSMVEAIDTEEVWLADKRVELVTRH 480

************************************************************

SauN315ORF189P IINNHDKYTRNRQYSSIFTVQSIHALIKYYETFKRLNKKLEQPLTIAGIFTFKPNEDDRD 540

SauMWORF169P IINNHDKYTRNRQYSSIFTVQSIHALIKYYETFKRLNKKLEQPLTIAGIFTFKPNEDDRD 540

*********************************************:**************

SauN315ORF189P GEVPYHSREKLEIMISDYNKKFETNFSTDTTNEYFNHISKNVKKGVKDSKIDILIVVNMF 600

SauMWORF169P GEVPYHSREKLEIMISDYNKKFETNFSTDTTNEYFNHISKNVKKGVKDSKIDILIVVNMF 600

************************************************************

SauN315ORF189P LTGFDSKVLNTLYVDKNLMYHDLIQAYSRTNRVEKESKPFGKIVNYRDLKKETDDALRVF 660

SauMWORF169P LTGFDSKVLNTLYVDKNLMYHDLIQAYSRTNRVEKESKPFGKIVNYRDLKKETDDALRVF 660

************************************************************

SauN315ORF189P SQTNDTDTILMRSYEEYKKEFMDAYRELKMIVPTPHMVDDIQDEEELKRFVEAYRLLAKI 720

SauMWORF169P SQTNDTDTILMRSYEEYKKEFMDAYRELKMIVPTPHMVDDIQDEEELKRFVEAYRLLAKI 720

************************************************************

SauN315ORF189P ILRLKAFDEFEFTIDEIGMDEQENEDYKSKYLAVYDQVKRATAEKNKVSILNDIDFEIEM 780

SauMWORF169P ILRLKAFDEFEFTIDEIGMDEQENEDYKSKYLAVYDQVKRATAEKNKVSILNDIDFEIEM 780

************************************************************

SauN315ORF189P MRNDTINVNYIMNILRQIDLEDKAEQRRNQEQIRRILDHADDPTLRLKRDLIREFIDNVV 840

SauMWORF169P MRNDTINVNYIMNILRQIDLEDKAEQRRNQEQIRRILDHADDPTLRLKRDLIREFIDNVV 840

************************************************************

SauN315ORF189P PSLNKDDDIDQEYVNFESIKKEAEFKGFAGERSIDEQALKTISNDYQYSGVVNPHHLKKM 900

SauMWORF169P PSLNKDDDIDQEYVNFESIKKEAEFKGFAGERSIDEQALKTISNDYQYSGVVNPHHLKKM 900

************************************************************

SauN315ORF189P IGDLPLKEKRKARKAIESFVAETTEKYGV 929

SauMWORF169P IGDLPLKEKRKARKAIESFVAETTEKYGV 929

*****************************

Supplementary Figure S2. Alignment of M subunit amino acid sequences from lineages CC1 and CC5. All clones start at sequence MSITE giving 518 aa in total including the Met.

CLUSTAL 2.0.12 multiple sequence alignment

CC1-1_ -------------------------------KFHHQHQNSQHRNIPKCYNKSITQRDKLL 29

CC5-1_ -------------------------------KFHHQHQNSPHRNITKCYNKSITQRDKLL 29

CC1-2_ ------MILKAFESYNISIKFFNNNCATKTQNFHHQHPNYQHRNITKCYNKSITQRDKLL 54

CC5-2_ ------MILKAFESYNISIKFFNNNCATKTQNFHHQHPNYQHRNITKCYNKSITQRDKLL 54

:*:::* * ****.**************

CC1-1_ IQRRRNH MSITEKQRQQQAELHKKLWSIANDLRGNMDASEFRNYILGLIFYRFLSEKAEQ 89

CC5-1_ IQRRRNH MSITEKQRQQQAELHKKLWSIANDLRGNMDASEFRNYILGLIFYRFLSEKAEQ 89

CC1-2_ MQRRRNH MSITEKQRQQQAELHKKLWSIANDLRGNMDASEFRNYILGLIFYRFLSEKAEQ 114

CC5-2_ MQRRRNH MSITEKQRQQQAELHKKLWSIANDLRGNMDASEFRNYILGLIFYRFLSEKAEQ 114

:****** *****************************************************

CC1-1_ EYADALAGEDITYQEAWADEEYREDLKAELIDQVGYFIEPQDLFSAMIREIETQDFDIEH 149

CC5-1_ EYADALSGEDITYQEAWADEEYREDLKAELIDQVGYFIEPQDLFSAMIREIETQDFDIEH 149

CC1-2_ EYADALSGEDITYQEAWADEEYREDLKAELIDQVGYFIEPQDLFSAMIREIETQDFDIEH 174

CC5-2_ EYADALSGEDITYQEAWADEEYREDLKAELIDQVGYFIEPQDLFSAMIREIETQDFDIEH 174

******:*****************************************************

CC1-1_ LATAIRKVETSTLGEESENDFIGLFSDMDLSSTRLGNNVKERTALISKVMVNLDDLPFVH 209

CC5-1_ LATAIRKVETSTLGEESENDFIGLFSDMDLSSTRLGNNVKERTALISKVMVNLDDLPFVH 209

CC1-2_ LATAIRKVETSTLGEESENDFIGLFSDMDLSSTRLGNNVKERTALISKVMVNLDDLPFVH 234

CC5-2_ LATAIRKVETSTLGEESENDFIGLFSDMDLSSTRLGNNVKERTALISKVMVNLDDLPFVH 234

************************************************************

CC1-1_ SDMEIDMLGDAYEFLIGRFAATAGKKAGEFYTPQQVSKILAKIVTDGKDKLRHVYDPTCG 269

CC5-1_ SDMEIDMLGDAYEFLIGRFAATAGKKAGEFYTPQQVSKILAKIVTDGKDKLRHVYDPTCG 269

CC1-2_ SDMEIDMLGDAYEFLIGRFAATAGKKAGEFYTPQQVSKILAKIVTDGKDKLRHVYDPTCG 294

CC5-2_ SDMEIDMLGDAYEFLIGRFAATAGKKAGEFYTPQQVSKILAKIVTDGKDKLRHVYDPTCG 294

************************************************************

CC1-1_ SGSLLLRVGKETQVYRYFGQERNNTTYNLARMNMLLHDVRYENFDIRNDDTLENPAFLGH 329

CC5-1_ SGSLLLRVGKETQVYRYFGQERNNTTYNLARMNMLLHDVRYENFDIRNDDTLENPAFLGN 329

CC1-2_ SGSLLLRVGKETQVYRYFGQERNNTTYNLARMNMLLHDVRYENFEIRNDDTLENPAFLGN 354

CC5-2_ SGSLLLRVGKETQVYRYFGQERNNTTYNLARMNMLLHDVRYENFEIRNDDTLENPAFLGN 354

********************************************:**************:

CC1-1_ TFDAVIANPPYSAKWTADSKFENDERFSGYGKLAPKSKADFAFIQHMVHYLDDEGTMAVV 389

CC5-1_ TFDAVIANPPYSAKWTADSKFENDERFSGYGKLAPKSKADFAFIQHMVHYLDDEGTMAVV 389

CC1-2_ TFDAVIANPPYSAKWTADSKFENDERFSGYGKLAPKSKADFAFIQHMVHYLDDEGTMAVV 414

CC5-2_ TFDAVIANPPYSAKWTADSKFENDERFSGYGKLAPKSKADFAFIQHMVHYLDDEGTMAVV 414

****************************************************************

CC1-1_ LPHGVLFRGAAEGVIRRYLIEEKNYLEAVIGLPANIFYGTSIPTCILVFKKCRQQEDYVL 449

CC5-1_ LPHGVLFRGAAEGVIRRYLIEEKNYLEAVIGLPVNIFYGTSIPTCILVFKKCRQQDDNVL 449

CC1-2_ LPHGVLFRGAAEGVIRRYLIEEKNYLEAVIGLPANIFYGTSIPTCILVFKKCRQQDDNVL 474

CC5-2_ LPHGVLFRGAAEGVIRRYLIEEKNYLEAVIGLPANIFYGTSIPTCILVFKKCRQQDDNVL 474

*********************************.*********************:* **

CC1-1_ FIDASNDFEKGKNQNHLTDAQVERIINTYKRKETIDKYSYSATLQEIADNDYNLNIPRYV 509

CC5-1_ FIDASNDFEKGKNQNHLSDAQVERIIDTYKRKETIDKYSYSATLQEIADNDYNLNIPRYV 509

CC1-2_ FIDASNDFEKGKNQNHLSDAQVERIIDTYKRKATIDKYSYSATLQEIADNDYNLNIPRYV 534

CC5-2_ FIDASNDFEKGKNQNHLSDAQVERIIDTYKRKATIDKYSYSATLQEIADNDYNLNIPRYV 534

*****************:********.***** ***************************

CC1-1_ DTFEEEAPIDLDQVQQDLKNIDKEIAEVEQEINAYLKELGVLKDE 554

CC5-1_ DTFEEEAPIDLDQVQQDLKNIDKEIAEIEQEINAYLKELGVLKDE 554

CC1-2_ DTFEEEAPIDLDQVQQDLKNIDKEIAEIEQEINAYLKELGVLKDE 579

CC5-2_ DTFEEEAPIDLDQVQQDLKNIDKEIAEIEQEINAYLKELGVLKDE 579

***************************:*****************

Supplementary Figure S3. A CLUSTAL alignment of the amino acid sequences of the R subunit from *S. aureus* N315 (CC5) with the R subunit of the Type IC enzyme, EcoR124I. The green shaded box indicates the motif essential for DNA cleavage. The red shaded boxes indicate the motifs essential for the ATPase-driven DNA translocation function associated with R subunits.


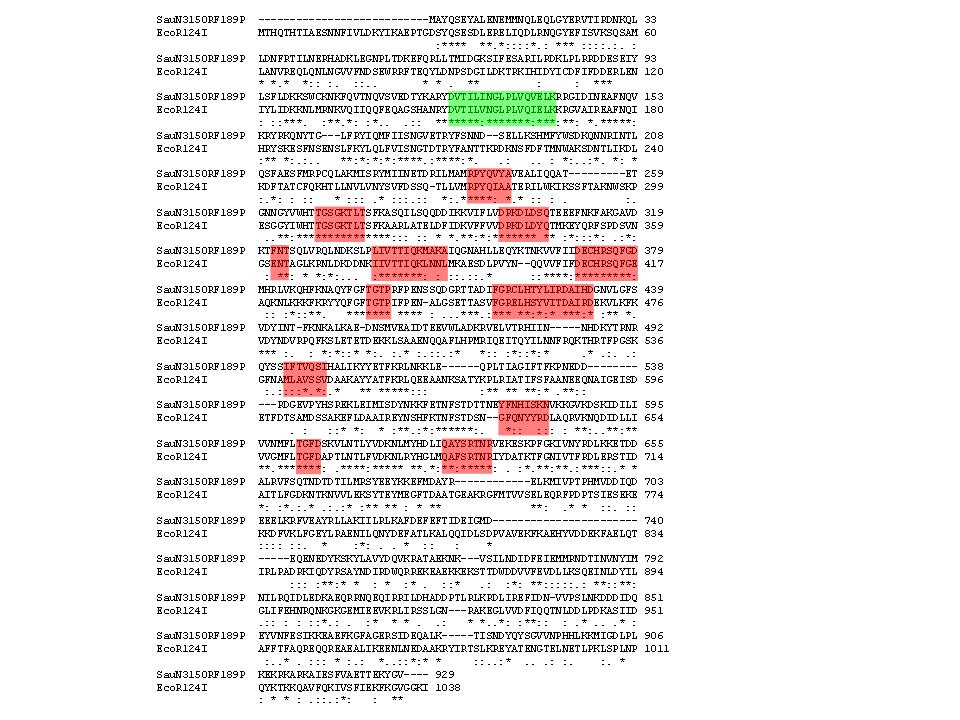


Supplementary Figure S4. A CLUSTAL alignment of the M subunit of the CC5-1 Sau1 system with the M subunit of the EcoR124I Type IC system. The red shaded boxes indicate the location of the conserved motifs for S-adenosyl methionine binding and methyltransferase activity.


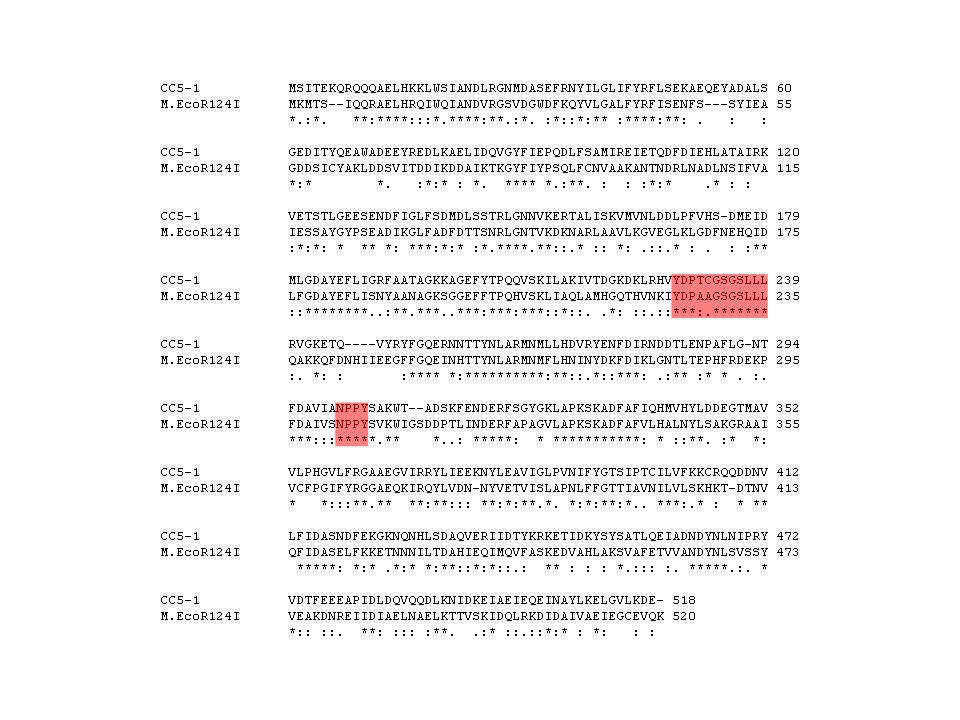


Supplementary Figure S5. A CLUSTAL alignment of the four S subunit sequences examined in this work. The red shaded regions show the conserved regions. The coloured letters show the TRD sequences. The first TRD is conserved in three sequences (blue) but not in the fourth (red). The second TRD is not conserved in any of the systems and is shown in four different colours (grey, green, magenta and orange). The initial methionine residue shown in lower case is assumed to be the translational start.


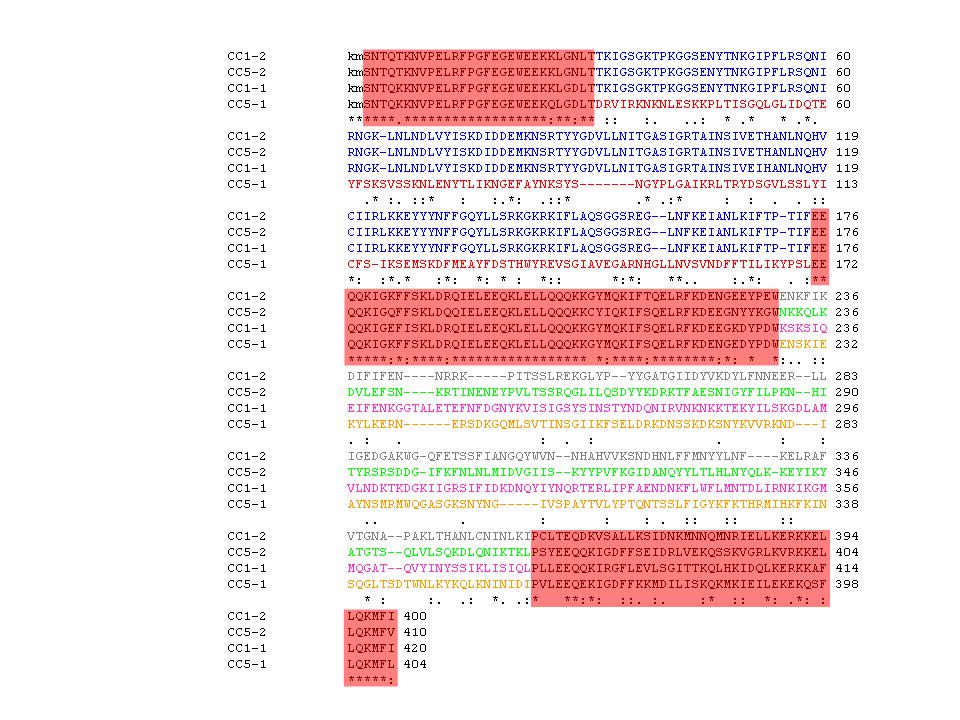


**Supplementary Table T1.** *S. aureus* whole-genomes (*n*=18), plasmids (*n*=233), bacteriophage (*n*=50) and Staphylococcal cassette chromosomes with *mecA* (*n*=35) sequences analyzed to determine target site distribution.

| **Whole-genome (strain)** | **GenBank Accession number** | **Size (bp)** |
| --- | --- | --- |
| MSSA476 | BX571857 | 2799802 |
| MW2 | BA000033 | 2820462 |
| ED98 | CP001781 | 2824404 |
| Mu3 | AP009324 | 2880168 |
| Mu50 | BA000017 | 2878529 |
| N315 | BA000018 | 2814816 |
| JH1 | CP000736 | 2906507 |
| JH9 | CP000703 | 2906700 |
| NCTC 8325 | CP000253 | 2821361 |
| Newman | AP009351 | 2878897 |
| USA300 FPR3757 | CP000255 | 2872769 |
| USA300 TCH1516 | CP000730 | 2872915 |
| COL | CP000046 | 2809422 |
| EMRSA15/5096 | NC_017763 | 2832299 |
| MRSA252 | BX571856 | 2902619 |
| RF122/ET3-1 | AJ938182 | 2742531 |
| 0582/TW20 | FN433596 | 3043210 |
| S0385 | AM990992 | 2872582 |
|  |  |  |
| **Plasmid (name)** | **GenBank Accession number** | **Size (bp)** |
| pRJ9 | AF447813 | 10406 |
| pKH16 | NC_010262 | 4442 |
| pUSA02 | NC_007791 | 4439 |
| pKH17 | NC_010284 | 4441 |
| SAP060B | GQ900417 | 4498 |
| pT181 | NC_006629 | 4440 |
| pJ3358 | NC_001763 | 6024 |
| M899 | NZ_GG730190 | 4368 |
| C101 | NZ_GG730120 | 4278 |
| pNS1 | PNS1CG | 3879 |
| pKH6 | SAU38428 | 4439 |
| SAP085A | GQ900437 | 4439 |
| SAP095A | GQ900445 | 4439 |
| SAP094A | GQ900443 | 4439 |
| MR1 | NZ_ACZQ01000139 | 4522 |
| A8115 | NZ_ACKG01000002 | 4498 |
| A9754 | NZ_ADJI01000035 | 4947 |
| ATCC BAA-39 | NZ_AEEK01000040 | 4457 |
| 58-424_1 | NZ_GG749054 | 5143 |
| SAP093A | GQ900441 | 4439 |
| pS0385-1 | AM990993 | 5246 |
| pC223 | AY355285 | 4608 |
| pC221 | NC_006977 | 4555 |
| pKH7 | NC_002096 | 4118 |
| pTZ4 | NC_010111 | 4555 |
| pSBK203 | SAU35036 | 3780 |
| SAP084A | GQ900436 | 4595 |
| pS0385-2 | AM990994 | 4381 |
| pS194 | NC_005564 | 4397 |
| pVGA | NC_011605 | 5713 |
| pAVY | NC_013451 | 1442 |
| pWBG751 | GQ900393 | 2473 |
| SAP078B | GQ900431 | 2415 |
| pWBG738 | NC_007209 | 2473 |
| pKH20 | NC_010686 | 2412 |
| pKH19 | NC_010685 | 2473 |
| TCH130 | NZ_ACHD01000266 | 2476 |
| A9719 | NZ_ACKJ01000014 | 2402 |
| A6300 | NZ_ACKF01000018 | 2366 |
| A9765 | NZ_ACSN01000068 | 2399 |
| TCH70_1 | NZ_ACHH02000015 | 2300 |
| pE5 | PE5PE5A | 2473 |
| TCH959 | NZ_AASB02000192 | 2476 |
| CF-Marseille | NZ_CABA01000093 | 2473 |
| pT48 | NC_001395 | 2475 |
| pSK3 | NC_001994 | 1658 |
| pSK6 | NC_001995 | 1551 |
| SAP104B | GQ900451 | 1552 |
| pSK17 | GQ900513 | 9968 |
| SAP065A | GQ900420 | 2908 |
| pDLK2 | GU562625 | 2908 |
| pWBG1773 | NC_010616 | 2916 |
| SAP080A | GQ900433 | 43892 |
| SAP069A | GQ900422 | 42198 |
| SAP068A | GQ900421 | 50500 |
| pUSA03 | NC_007792 | 37136 |
| p18806-P03 | CP002134 | 27068 |
| p18807-P03 | CP002135 | 26972 |
| p18809-P03 | CP002139 | 27068 |
| pLAC-P03 | CP002149 | 27068 |
| p18808-P03 | CP002137 | 27059 |
| p18811-03 | CP002143 | 27070 |
| SAP049A | GQ900407 | 25022 |
| SAP015A | GQ900380 | 27068 |
| SAP050A | GQ900409 | 27067 |
| p18805-03 | CP002132 | 27069 |
| SAP027A | GQ900388 | 29646 |
| SAP051A | GQ900410 | 23059 |
| SAP046A | GQ900403 | 27068 |
| pSJH901 | NC_009477 | 30429 |
| pSJH101 | NC_009619 | 34029 |
| A8796 | NZ_ADJJ01000025 | 17758 |
| pBORa53 | NC_013550 | 17334 |
| TCH60 | NZ_ACHC01000043 | 24490 |
| pUB101 | NC_005127 | 21845 |
| pLEW6932 | NC_009130 | 51514 |
| VRSAp | NC_002774 | 25107 |
| pWBG748 | GQ915265 | 44964 |
| pWBG749 | GQ900391 | 38087 |
| pI258 | GQ900378 | 29254 |
| 65-1322 | NZ_GG700565 | 22093 |
| 68-397 | NZ_GG700590 | 27097 |
| E1410 | NZ_GG700609 | 23259 |
| M876 | NZ_GG700623 | 23124 |
| C101 | NZ_GG730140 | 24019 |
| 58-424_3 | NZ_GG749073 | 24634 |
| M809 | NZ_GG749324 | 23537 |
| pSK76 | GQ900444 | 23983 |
| M1015 | NZ_GG749015 | 23501 |
| pTW20_1 | FN433597 | 29585 |
| pSK62 | GQ900446 | 22694 |
| SAP055A | GQ900414 | 20453 |
| pUSA300HOUMS | NC_010066 | 20413 |
| SAP017A | GQ900382 | 32650 |
| SAP057A | GQ900415 | 39308 |
| pKH8 | SAU50077 | 2417 |
| pNVH99 | AJ296103 | 2239 |
| pSA1308 | NC_007928 | 2756 |
| pKH4 | SAU81980 | 2487 |
| pWBG754 | GQ900396 | 2241 |
| pKH21 | NC_010684 | 2531 |
| pBMSa1 | AY541446 | 2750 |
| pC55s | AY048756 | 3333 |
| pKH18 | NC_010231 | 3332 |
| pNVH01 | NC_004562 | 2650 |
| pUSA01 | NC_007790 | 3125 |
| pKH12 | NC_010687 | 3011 |
| pKH14 | NC_010428 | 3124 |
| pUSA10HOUMR0001 | NC_012417 | 3125 |
| pS0385-3 | AM990995 | 3158 |
| SAP049B | GQ900408 | 3125 |
| pKH3 | NC_005020 | 2979 |
| pTW20_2 | FN433598 | 3011 |
| pWBG760 | GQ900473 | 4412 |
| SAP051B | GQ900411 | 3125 |
| SAP046B | GQ900404 | 3125 |
| p18805-P01 | CP002133 | 3125 |
| p18807-P01 | CP002136 | 3125 |
| p18808-P01 | CP002138 | 3125 |
| p18809-P01 | CP002140 | 3125 |
| p18810-P01 | CP002142 | 3125 |
| pLAC-P01 | CP002150 | 3125 |
| p18811-P01 | CP002144 | 3125 |
| SAP070A | GQ900423 | 3011 |
| SAP104A | GQ900450 | 3011 |
| A5948_3 | NZ_ACKD01000027 | 2562 |
| pKKS832 | FN377602 | 6242 |
| CF-Marseille_2 | NZ_CABA01000045 | 4556 |
| MR1 | NZ_ACZQ01000138 | 4604 |
| A9781_2 | NZ_ACKL01000028 | 4554 |
| A9763 | NZ_ACKK01000035 | 4555 |
| A9719_3 | NZ_ACKJ01000060 | 4555 |
| A6300_2 | NZ_ACKF01000048 | 4554 |
| A6244 | NZ_ACKE01000028 | 4555 |
| A5948_4 | NZ_ACKD01000047 | 4379 |
| A5937 | NZ_ACKC01000002 | 4556 |
| A10102 | NZ_ACSO01000036 | 4554 |
| JKD6009 | NZ_ABSA01000025 | 4548 |
| pKH13 | NC_010426 | 2909 |
| pKH15 | NC_010427 | 2907 |
| SAP087A | GQ900439 | 2396 |
| pWBG764 | GQ900468 | 2397 |
| pE194 | NC_005908 | 3728 |
| SAP085B | GQ900438 | 3961 |
| SAP099B | GQ900449 | 16428 |
| pETB | NC_003265 | 38211 |
| SAP102A | GQ900496 | 36082 |
| SAP077A | GQ900428 | 35510 |
| SAP067A | GQ900483 | 37206 |
| TCH70_2 | NZ_ACHH02000018 | 18140 |
| pWBG750 | GQ900392 | 20653 |
| pWBG757 | GQ900397 | 20730 |
| pWBG763 | GQ900467 | 20730 |
| p18809-P04 | CP002146 | 28404 |
| USA300_TCH959 | NZ_AASB02000125 | 14600 |
| ATCC 51811 | NZ_ADVP01000034 | 16164 |
| SAP072A | GQ900424 | 20653 |
| SAP073A | GQ900425 | 20729 |
| SAP053A | GQ900413 | 20672 |
| pSaa6159 | CP002115 | 20730 |
| p21 | NC_002517 | 20719 |
| pMW2 | NC_005011 | 20654 |
| pSAS | BX571858 | 20652 |
| SAP054A | GQ900477 | 37475 |
| SAP078A | GQ900430 | 35508 |
| SAP075A | GQ900486 | 37050 |
| SAP076A | GQ900427 | 35114 |
| SAP019A | GQ900385 | 27435 |
| A9635 | NZ_ACKI01000002 | 24653 |
| pWBG752 | GQ900394 | 24654 |
| pWBG755 | GQ900471 | 25607 |
| pWBG744 | GQ900398 | 27268 |
| SAP012A | GQ900377 | 27267 |
| SAP074A | GQ900426 | 27268 |
| A8819 | NZ_ADJK01000020 | 27798 |
| A9781 | NZ_ACKL01000034 | 27268 |
| SAP063A | GQ900418 | 26016 |
| p19231-P03 | CP002147 | 27425 |
| pLUH02 | FR714929 | 27271 |
| SAP060A | GQ900416 | 22987 |
| A8115 | NZ_ACKG01000021 | 22988 |
| A8117 | NZ_ACYO01000019 | 23292 |
| SAP058A | GQ900479 | 22439 |
| pWBG756 | GQ900472 | 24456 |
| pWBG761 | GQ900474 | 26838 |
| SAP071A | GQ900485 | 26883 |
| pN315 | NC_003140 | 24653 |
| pTZ2162 | NC_010419 | 35380 |
| pSA1379 | NC_007931 | 22787 |
| D139 | GG730186 | 33841 |
| SAP052A | GQ900412 | 32445 |
| pUSA300HOUMR | NC_010063 | 27041 |
| SAP082A | GQ900434 | 44116 |
| SAP015B | GQ900500 | 21860 |
| pSK41 | AF051917 | 46445 |
| pGO1 | NC_012547 | 54000 |
| pLW043 | NC_005054 | 57889 |
| SAP079A | GQ900432 | 47322 |
| SAP065B | GQ900481 | 26750 |
| SAP066A | GQ900482 | 32122 |
| SAP064A | GQ900419 | 30857 |
| SAP048A | GQ900406 | 27268 |
| SAP070B | GQ900484 | 29532 |
| pCM05 | GQ900387 | 33660 |
| pSK21 | GQ900490 | 37046 |
| pSK57 | GQ900493 | 31164 |
| SAP101A | GQ900495 | 32163 |
| pSK53 | GQ915270 | 38886 |
| pSK23 | GQ900491 | 41993 |
| pSK80 | GQ900492 | 31314 |
| pSK74 | GQ915266 | 35327 |
| pSK60 | GQ915267 | 35175 |
| pSK79 | GQ900489 | 35859 |
| SAP104C | GQ900498 | 29343 |
| pWBG753 | GQ900395 | 30047 |
| SAP103A | GQ900497 | 29258 |
| pSK156 | GQ900448 | 45052 |
| pWBG747 | GQ900399 | 33701 |
| pSK59 | GQ900488 | 29570 |
| pSK64 | GQ915268 | 34784 |
| pWBG746 | GQ900390 | 33702 |
| pEDINA | NC_010077 | 34986 |
| SAP059A | GQ900480 | 26243 |
| SAP056A | GQ900478 | 27128 |
| pWBG759 | GQ900401 | 28384 |
| pSK77 | GQ900494 | 27694 |
| WBG10049 | NZ_GG730219 | 24446 |
| pWBG762 | GQ900475 | 54023 |
| SAP014A | GQ900379 | 50429 |
| pPR9 | GU237136 | 41715 |
| p18813-P03 | CP002145 | 30127 |
| pV030-8 | EU366902 | 39041 |
| pI5S5 | HE579068 | 37285 |
|  |  |  |
| **Bacteriophage(name)** | **GenBank Accession number** | **Size (bp)** |
| PT1028 | AY954948 | 15603 |
| phiMR1 | AB370268 | 43011 |
| 66 | AY954949 | 18199 |
| 187 | AY954950 | 39620 |
| phiNM1 | DQ530359 | 40000 |
| P954 | GQ398772 | 40761 |
| SAP-26 | GU477322 | 41207 |
| 2638A | AY954954 | 41318 |
| PVL | AB009866 | 41401 |
| 52A | AY954965 | 41690 |
| TEM126 | HQ127381 | 41882 |
| 55 | AY954963 | 41902 |
| 92 | AY954967 | 42431 |
| phi5967PVL | AP011955 | 42461 |
| phi7247PVL | AP011956 | 42481 |
| phiSauS-IPLA8 | EU861004 | 42526 |
| phi 13 | AF424783 | 42722 |
| 69 | AY954951 | 42732 |
| 29 | AY954964 | 42802 |
| phiSL | AB045978 | 42942 |
| 3A | AY954956 | 43095 |
| 71 | AY954962 | 43114 |
| phiNM2 | DQ530360 | 43145 |
| ROSA | AY954961 | 43155 |
| 88 | AY954966 | 43231 |
| phiETA2 | AP008953 | 43265 |
| phiETA3 | AP008954 | 43282 |
| X2 | AY954968 | 43440 |
| 96 | AY954960 | 43576 |
| phi 11 | AF424781 | 43604 |
| 37 | AY954958 | 43681 |
| TEM123 | JQ779024 | 43786 |
| 80alpha | DQ517338 | 43864 |
| 53 | AY954952 | 43883 |
| phiNM3 | DQ530361 | 44061 |
| 85 | AY954953 | 44283 |
| phiMR25 | AB370205 | 44342 |
| 47 | AY954957 | 44777 |
| phiPVL108 | AB243556 | 44857 |
| phi 12 | AF424782 | 44970 |
| EW | AY954959 | 45286 |
| 42e | AY954955 | 45861 |
| Sb-1 | HQ163896 | 127188 |
| K | AY176327 | 127395 |
| Twort | AY954970 | 130706 |
| SA11 | JX194239 | 136326 |
| A5W | EU418428 | 137083 |
| ISP | FR852584 | 138339 |
| G1 | AY954969 | 138715 |
| GH15 | JQ686190 | 139806 |
|  |  |  |
| **SCC*mec* (representative strain)** | **GenBank Accession number** | **Size (bp)** |
| NN1 | AB245470 | 21770 |
| JCSC1978(8/6-3P) | AB063173 | 21777 |
| 80s-2 | AB245471 | 21801 |
| JCSC6668 | AB425823 | 23124 |
| M03-68 | DQ106887 | 23157 |
| HDE288 | AF411935 | 23293 |
| HDE288 | AF411935 | 23293 |
| JCSC6670 | AB425824 | 23328 |
| AR43/3330 | AJ810121 | 23600 |
| 2314 | AY271717 | 24106 |
| WKZ-2 | GQ918137 | 24503 |
| CA05 | AB063172 | 26090 |
| JCSC6833 | AB435013 | 26707 |
| AR13.1/3330.2 | AJ810120 | 27029 |
| M299 | HM030721 | 27379 |
| WIS | AB121219 | 28612 |
| 81/108(MR108) | AB096217 | 31263 |
| CF-Marseille | AM943017 | 32797 |
| JCSC6082 | AB373032 | 33261 |
| C10682 | FJ390057 | 33371 |
| BK20781 | FJ670542 | 33743 |
| ZH47 | AM292304 | 34675 |
| NN50 | AB633329 | 35517 |
| NCTC10442 | AB033763 | 39332 |
| BK16704 | GU235984 | 40424 |
| TSGH17 | AB512767 | 42083 |
| JCSC 5952 | AB478780 | 42430 |
| JCSC6943 | AB505628 | 44355 |
| JCSC6944 | AB505629 | 47212 |
| PM1 | AB462393 | 49137 |
| Sa0059 | JQ412578 | 50265 |
| 45394F | GU122149 | 51121 |
| M1 | HM030720 | 53863 |
| N315 | D86934 | 58237 |
| 85/2082 | AB037671 | 68256 |

**Supplementary Table T2.** Cleavage results showing only those plasmids which were cut to a linear form (shown as Y) or not cut (shown as N). CC1-2 linearized pUC19 and hence could not be used in closed circular form for analysis (so all other plasmids are shown as – for not done). However, linearizing the plasmids with EcoRI or BamHI prior to cleaving with CC1-2 resulted in a smear being formed if two or more sites for CC1-2 were present on the linearised plasmid (shown as Y).

| **PLASMID** | **Sau1 enzyme** | | | | |
| --- | --- | --- | --- | --- | --- |
|  | **CC1-1** | **CC1-2** | **CC1-2 on LINEAR dna** | **CC5-1** | **CC5-2** |
| **pUC19** | **N** | **y** | **-** | **n** | **N** |
| **Clone1** | **Y** | **-** | **N** | **Y** | **Y** |
| **Clone2** | **N** | **-** | **N** | **Y** | **N** |
| **Clone4** | **N** | **-** | **N** | **N** | **N** |
| **Clone5** | **Y** | **-** | **N** | **Y** | **Y** |
| **Clone6** | **N** | **-** | **Y** | **Y** | **Y** |
| **Clone7** | **N** | **-** | **Y** | **Y** | **N** |
| **Clone9** | **N** | **-** | **N** | **Y** | **Y** |
| **Clone10** | **Y** | **-** | **N** | **Y** | **Y** |
| **Clone11** | **N** | **-** | **N** | **Y** | **Y** |
| **Clone12** | **Y** | **-** | **N** | **N** | **N** |
| **Clone13** | **N** | **-** | **N** | **Y** | **N** |
| **Clone14** | **N** | **-** | **Y** | **N** | **Y** |
| **Clone15** | **N** | **-** | **Y** | **Y** | **N** |
| **Clone16** | **N** | **-** | **N** | **Y** | **N** |
| **Clone17** | **N** | **-** | **N** | **Y** | **Y** |
| **Clone18** | **N** | **-** | **N** | **N** | **Y** |
| **Clone19** | **N** | **-** | **N** | **Y** | **N** |
| **Clone20** | **N** | **-** | **N** | **Y** | **N** |
| **PhiED1clone5/I** | **N** | **-** | **-** | **N** | **N** |
| **PhiED1clone5/II** | **N** | **-** | **-** | **N** | **N** |
| **PhiED1clone5/III** | **N** | **-** | **-** | **N** | **N** |
| **PhiED1clone5/IV** | **N** | **-** | **-** | **N** | **N** |
| **PhiED1clone5/V** | **Y** | **-** | **-** | **Y** | **N** |
| **PhiED1clone5/VI** | **N** | **-** | **-** | **N** | **N** |
| **PhiED1clone5/VII** | **N** | **-** | **-** | **N** | **N** |
| **PhiED1clone5/VIII** | **N** | **-** | **-** | **N** | **N** |
| **PhiED1clone5/IX** | **N** | **-** | **-** | **N** | **Y** |
| **PhiED1clone5/X** | **N** | **-** | **-** | **N** | **N** |
| **PhiED1clone14/I** | **-** | **-** | **-** | **-** | **N** |
| **PhiED1clone14/II** | **-** | **-** | **-** | **-** | **N** |
| **PhiED1clone14/III** | **-** | **-** | **-** | **-** | **N** |
| **PhiED1clone14/IV** | **-** | **-** | **-** | **-** | **N** |
| **PhiED1clone14/V** | **-** | **-** | **-** | **-** | **N** |
| **PhiED1clone14/VI** | **-** | **-** | **-** | **-** | **N** |
| **PhiED1clone14/VII** | **-** | **-** | **-** | **-** | **N** |
| **PhiED1clone14/VIII** | **-** | **-** | **-** | **-** | **N** |
| **PhiED1clone14/IX** | **-** | **-** | **-** | **-** | **Y** |
| **PhiED1clone14/X** | **-** | **-** | **-** | **-** | **N** |
| **5/V/A** | **Y** | **-** | **-** | **N** | **-** |
| **5/V/B** | **Y** | **-** | **-** | **N** | **-** |
| **5/V/C** | **N** | **-** | **-** | **Y** | **-** |
| **5/V/D** | **N** | **-** | **-** | **Y** | **-** |
| **5/V/E** | **N** | **-** | **-** | **N** | **-** |
| **5/V/F** | **N** | **-** | **-** | **N** | **-** |
| **5/IX/A** | **-** | **-** | **-** | **-** | **Y** |
| **5/IX/B** | **-** | **-** | **-** | **-** | **N** |
| **5/IX/C** | **-** | **-** | **-** | **-** | **N** |
| **5/IX/D** | **-** | **-** | **-** | **-** | **Y** |
| **5/IX/E** | **-** | **-** | **-** | **-** | **N** |
| **5/IX/F** | **-** | **-** | **-** | **-** | **N** |
| **14/IX/A** | **-** | **-** | **-** | **-** | **N** |
| **14/IX/B** | **-** | **-** | **-** | **-** | **N** |
| **14/IX/C** | **-** | **-** | **-** | **-** | **N** |
| **14/IX/D** | **-** | **-** | **-** | **-** | **N** |
| **14/IX/E** | **-** | **-** | **-** | **-** | **N** |
| **14/IX/F** | **-** | **-** | **-** | **-** | **Y** |
| **pY4** | **N** | **-** | **-** | **N** | **N** |
| **pY33** | **N** | **-** | **-** | **Y** | **N** |
| **pY107** | **N** | **-** | **-** | **N** | **N** |
| **pY124** | **Y** | **-** | **-** | **Y** | **N** |
| **pY225** | **N** | **-** | **-** | **Y** | **N** |
| **pY378** | **N** | **-** | **-** | **N** | **N** |
| **pY401** | **N** | **-** | **-** | **Y** | **N** |
| **pY493** | **N** | **-** | **-** | **N** | **Y** |
| **pY524** | **N** | **-** | **-** | **Y** | **N** |
| **pY627** | **N** | **-** | **-** | **Y** | **Y** |
| **pY667** | **Y** | **-** | **-** | **N** | **Y** |

**Supplementary materials and methods.**

**a. Approximately 2.4kb PhiED1 segments.** These sequences were amplified by PCR from DNA extracted from *Bacteroides fragilis* phage PhiED1 (kindly supplied by Dr Garry Blakely, Edinburgh), employing oligonucleotides with integral restriction sites, and ligated in pUC19 as EcoRI-BamHI fragments. PhiED1clone3 was planned but could not be constructed.

**Clone1**

GTGTTTCTCGAAAACAGGCAAAATCTTTAATGAAAGGGTTCTTGTTACTATGGGAATAGATATTATTTGCGCAATTGATCCCGGTGTGTCAGCTGGTGGAATAGTGGTATATAAGCCGGGTAATAGTCTTATTACTATCCCAATGCCACGCACGGCAAAGGGTATTTTTAACGTGTTTCAAAAAGTGAAGCGTTCCGGTAGCCCTGCAATATTCATTGAGCGTCTTTCGGTTCGTGGGGGTGACTCCGGAGGCGGGAAAGAATTTAGAATAGCAACTATGTTGGAGAACTACAACTACCTTGTATGTTGTGCGCTCGTTCTTGATATTCCTTTATTTCTGTGTGCGCCTATTTCGTGGCAAAGTGGTTTAAATCTGAGGGAGAAAGGAGAGAAAGAGGAAAAGAAGGATAGAAAAGAAAAGTATCTGAATTATGCGATGAAGCAATTCCCGCTTGCAAACGTGAAATTATGGAATAGTGACGCTATATGTATTTTGCGCTTCGCACAGATGAAGATGATTTGTGATGTCGATTGGTTTTCAAGTAACATGCAGAACGAAAACAGCACAGAAATAGCATTTTCTCCCCCTCTGTTGGACGATAGTATTAAATTCGTAGAAAGATATGGATTCAAAAGAAAACGATCTAAAAAACGCTCTAATTGAATCGGTGAAAGAATTGAGAAGCGCACAGAAGAGATTTGAGCGATTCGGGGAGAGATACAGAGAGAGAAAAGAAAAGGCGGAAAAGAAAGTAGATGATATAATTTCTAAGCTGACGGACACGCAAATAAGTTTATTTTAATTTATTAACTGTTTGTCTATTGCTATGTTCGATTAAGTGCGTATATTTGCAGTGTAAAATTTGTCCGCCAACAAATTTAAAGATATTGCTTAATAGCGTTAATGCCTCGGTTCGTGTTGTTTGGCGGCACACGAATCGGGGCATTTAATTTTAAACTGTAATGATATGGAAATTTCGGTGATTGATCAGAGAGAAGTTTTAGGAGTTGATTTTAAAATATATGGTACTTTTGATGATCCTTTGTTTTTGGCTAAAGATGTTGCTATTTGTATTGATTATGATTTGAGTAGCGTTAATAAACTGGTGAACCTTGTAGATGATAATGAAAAGGTACGGAACATTATTCCGACACTTGGAGGTAATCAAGAAGCTTGGTTTTTAACTGAAGATGGTTTATACGAGGTGTTAATGCTAAGTAGAAAACCTATGGCAAAAGCATTTAAATCAAAAATAAAAGAGATATTGAAAGATATACGCAGACATGGTATATATGCAACTGATAATGTTATAGATCAGATATTAAATAACCCAGATTTTGGAATAGGACTATTAACTAAGTTAAAGGAGGAAAGATCGGCAAGAATAGAGGCGGAAAAGAAAAATGCAATACTTATGCACGTGAATAAAACTTATACATCTACGGAGATAGCGAAAGAGATAGGTTTAAAATCGGCAAACGAATTAAATAAAATACTCGAATCTAAGAAGATTCAGTTTAAAGTTAATGGTACATGGGTATTGTATTCTAAGTACTCTAATTGCGGTTACGAAGAAGTGAAACAAGAAGTTCTTGATAGTGGAAGGGTTATATATCATAGACGTTTCACGCAGCTTGGGAGAGATTTTATATTAAACCTATTCAAGTGATATTCTAAACTAAAGTTAAATAACGGGTATTTCGGAAAGATTTACCCGTTTTTATTTGCGTTAATTTAAAGTTTTGCTTTAATTTGCAGCGTAGAAATAAAAACAGTAGTAACAATAAAATCAATTAATTATGCAGGAAATTAACAAGAAATTAAGTGAACAGTCAGTAGAAAAGGTTTTGGATAGACCGGAGTATAGAAAAGAGCTTTCTATTTATTGGGATGGCTTAAAAGAGCAACGGAAAAAGGCTTCGTTCGAAATATTGCATCACGGAGGTATTCCTAAAAGAATAACAATAGATAGAGTAGATAAAATGGATACGGATCAACTTATATCAGAATTTAAGCTGATACTTGACAGAAAGAGTGAGTTGCCTGCAAGTCTGAGGTACTTTATTTCGGATGTGTGCGGAAAGGTATTTATTAGTTGGTTTACAAAAGTGATCGAAGATGAAGCAAAAGAAAATAACGATACCCGGGAAGGTAACTAAGGACGGTAAGTTATCCATCTACATGGGCGAGCTTAACGAGTTTATGAAGAACAACGCAGGGAAAAATATTATTGCGGAGTTTACGGTATTAGAACCGTCTGATTCTTCATCCTTGCGTGGATACTACTTTAAATACGTTGTTCCCCAATTTCAGAAAGGGATGTGCGAAAATGGGTACAGGTGGAGCGAAGAAGAAACGGAGGCTTATATGCGTAGTATTTGCCCTATTACGATGGGTGAAGTTGTAGATGTTGAAACTGGTGAGTATAG

**Clone2**

GTTGAAACTGGTGAGTATAGAAAGGACTCAGTTAAAGTTACCGATTTAAGCAATAGCGAATTTGTCGAATACATAGAATTTTTAAAGCAGTTTGCGGCAGAAGAATTTAGTATTTATATTGAAGAACCAAATAGATTTGTAAGATGAAAGAAAATGAAGAAATGACTTTAGAGGAAAAGTTCAATTTGATGTGCGAAGCATTAAGCATATCTCCGGAGAAAATTATATCACGGGATATTACACGTTATGTATCACTTCGAAGGAATTGCATTATCCATCAGCTTTACGCCTATAAAAATCACGGTTTACCCGAATTGATAGGTCGCACGAAGGTTTTAATTATGAAAGCGCATGAACGTTTTCAAGGCGAATTAGATGTGAAAGATATGACAGCCGTAGAGTTTGTACGGCTTATAGACGAACGACTGCAAAAGTATATTGATGGCAAAGAAGATTAAGAATCTTGTTCTTGTTCATTGCACGGAGTGTAGGTTCAGTTCAGATCACCATAATTTGATTTGCTATTGCAAAAAGAGAGATAAAAAGTTATGCAGTTGCCCGAACATTGGGCGGGTCTGTGAGTTTTACATTAAAAAATAAAGTATCATGTTAAAAGACAATTTTGAATTAAAGAGAGTTAAGTTCTTGAATAACGGTTTAGAGGTTGATTACAATGATTGCCGTTTGGTTGATGGTGAAGAAACAAAGACGTTTCACAAGGTAAAATGCCCCGAATATCCGCATAGAGATTTAGGAATTGCGGCAAATGAGCTTCGTTCATACATAGTTGAATTGATGGGAATAATGAATTTTAGGAACATCACCTATTTGTCTGATTTGGCAAAACAAGACAATGAGTTAAGTAGACAATTCGATGAATATTTTGAAACGCTTGCTACCCGTGTCGCCATTAGCGAGATAGTTTACGATCCCGAAAAGAATACAATCGTTTTCAAATATATTTTCGCAGGCGTAGATTTATCCCGGTTGAAAATGCAAACGAGCAAAATTATGTTGGACGGTGAAGGGTTGAAATTTGAAATAGCACTACAAGAAGATTTTGAAGCACTGAAAGATGAAATTTTCAAGTATCTTTTTGAGAATAAGCGTGCACAATTGGAGCTATTCGGTGAGACAGCAACGGCAGAACAGGACGATAGTTTGACGCCCGATGATGATTTAGAAGGTGACGATACGTTTTTTGATGATGAAGAAGCAGAGCAGCCGGAGTTGATCGAAGAAGATGTACACGATTGATACGTTTGAGGAAATAGATTATTGTTTAAGCAGGGGGTATAACCCCTTGCTATTTAATAATAATTTCGATATTGAGCCTAAAACAAGGTATGAATATTTAAAACGGATGTTCGGAGAGGGTCACGGACAGAGGGGAAATGAACGTTTCTTCCGGTATATGTGGGATATTAAGCCTCACTATTGTGAAGAATGTTTAAAGCCGTTAGCAGGGTACTCAGCCGTTTATATTTCGCATATTATAACGAGGGGATCGAACCCAATGATTGCGCATGATCCTCGTAACATAAACATACTTTGTTTCAATTGCCATAATCGTTGGGAGCACGCTAACACCCGGAAGGGAATGCGGATATATCAAAGTAATTTAGAAAAAATAAAAGTCCTCAAAAGGGACAGTTTAAAACTGCAAAAGAAATGAAATTAGTAAAATTTGAACTTGTATCTGGAAATGAAATTATGATTAACCCTAAATCTGTGGAATCAATAGTTAGATATACAGATGATTCGGTGTATATTAACACAGTAGGTGTAGATATGCCGTATAGGGTTAAAGGTTCAATTGAAGATGTCAATAAAGCACTAAGCGAAGGTAGCAAGATTGATTCAATAGCAGGACTTATGGTTATCGTCTTTATTGGAATTTACATATTATCAACATTAGCAAATTTATTATCGTAATGAACTTAAACAAAATCGAATTGATCGGGCGTGTTTGCGCTGATCCGCAAGTAAAAACCTTCGATAACGGAGGGAAAGTATGTAATCTTTCTATCGCAACGAACGAAAGGGCATATAAAACGAGTAACGGTATCGAAGTTCCGGAAAAAACAGACTTTCATAATGTAACATTCAAAGGTAAATTGGCTGAGATTTGCGGGCAGTATGTTACCAAGGGAATGGAGTTATACGTAGAAGGTAGTTTGCACTATCGAAAATATACCGACTCCAATAACGTTGAAAGAACTATTTCTGAGATCGTTGTTAGGTCTATGCAGATGGGAAGAAAAGCAGGTGAGGGAAACCAGCCGACAACCGGAGGCAACGGAAACCAACAGCCGACAACCGGAGGTTATAGCGGTCAACAGCAACCGCCTCAGCAGATGTTTACGCAAAATGATGATTTGCCGTTTTAATGTAGTTTATAAATTGGGGATGTATATTGCATCCCCTTTTTTGTTAAATACGTGTTAAAACTTAAAGTTTCGCTTGTAATATC

**CLONE3**

cttaaagtttcgcttgtaatatcaaattaagcccttatatttgcagtgtcaaaaggaaacaaagtactaacatttaaaaataaatattatggtaacaatgacatcaaaacaattttgtgagagaatgtatgcaatgtataaattacttggtgggagtgagtccggatgtgctcaatgttcaaacgatagattttcttgcggatatggaaaggagaatacggttttaaccaatgcacttat

gaaagcgtgcgataatcacaaagttccttataagatagaagcaaacgaatattgtatcaatttcgtagtagaatttaaataataatagcggtagaaataccgctttaaacttatagttatggaaaaaagaagattgtccggtcaatacaaaatagcgatgtgcaaaaatagaggaaacgatacatttgccggaactgttggaataagaacaggttttatgtatcagtgcggtgcgtatca

gtattttacttattgggagaatgacaataaaatatcggttactgaatcaagtacaggttttcgtgtaatgtctttggatgttgaaaagggagaaactcctaaaactgcgcatgataggatagttgataagttgaagggttttgatccatctttagaaaactggaatagtgctaaagagatgatgaagaaatataatattccctatcctcttaatgaatggataatagggctaaaagacat

aaaccatgaatgaagaggtagagaaagcaagatcggtaagtaacgaggttatttcggaaactatcagaaaatcgactgataatataaaggcaatggaggacgatttcagattagtaagaaagaagttgcggaaaattggcgatcgaataaaatttgagagaaagaaacttgatatatacaacgaagaaataaaaaggagggttaagtatggaatttggtaacttactgttatatagattg

gggttcaaccgtgaaatgttggaagataaactttcagaaatatccgctaaggagaaagagataagagtcctaaagaaagaagtttccggtataatggaacacatatcaaaattggaaagtgcgttaaatcatggagagcattattattgcggtgcttgctgctatcttgaaagtaaatgtaataagggaaaatataagtgtcttgaaaccggagaatacaagaaatatcactgtaaggcg

tgtgagaaatttagagatttaccattttaataactaattataaattaaatattatgattgattttaatcaaaaaagtatctctttagctaaagagtgtacagaacaacatgaaagaatgaaggcaaaaggtttttatgaatcagatgtttttgagtgtaaaaaatgggcgttgatagtgtctgagttctgcgaagctatggaggctgaaagaaaaggcagagttatagaaaacgatgtat

atgactttgctatgaataggaaatcaaaggtaggctttgagtcgtatttcaagaaatggataaaggatacagttagtgatgaactcgcagacgtgtttatccggtgtatggacgcaataggacattctattgataaaattgcgtgtcctaccgaaatttttgtttttcaaagtatggttagcgatcatttcaataggttattgtatcttgaaaaatctatttcatcaattgtttattatg

ccattcaatttgtgccgaaatctgtatttggcaaatcgtgcattaccgagtatactaacatgatggcaataaccattgcagccgcaaagctttataacatagacctatctaaagcaatagaggcaaagataagatataacgagttgagaggtaaaaaatatgggaaataatattaattcaattgatatggaagaaaaaattattgatttagcaagaagaagcgtttattatggtgatccg

gaaggttaccaagttgggggaggccattacaagacatccggcatgcagctttctgaatttttagaaaggaataaagttggtttcttggaggggaacgcaatgaaatatgtgtttaggcacgataagaagaacaaagaagaagatttgctaaaggctattcagtatatcaaattgattctaaaatacaaatatggtaaattcttagtaggtgatattctgttgagtgaagaagaatataga

aaactggatgagcttattgagaagcaaaatacgattgaatttgatactacttttatcagaaatgcgttaaaaaccgcatcaatcagagtaaacaaaatatcggtagaaaaagcaaacttgtatgttgcaaagctaagagaggttaaagccgaatatatcgaaaatttcgttttgtcggatatacaaaaacacaggcttttagatatgggtctacaatggataagaaatagcacgtattgc

cttaattttacggcaaaaaatggagatatgatatgtgttaagcctgggc

**Clone4**

GATATGTGTTAAGCCTGGGCAATATATTGTGTTGTTTGAAGAAGGTAAATATCAAGTGTTCTCAAAAGGAAGGTTTGAATTTCTTTTTCAACCGAAATACTAATAAAAATAAATAATGATAGGTCACGTTGCAAATATAGCAGCGTGACTTTATTTTTATATTATCTATAATAGTGTTATTTTTGCGCATATTGAAAGATTATATAATTTGTAGTACAATATACAGAATAGAAATTATAACTTAAAAATACGTCTTAAAATGGATAAAAAAATAGGTTCAATGAAAAGAGGGCAGGGAAGGCATAGCCGGACGGACGAACAGACTGAAAGAGACCGTTCCTTTGCCTCTGATTTGTTTTTGAAAGGTTATTCTTATAGAAGAATAGCGGAAGCGATTAACGAGCGAAATAAGGCGGATGAAGTGCCGTATACCGTGACTTATCAAACAGTGTATAATGATATTCAGTTTTGCCTGACTCAGTGGAAAAGAGAACAGTTCGATAATATAGATCAGTATATTACGCAGGAACTCCAATCTTTGGATAATGTAGCTCGTGAAGCGTGGGAAGAGTGGGAAAAGTCTAAGCGTCCCAAATGTAAGACAAAGTATATTTTAGGGAAGGCCAAGGAGGTGCAAAAGGAAACAACAACGGGTGATCCTTCTTTCTTGAATGTAGTTCTCAACGTGCAGCAAAGAAAAGCAAGGTTATTGGGATATGATTCTCCGCTATGTATAAACTTGGTAGGAGACAAAGAAAAAGAAAAGCCTAAATACGATTTTTCGGATGTTCCGGAGGACGTTTTAGAACAATTGGCAGATTCTTTGCAAAATACGGAGGGTAAAAAGTGAAAAAAGTAAATGAAATACCACCTATTGAGATTGTGAAGCATGTTGCGAGGAAGAAGTTTAAGAACTATGCTAAATTCATAGATGATAAAATAGTTCTGAGTCAGTTTCACAAAACTTACTACGAGATTCTCGATAGGTTTGCACATGGTAAGATCAAAAAATTGATTGTTACCGTTCCGCCTCAAACTGGAAAATCAGAGGGTAGTAGTAGAAAGCTACCTTCTTTCCTTTTGGGGCTTAACCCGTCTTTAAAGATATTGATCGGTTCTTATGCCGCATCACTCGCAGAGGGGTTTAATAAGGATGTACAAAGAATAATGGATACACCGGAGTATAAAAGCCTATTCCCCGACACCCGAATAATGGGAGAGGAAAAAAAATCGAGGTATCAAGCGTTTGCGAGAAACTCAAAAATGACGGAAACAATCGGAAAGGGTGGGTATGTTATATCAGTTGGACGTAATGGTAGTTTGACGGGTAAATCTGTTGATATAGCCATATTGGACGACTTGTATAAGGATCATATGGAGGCAAATTCTCCGATTATCCGGGAAGCTGCTTGGAAATGGTACACCACAGTTGTAACCACCCGTCTACACAATAACAGTCAACAACTTATTGTGTTTACGAGATGGCACAAGGACGATTTAATAGGGAGGATCGAAGATAAGGAGAATGTTATCACTGTTGAGAAGTGGGAAGATTTGGATAATATACCGGATGGCGCATGGGTTAAAATAAACTTTCCTGCTTTAAAGGTGGGAGAACCAACAGAGATTGACCCACGTTTACCGGGTGAAGCACTTTGGGAAGAAAAACATAGCGCTAAGAAATTGAACGCACAAAGAGAACTTGATAGAAATGAATTTGAATGTTTGAATCAAGGAAACCCGGGTAGTGCTGAGGGGACTCTATACGGTAACTTTAAAACGTATACCGATAAAAACGATTTTGGTGTGTTGGTCGGAAGGGGTAACTATACAGACTGTGCGGATACTGGTAGCGACTACCTTTGTTCAATTTGCTATGATAAATACCAGTCAAAAGAAGCGGTTTGGAATGAAAAGGAAAGGAGGTATAAGCATCTTATTTTCTGCCTTGTAACGGATATTGTTTATACTACCGCACCAATCGAAGAAACGCAGGTTAGTGTTCCTAACATGTTGAATATGAATAGTACAGATTACGCATACATAGAGAGTAATAACGGAGGGCGATCCTTCGCTGTAAACATCAGTCCAAGAACTAAGACTGAAATTAATTGGTTCTGTCAAAGGTTAAATAAAGAGGCTCGTATATTGTCGAACGCTGCAAACGTTACTCAATCTATTGTTATGCCGTACGGGTGGGAGTCACGTTTCCCGAAATTCCACGAACATATAACGAATTACCTTCGTGAATTTTCCGCTAACAAACATGATGATGCGGCAGACGTTTTAACTGGTATAGTCGAGAAAGAAGTTATTCCAACTATATATCAAAAAAGAAGAGGAATAAGGGTTATAAACTGATAAAGTAGGAAAATGTATCAGACTTTCAAGTTTATACGGTATATTTGCAAAGTAAAATCAATTGTTTAACTAAATTTTTATAATTATGTTGTATTGTAATTGTCC

**Clone5**

TTATGTTGTATTGTAATTGTCCTTTAGGCGAAGCAATTCCGGATATTCCCGCATTTAGCTGTCCAGACAATTTCGGGCAAGTTCAAAAACTTGCTTTTCAGAGACTCGAAAAAACGGCAGGAACTGCAAATACTATGACTGCCGAAAGTATCGTAAAGTTGGCTACATGGACTCCTTTACTGTCAGCAAAAGACGGTACTAAAGTAGTAGTTACGCCTTATATTTACGAGCCGACAGTAGAGGCGGGAGCTGCCCTTACTTATGGAGGTGGAAACGCAACTCCCGGAGGTATTGTAGAAATTTTGGGGTCGGAGTCGACACCGTTTACGGCTTCGTTCAAGAAGTTGCCGCAAACCATTATCAAAGCTATGAAAGCGTTGATGTGTGAAGCGGGTCAAATCGGTGTGTTCCTTATCAACGGTAACGGACAGATTGCTTGTGATAAGACGGGTAATAATTTGCACGGTTTCCCTGTTTGGTCGCTGTTTATCGGTGATAAGACTATCGGAGGTTTAGAAGCTCCGGATAGCAATGCTATTACGTGGAACTTCATCCCTAATTGGTCGGACAACTTCACTATCGTGAAACCTGAGTTTAACCCTCTGACTCAGTTAGTGCCTTCTACGGGTATAGGCGGATGATAGCTAAAAAAACGTATATTTCCCTCAGTTGTGAAGAACTGGGGGAAACTCGTTTATTCGATATTGAACACGCTGAGAGACTTTTGGGAATGGTTAATAATGGAGGGTGGCATATACCGGAGGACTCAGAATTTAAATTAAACGAAAATGGGAAAATCATTAGACGAAATAAGGGAGATATACAGACATCCGGAGGGGATCAGTCAAATAGCGAAAGCAAAGGAACACGAAGAAAGAATAGCGTTTCACACACGGGTGAGAACGAGTGATGATCGTAATAAGCCAGTAATTGACTTTCTTTCTAAGGTTAAGACGTGGATAGCGAAAGATAAATATGATATTTTCCTATCTATGTTCCATTTCCCGGTTAAAACAAATGGTGTTACTTCTGAGATATTCGACAAACTGAGCCGTGTTTTCGATGGTAGGAATCCGGTTTATAACTATCAGTTTAAATCATCTGAGGATCGGGATGACTGGGAGTATTACCGAAAGGATGTTTTAAAAGAACCTTCGGTTTGGAGTACGGACGGTTGGGATAATTTCAAGCATAGAATTAACTCTGTTTTGGTCGTTGATATGCCGGAGGTACAGGTAGGAGAAAAACCAGAGCCGTATTTTTTTTGGTTGCCTATTGCAAACGTACTTTCTTATCGCACATGTGGGAAAGACTGTAATTTGATGGCTTATATCATGTACGTAACGGACGAAAATAAGATCGTCTATATTGATGAAGAACGTTATGTAAGATTTGATAAAACGAGGGGAAACGACTTGATTTTAGAGGTAGACAATATGCACGATTTGGGCTATTGTCCGGCTCGTTTCTTTTGGTCTGACTCTATATCATTGAGTGAACCCGACATTAAAATAAGCCCTATAACGAGCGAACTCGACTCTTTCGACTGGTATCTTTATTATTCCACTGCAAAGAAGCATTTAGATTTATACGCGTCTTATCCGATTTATTCCGGTTATGAACGTGATTGTCACTATGAGTCACACGATGGCAAAGAACGGTGCGATGATGGTTTTTTAAAGAACGAAAAAAACGAGTGGATAACAGGTGAGGACGGAAAACCGATGGCGTGCCCGATTTGCTCAAGCAAGCGGTTGCGGGGTGCAGGCTCTTATGTTGAGATACCTATCCCGGACGAAATGCACAACGTCCCCGACTTGAAAAACCCGATCACTATGCTATCCGCTGATACCGGATCACTCGAATATAACGTAAACGAGGAAAAGAGGCTGAGAGAGGAACTTGTAAGATCGGTAACCGGTGGAGAAGGGGAATTAAACAGGTCTGAGGCTATTAACGAAAAGCAAGTTAAAGCGGGTTTTGAGTCCTTGACTACTAAACTAAACAGAATCAAACGAGGCTTCGAGGAAGCGCAAACATTCGTAGACTCTACTATCTGTTTACTCCGTTATGGTGATAGCTTTGTTTCTTGCAATATTAACTACGGGACTGAGTTCTATATCTATACACCGGAAGAGCTTTCAGAGCGTTATAAGATCATGAAGGAAACCGGAGCGTCCGAGGCGGAACTTGATGCACTGAGGCAACAGATCATCGAAACGGAGTATCGGAACGACCCTACACAGATGCAAAGGTTATTAATCCTTAACGAGATAGAGCCTTATTCACACTTAACGAGAGAAGAAGCGGTAAATCTGTATAAAGAAAACGTTATAAGTGAGGAAGATTTGCGAGTTAAATTAAACCTTACTACATTTGTGCGTAGATTTGAAAGGGAGAACATGAATATCATTGAGTTCGGTTCTGCACTTGAC

**Clone6**

GAGTTCGGTTCTGCACTTGACTATAAAAAGAAAATTGAAATAATTATTAATACTTTAAAAAAGTACGCAAATGGTTTACAGAACGGATCAGTTAGATCAACTGAATGAAAGTAATTACGTTTGCCCGAAGGATGAAGTTAAATTGTATCACGTTATTCAAGAAGTGAAAGAGTTTAACCCGAAAACAGGGCAAAGAATCAGCGTCCCGGTGTTGCAAAAATACAAGCGAAAGACTTTTGAACTTGATATTTTGCCGAGACTGCCAAGATTGGGTTATACATTGAGAGTTGTTTTCGACCCGGTTAAATATGAATCTACAATTTCAGAGGCAAGACGAGCCGCAGAACTGGCAGCGAGAGCCGAGGCAAAAATGAAGGCAGACGAAGAACTGAGAGAGCAAATTAGACGTGAAGAGGCTGCAAAACTTCGTGCGGAATTGAAGAAACAAAAAGAGAAAGGAGAAAAGTAATGTTAACAGTAGAATTGCTTAGGCAGAATAAAGCGTTATCGGAGCTATCGGATGAAGTTCTTAACGCTATTTCGGAGCTTTCAAAAAACGATGAAGCGCAGACGGTTGCGGCAAAGGTGAGAGAAGCCGAAAACAGTATTGCTTCTCAAATGAAAGAGGCTTTTGGTATTGAGGGTGTAACCGATCTCGATTTGAAAACCGCAATTGAGTTTGGCAAAACAAAACTTTCTAAATCTGATACCTCAGCTTTTGAAAAACAGATTAACGATCTGAAAGAAGAACTAAAAGCTGAGAAAGCTAAAAAGGGAGGAGACCGGGATACTGATAAAATCAATCAGCTTACTGCCGAACTAAACGACACCAAGCAAAAATTTGCTGAGTTGAACAACCAACTTTCAGAGAAGGAAAAGGAGTTTAACGGTAAGTTGAACGATTACAAGATCACTTCTTACATTTCAAGCGCTATGCAGGGGATGAAGTTTAAGAAAGATATTTCAGAGCCAGTTCTAAACGTTGTGAAGCAACAGGCGGTTAACTTGCTTAAAACTCAATTCTCACCCACTTTGCAGGGTGACGAAGGTTCTGAAAGTCTTATTTTCATGAAAGACGGTGTACCTTACAACAACCCTGCAAACAGTCTGAAACCGTTTACCGCATCAGAACTTCTGTCTCAACAGTTTGAACAGTTCGGTGTGCTTGACAAAGGTAGACAGGCAGGAGGTGCGGGTAGTTCCGGAGGCGGACAGGGTAACGGTAGCTTGCTTGATTTAAGCGGTTGCAAAACCAAAGTAGAGGCAAACAAGGTTGCGCAGGAGTATTTAGCTAAGAAAGGTTATACAAGCGAGTCGGAAGAGTATCAAACGGAGCTTGATAAAATTTGGGTTGAAAACAAGATCGCAGATTTGCCAACAGAATAACTAAAGAGGGGGTTAACCCCTCACAATATAAACTTTAAAACAATAGATTTATGTCGTTAATTGCTACAAGAACACAGGAGTTCAGATTAAAGAACCCTAACATTGACAAAAACATGGCTCGCATGACCGAATGGGGTGCGTATGACTTCTTTTTGTCTCAAACAAATGCGATGGACTCAATGCTTTCCGATGAAACTAAGCGTAGAGCGTTCGCATCTATGGGAAGCGATATTAAGATTCCCGTAATTGATTACGATAAAAACGTAACGGTGTCACACGCTCGCACATGCGTTATCGCAGATGCGGAGAACATTTCTCGTTTGATCGGTGTAACTTGGAAGACCTATGCTTTCGGTTTCACTATGACACCGAACATGTATTCAAACAACGAAATCGATTACCAACAGGACTGGAACAGAAAGCTACAAAAGCACATCCGTAAGTTCATGGATACCGTTGATAAGGACGCTATTGCGGCTTTGGAGGCAAACAAAACGCAAGTGTTCGGAAACTTGCTGTATTACACAAAAGAAGGTAATGATGTACAGGTGAAATTCACTCAGCGCAACGACATCCTCAGCGATTTGCACCCGATGTTCCGTGCAAACGACTATTCCGGTCAACTTCATATCATTGGCGACACTGGTGTAGACTCAATGTTGCGTAAACTGGAACAGCACGGTTTGTACAATGACGTTAACAAACAGTTGGAGTATGCAAACAAAGTGTTCCATTTCACCAACAACATGACTTTAGAGCCGGAAAACTTCGCTCAGATGTATGCTGTTGAATCGGGCAACGTTGGTTTGTTGACCCGTGTAGACCGTGCAGCCTACAACAACACTAAGTCGGGCACGCATGAATTTGGAAAAGTTGTTCTTCCTTATTTCGGTAAAGAGGTCGGAACACACTACTACGAAGAGGTGGGCGATCAGTCGGCTATCGCAGGCGCAGCAACTGCCGACATGACTTGTGACGTTAAACACTTCTACGGTTTCTCAGTAGATATTGCTTTCGTAGTAGCTTTTAACTCCGATACTTCAACAATCGCCAACCCGATTATGAAGATC

**Clone7**

CGCCAACCCGATTATGAAGATCGAAGTAAACAAAGAAAATTCGCAGTTTGGAGGTACTCCGGTATTTATTACCAATGCTGAACAGATCGGTGGAGGTTCTCCGGCTGGCGAATTATCGGTTAACCTTGCTAAAATCGGAGGTAGCCCGGTTGCTGAATCTGCTTTGAAAGTAGATTTGGATAAAGTCAAAGGTGCAGCGGTTTCGGCTACTGGTGGCGTAGTTGATGTTAAAGTCAATGCGCAGGCTGCAAATCTTAATGTTGAGGTGAAGAACTCTGATAGCGCACCTGTACCAACAAAAACTGTTGGCGGAGCGTAACGAGAAAGTAAACTAAGTATTAACAAAGGGAGGGGGACAAAATCCCTTCCCTTTTTTTATTTATAACCATGTACAGATTAAAGGATATACAAAAAGAACTTGCCACGCTCGTAGGATGGCGGCAGTCGTACGATAGAGACGCTAAGATAGACGAAAGTTTAACGGTGTCCGATAGTGGTGTTATGTTTCAAGACGTTCACCCGCTTGTGACGCTAAGAAACATTGAATCTATTATGCCACTTGATTACTATTTACGTTATCCGGAGTATCGGGATACCGACACTTATAAGCCGGGTGACAAGGTAGTTTACGGCAAGGACGTGTTAACGCTTCGTCCGGACGTATGGGAGGCAATAACAGAGAATGTTGGTGTAGAGCCTTCAGAGGGTGATAACTGGAAACGGTACAATCCACTAAGCGATTATTTGCGTGAATTGAACGAAAGAGCGATCACCAATACCGTTACTCGCTTCATCAATGAAAAGTTGATTGCAGGGGAAACAAAGACGCTTTTGGAGCGTACAAACTTCTTCGATGGTTCGGGGAAGATAAATAACGAGATTGACCCTACCGATAGTATTGTAGGATATGAAATATTGCCAGTCCGTTCTATGGGAGTAACAGCCAAGATCGAGAAGATAGGTTTGCAGTTTAACAAGCCGGGAAGGGTAAGACTTTACCTTATGCACACCTCACAGGTAGACCCGATTAAGACGTTTGATTTGAATTATACTAAAAATGGTTCTTATCAATGGTTTGATGTCGGTAACGATGTGTTACTCCCTTATATGTCTGAGGAAACCTCACCCGGTGGCTTGTGGTACTTGTGTTACGATCAAAAAGAATTGCCGTTGGGGATGTATGCTATAAACGTCTCTAAGGACTTTTCACGTGACCCGTGCGGTACTTGTAATATCGGAAGCGTGCAGGCGTGGAGAGAGCTAACAAAGTATATCAGAGTGTCACCGTATAGAGTTGACTCTACGCAGTCGGAGGATGGCGTAAAGATGTGGAATATAGAAATGAACATGTATACGTCTGCAATCTGCTACGGTTTAAATGTTCAATTGTCGGTAGGATGTGATATAACTGACTTTATCATTCAGTCTAAGTATGCCTTCACGCATGCCGTTTCTCTGCAAATGGCTTCTTATGTGCTGCGAGAGCTTGCATTAAATCCGAACGTCCGGCAAAATGCCAACCAATTGAATATCGACCGTGAAACACTATTGTACGAAGTTGACGGAAATTCACAAGGGCGTGCGCAGGGTATCGGATACGAACTAAAGAAGGCTTTTGAGGCTCTTTCTATTGATACAAAAGGGATGGATAGAATATGCCTTTCTTGCCGGAACAACGGGATAAGATTTAAAGCAACATGATAAGCGGTCTAATAGATAAGTTTAAAAAGGTAGGTGAGGAACTCGACACCGGAGAAATAGCAAAAAAGATTGTGCGTGACAATGATAATATACTTATTGACATGAACGCACAGGATCAGCTATACGCAAAGGGTGTTAACCGTTTGGGCGTTCGTATAGATGAATACCAACCCTACCGACCCTTAACTATAAAGGTCAAAATAGAAAAGAGGCAACCGTACGACCGGGTGACACTAAAAGACACAGGAGAGTTTTACGACTCTTTTTATGTTGAGACAGCAGAAGATCGGTTTTACATAAAAGCCTCAGATGAAAAAACTAATTGGCTTATCAAAAAATACGGTGCTGAGATTTTCGGGTTAACAAATGATTCACTTGCTGAGTTTATTAACGATTATGTGAAAGACGAAGCATATAACAGAATAAAGGAGATATTAAATGAACGATAGGGCTATAATTAGACCAAATGCGACACTTTTCGATAAAACGATAGCCGATGTACAGGTAAGCCTAACAAAATCGCTTAAATGGCTTAATTTCGCTTTCGGGAACGTGGTTAAATTGGTAGAGAGAAACGAGAGGGGGAAATTTGTTACCCCATCAGTGTATTTTAAGGGAAATGATTATTTGCGCTTAGAGCCGGACGATAAGCGGGGCAACGTTTGTTTTTTCTATATGCACGACTCACAAGATTATGAAGGGGGAGACTCTTTGTCTGGCTTTGGCGATCTGAGGGGG

**Clone8**

GGCTTTGGCGATCTGAGGGGGacggttagcattatcttttggttcgatacccgtaaaatcccgggagcagaatattacaacgtggagtttgtaaagtcagaaatactgagagccttaacgcacgaactttatctgccatccggtgatatacaggtgagaaagatattccacgacgccaacaacgtatacaaggagttttctatccaaaagacggagaatcaatactacgtttatccctatgcgtgtttgcggtttgagtgtgatattcattgcgaagaagggtgttattaaagggggagtttccccctttttgtgttaaatacatgttaaaacttaaagtttcgcttgcaatattaaataaagtccttatatttgcaatgtcaaacaacgaaagaccccacaatctaaccaagacgcaaaaagattgttgaaagattaaattcataagagtagaaaataagcaacggtatctacgaagggttaaatgaaggttcggtatccgattaaatgaagctataaagcctaaatctttcgaagtatgacaaattatgtagtaacaattaaaaatcaagattatgacagcaagtttatttattcagagaacagttgaaaaatttattatgatggaatttgttaagggaaacatggataccaaagaacaagttgatacaatgatagaagttatcaaaagaaagttagacttttcgcatgatgaagcatgtgattttataagaaaagcgatcggaataaacgaataactttaattattgacaggtggggataataccccacttcctaaaataaaagccatgaaagtagataagtatctaaagagccacaaggcaaacgaattttacgtgaaaaagtgtagaggttattatctcgttatggatagctatgataaaagtttggcgtctatggaggttacagaagaagaagctaaaaaggtagccgagcaacttaacaagattcgcaacgaaagattgaatctaaccgttaaataatagttaaatattaaatttaaacgtgcaatattaaataaagtccttatatttgcagtgtcaaaaggaaacaaattactaacaattaaaacccaaagttatgaaaagatattttgtaaacggaaaagagataagcgaacaaaaagcaaaagagattgaagctaataataaaaagtatatggaaagcaatgacctttctctttgggcgaaatgtgaatttataacagttattgtaaagtaaaacaagtgggggtaacaccccacataaaaaaaataaatatatgactacttacatttataaaggacaaaagataagccactccaaaatattatccctattgcgtagtgcaggcatttacggaggaaacaaactatcatattatgaagttttagttaaagctgccgaaaacggcaacgaaagagccgcatatattttgagagacttaaaagtgatataataaccgtgggaaaccacacaaattttaaaggtatgtttaacaaggaaagaattgagaatttagaaaagagagttaaggaactggaaagaatagaaggtatttcaaaactttcggaagatgtatctaaaaggttgatacatgctttaattgactctaacaacgaagtagtgaaaagagtttttgatgatgtgtgtgttcgttcggtatatggtacatctgtgtttcagccacctaaagccggagacattcacgcaagtaatgcggtgtttaacggtatacccgattctttaaagaaggtagaagaggataaggcagaagcacctaccattgcaagcgtgttggaaaaagcaagaaggaatacgattgcgatacaggagcttttaaaacgaacaggatgttcgaacgtgaacgaagtaataagcaagtttgaacttggggtttctttcaaaaagatgtatgatgaagaagcacgcaaaaggaaagagcttgcaatacaaagagatcgtttagaaagtgaaatgatgtgcaaaatacaggacttagaatcaagaagagaagagttgttaacagcaacagggtgtgcaaactttagcgacttgaaaaataagttcttatgtaaggatataaagagggatgaactaatagaacaaataaaagagcttgcacggcaaagagatcttttatatcgtgatttaaggaatcaaatagctaaactttctgataaaaaccaatctttgatgcaaagtgaaaaaagccttatttgcaaacttgcagacaagaacgaggaattaaagagagtggaagaactttcagacggtagatataaagaagtcgtttggattcgtggcgaactgaaaaatcaagaacaggcggtagaaaaactaaaagacgaaaacaaacagcttaaatatgctaattcgaaaatggcaaaaagaacggttgattctatttgttcagaatcggatattgcggtaggatattcaaatttgcaaaagagatgtaaggatttgg

**Clone9**

GCAAAAGAGATGTAAGGATTTGGaaagagataaaaaatcattgcttaattccgagagagaattacaaaaacaagtatttgacctttcgagagataaaaaatacttggagatggcaaacactgcactcctgcaagaaactcgtaaaattagagagtctttgaacgaaagaattaagaagctaagacagagacttaaaaaatcgtctatccgttacagagacttaaaagaaatcatttcgcacaacggtttgaaatcagtataacaataatagccgggatattatcccggcacaatattaaaagatatgttgaaggtagatataagaaataagatattaaaacccaaaataggtgagattataaccgtaataggagatatgtataccacggtagcggaaacaataccgtataaaggtgaggggtgtgaaaaatgtgcttttcatgattccggaaaggtagggggtgattgtggtgattttgtggcatgttctaaacttacccgtgaagatgatgttatgtttaaactaatagaaagaaagagaactaaggaggttgaaaatgaatagaatatctttgtatgatagagatagattcgtaccgaaagagggtgaagtgttttttgcagaagttccgggaaagggaatagaccggaaggtagaggcggtattattgaaagacaatagcggttgtaagaattgcgcattttttaaaggagaattaaaagacttgtgtatgcaaataaactgccttaatcgtggtaggcaattaatttttaagagggtgagaagatgaagagatttaaaagagtagaactgtatgacaccttcacaatggatcacccgataacaggtgaagttatcagagtccaagcaattgaaggcaacaatgtaatatcatgccgggaatgtttattcagacaaaaggggtttaaaaagatatgcccacttatgagatgtttagatatgtctacgggaaagagtcaagcgtataaacaagtaaagttatgaagaaattagatttgtcaattctaccgattgatttaaaggtaggtgaggaaatggaaatattaacgcccgaaggtgataaggttacagtaaggtgcgttgaggatgaaagggaaaatatgtgtgattattgcttcttcgggaaatcggtttctagaatttgttatagcgttaaatgcagcgtagatgagcgtaaagacagtgtaagtttcaaagaagtaaaaagggagagactcgagaaaggaaaattattatgaaagaagtgattgactttaaaatcggtgatgaatacaaggagggtgatatactaaagacgagagaggggatatatttgctcgtagaaaaaacggataacaaacattgcgttaaatgttgttcttcgtgttggtttcaaaattcacctttggacgtatgtgtacaaatagattgtaccgcaggaaatttttattttagaccgtttgaaacatacaaagaaggtgaggaatacaacgtaggtgatctattgaaaatacctaaaccgggagagccgggaaagttcatcctttcaatagtagtagaggatgatattgtagacgaatttaataatagttgcgaacgatgtgtattcaaagactgggtatatcctacggatgattgttgttcaagaaacagatgtgtagattgtcttagagatataaacttagacgatgtatattacaaaccattagcggaggtataagaaatgaagcaaaagaaagtgagagattttgaggtgttcaaagtagtacatccgatcacagcaaacgaagtaacaattcaagcaataccacgggataccatttcatgcaacggatgtgccttccgaaagggagatttagaaagcatgtgtaaagcatatctgtgcgttagtgaaagaacgttagattgtttggtgttcaagaaagtaaaggagaaattcaaatgaaatgttacagagttttaaaagttaaagtattaatttaaatgtgttgacttatgaaagaagaagttgttttaatgctctctgagctaagatcgcaaattgatgatacaattagtcgtgttgagaaagaaagtgcagcagaggacaaagaaatcatgtctacgttagacaactgcgaatacattatgttaggttcttgcattatcttcccacagcaatttatagactggtgtttatcccgtggctttttgtctaccgccactgaggaaaattctctttttggaggttcttatatgattcaaagatatgtgtcaagaacagaccttatccgtatgaatggaagcgatataaccgttcatccttcattgttgtacgcatacttacacagaggtgaaaatgaataatacaaaggactgggaggaatacctcaaagatgtgagtaactccgaaaaggtgggcgaatatatagccaatatgatagatcacgatgaaagggagaaactaaagatac

**Clone10**

GAAAGGGAGAAACTAAAGATACttttagatatttgcgagaaaagcaaggatagcagctacgatctgcctatcgactgcaaaatataccttcctttaggctctttgttgtttgaacatgaaatgcttgattttataacgtgggctgataagatgggatatattcggtgtgaagaagacaagattatcattatttcgtctatgattaaaaggcggtttatcgttggatcgcttaaagttatgccggagattgtggaagcgttcgttttatacagaaagcatgtaggttaggggtcttttagactccttttcttatttataaacatttcgtttttatccaccttcggagagttcgagactaacgttttaataatcaatatctttgcaaaattgctttttattcatacttttgtacaaactaatatttgaattatggagatttataattattttctttcttgcgtgctacttgtttcgtttgtagcggcattttgtgttaactttgcccgaaagacgggtgtaattgaacggatgtcagtgtttggtgattcttggttatctaaggtgttccggtggtatggtgatagatcactgattaacgagctaatcaactgcgatttctgcctatcgttttgggcgtgtgttatttgttcagtgatcgtgtcgatcggaacgctaagccctattttcatccttacaccgatctttgcaacacctatttgtagaattttaatttaatgattatggagattagaaattatgtatcaattatcccgcctttcgagatcgtgaaggcggttaagtttaacggtgatgttcacgaattagcgcagctattgccaagttttgaactcctttccgcaatggatggcgtaatgatggcacgaataaacggcagcgcttttcgggtgtttgataacgattttatcgttcttggagaaaaaattgcttactcagttgacgaagaaacgtttgccatattatacgagcaggcagataaggaggtgacgaatgaacacgattaaggtagggaatcacacggtaacggtatacgaaggcattgatgaaatgcctatcgtccgttatcagaagtttaaccgtcttatgctgattgagtcgggagtcggtagcactattgaggaactcgatacgcatttgcaacgtgctattgtctattgcaggacacagccggaacatacgtataacgagctaatgaatctaaggcagtgtttcaatatggcagcgaatggtgtacatcccggaatgatggcttttgccgccttcgttaaggcggtcgatggcgtggaatatccggttaacgcatccgattccgatctaaaggcgatatttgacagcctcagcgatgcaactattaacgaactttctgagccgtttcagaaggtcaaaaaaaaatagaggcggaagtatcggtatactttccacggatggcggacgatcctctgattaaagagtattacgatattaaactatctctgataaaagcaaagttagataagcttgtgaacaacgtagataacagtgaggcggtgaaggaaatagaagataagttgctaaccttcttcccgcctcgaatattctacgggactgattcggtagagataaagacggataaggagtttcaagaaatgtgcttggtaatcactcagaatatgcacataaatgcacgtgaaatgtcggtgtctgagttttacaccgctttcgagatgataaagagacaggcaaagagaagtaagaataaataaatttaaatcaaatggcgaacgaagtaaagggaataaagtatagcgatcttatacaaccggacagcagtataaaggacgctattacgcagttggagggactgcaaaagatatatgacgctatgttaaagcgtatcgaggaaggcgcaaaagggctgcaaaaacctatttcagaaggtggaggcgcaacggaggaagggcgcaaaaagatagacgcctacgaaaagcaagtgcgatccttggcgaacgctgagatacaattgaaattggcactgacagagacagcgcaggaaatcgcagtattgaagaaacagacagccgatcaaaactatctgaataagttgcaggcgaagttggctaatagtatggcaggaagctataacgctttgtcggcacaatacgagctaaacaaaataaagatgaataatctgtcgcaggcttatttggagaatacggaggcaggaaagaagcttgttaaagagactgcggagatttacgcagcgatggataaataccaaaagagcacgggaaagcacacgttaagcgttggtaactacaaacaggcgttcgatggtttaggcttttctatatcacaggtcgctcgtgaacttcc

**Clone11**

CACAGGTCGCTCGTGAACTTCCatccttggcgatcagcgcaaataccttcttccttgctatttccaataacattcctatggttatagacgaaatacagaagttgcgtgcggcaaacgaggcagcagcgaaagcaggggaggcacaggtaagtataaccgggaaactggttaaatctctgttctcgtttaataccgtgatggtgttgatattgaccgccttttctatttggggtaaggatataaccaactggataggtagcctattcaaaggtaaaacaacagtagatcagttgaagcgatctactaccgacttgaaagattccatgttagaggctggaaagagtgccgtaaacgagtctgtgaggttgaatatcctgtataaagcggctaccgattccacacgcagccaaaacgagcgtttgaaagctgttaaggagctaaagaaagagtatccggagtaccttaaaaacctatcagatgaagctattatgacgggaaacgcatcaaaggaatacaaggaacttgcaaaacacattctatcggtcgcaatggcacgtgcctacgaggaaaagatacaaaagaacgcaaaggaagttattgacctcgaagaaaaaaagaaccaagtattagaggaaggtcggaagacttaccaaaagcaacaaaaggagatcgaggaacttaaacgttcgtctaagggtatcggtgttggtgcggtggctttggaagcggctttgcaagggcaagcgtccgcatggaataccgccaaaaaggaggcaaagagctatgacgaacaaatagcagttatcaataagtcgagtgaggaacttgctaaaaaggtggttatccccgatcttcttgcaggggacaaaggaggtaagacgaaggaaaggacaaagaaggactttgatctacaagctgagtatgaaaatagccgtatagctcttattattgattcacgtttgaaagagcaggaagagcgtaaaaaggcaacggcagatgaactgaaaaagctaaaggatagcacaacggagaaacaaagagctacgcagttatatgctgataccgtatacaatatcgaggcaaaattgcgtagagacttggataaactgcaaaacgactggcgggtagaggacttgcaaatcacgcatgaccgattgagtgaacgcctaaaggctgttagacgtggcacggctgacgagctattaatacaagtgcagttactcgaaaacgaaagagcgcaggacgaattgcgtattaaacagtcaaccgatagcgaacaggtgaagaatgaacgtttgcttatcctgcaaaggtcgtatcagcttgcatctatccaactgcaaaaggatttcacggaaaatcaagacaaacgtataattgatcggtcggtgttccgacttaatcagcagcagcaggcggagagtgccgcctttaatatggtgcaacgttcggagaaagaacagagccgtttccggttgaaattagagcgtgaaaagtgggagcaaatattagagttaacaaggcagtacggagagcaaatcacgggatacaacgtaaagacggtagaggataccattaagggaatagacaatgcaattaagcgtgatacttccggatgggacagcaatcaaggcgtatttggaaatctgtttgatcttgttttcggtgacgcctttagcgcaaaagatggtaagtcaggcgcagagcgtgcagaacagtttaaagactccatattagaggcttcggagttcgccatagaaaacctaaagagtgttgcgcaggcaagggtagaggcggcagaagttgcggtacaggcagcagagaaagaagtttcagcccgacaaaaggttttggacgctgagatacaagcgagggcgaacggatacgccaacaacgtagcaaccgcacaaaaagagcttgattttgcacgcaaacagcaggaaaaagcactgagggataagaagaaggcgcagaagcagcaagaacgcatagatacacttatgcaggcaagttctttggtaacagcaaccgctaacctatggaaagatttaggtttggcagcgatcccggctattgcgttgatgtggggatcatttgcttttgctaagataaaagcctcacagctatctaaagcctcgcaggacacggaggaatacggtgacggtacggtagaaatgattgattacggaggttcgcacgcatccggaaacgatgtagatttaggtacgactaaggatggtaagcgtagacgggtagaacgtggtgaatacttcgcagtagtgaacaaacgttcatctcagaagtataagaaactcgttccggacttgattaattcgctaaataaaggtacttttgaacagaaatacttaaacgcctattccggtagtgatgaagtaacgaatataatgcaaggttcaacggttgatctgtctaaggtcgaaaaagatctgaaatcaatcaaagagcaggggcgtgttaagtacatcacaggtgcagacggcacgataattgaagtaaggggaaatattaaacg

**Clone12**

GTAAGGGGAAATATTAAACGaataattaaatcataatgaacgttaaagatttgcggtttaaattggggggtgtagaaatacatccccactattcagagctaaaacggaagtttggcaaagagaatcaacaggagtttttcagagagtcgatagaggggagtttaacgctgatcggggcggactaccttcttgttaaaaatgcgagtattgaggatattttgtacttgcaaatagagcaaaaggataaagggcagctatcaacgcagtatcaagtaatatttgagggctatttcagtaagacagattgtgagatagacagcgataaccgtacgtgcaaagtgaagataagcccacgggacgaatacaccgacataatgaagggtattgagaacaaatacgatcttatcaagcttgcacccgcattgtctcaaataggggtatccaagcgtccgattgtgcaagtttacattgcgggtgcatctacaatatcgaactaccttgcaggcactcactacgaaactgaggttttcaacgttgtaacggataacaaggagctaacggataagaatttctttgccttcttcgctgcatacaacgaaatagaggtaaaggcagtgccttatcagttctttaacggaaagtactacggaacgaatggaacgtacactaaattggatggcaatttctcaataaaatggactctaagcgaaggtttaaatattggtttccttcacttggaaaataaagagggaactatattgtaccgatccgataagatcaattggagcgataaaagctactactacatagatgtttctgaaataacattcacaagaatagtagatgatccgacacttcctcaaaagtttggcggaaacactgttcttttgcagaagctatttcaaagaatgttgcttaaccttccggagttggacggtaaacctaccgggaaactatcatcagaggacgtttaccctaccaatagcaactacatgtatgccgcaccattaaaagggaactacttttatacgtctacgaaggttcagaacgagccaacagagtatggtgtaaatgatgaaggcaattattttaccgataacttcgttccgtctgtggcgggtactggaaagttgtatccggtatgccgttcacgatgggggaatatgtcgatttggttcgagtttgatttatcctatgcgccattggaggaaagagcgagaaaggagtatgttttaaaggactcgttcgccatacaggacgctattagggcgcttattaagcaaattgatcccactttgacgcacgaagctacggaagaatatagtaagtttttgtatgctgccaataaccctatttccggtgcaccttttaaggtgttcatcacacagaaaagcaacatcctaaagggtgagtatgaccgtccggcaaagaaggcggaaacaaccctcagcgatataatgaagatgttgcgtgacacgatgaaactatattggtttatagatggcgataagtttaggatagaacatatttcttacttcataaatggcggaagttataccggtagcgggacggtcggcatagacttaacaaagcttagatatgcaaaatcgggtcagttaatgacgtggaaaactaacacggtcaaatatgataaaaccgatctgccttcacggtttgaattttcttggatggacgatacaacaaatacgtttgcgggtttccctattgatgtgaaatcaaactacgtgcaagagggaaagaaggaagaaataagggtgtctaacttttcgtccgatgtagattatatgctactatcaccgggtgacttttcacaggatggttttgcgctgctgggagctacgcagataggcggtaaatggaaactaccgtttgttacattcaatttggtagacaagaacaataagaagtacaccgtaaacccccaaaacggctacatgtcgttcttgcacctcgttaaatactacatgcacgatatgccagcctcagagattgaacacggaggcgatcagacgataagagtgagaggaataaagcggagtatgacgcaagatttatctttcacatacgacaccacaccaaaccccgtgcaactgataacaacggatataggcaacgggaaaccgataactatgactgaggatctaacaactcgccaaataaccgtatctttatcttacacccccttataatagggggtgttttcttttaaattgctatctttgtgcctataatcaattttttaatcaaaatggaagtacataacaactttagtcctttggcgtttagaaagaaagaatctaaagccacatacgaaaaatggtacgctttcgggaagaactacgctattcctgcaagcgcaaacacgctaactcctttccagtttacagagttgaacataccagtctttgatcccg

**Clone13**

CATACCAGTCTTTGATCCCGacacgatcgaagtagaagcggttaacgaggaaacgggagagtcgacaaaaacgggtgtatatgttagcttcgatgtaatgcccgaacatggcggtgtattgtacgtgtcacccggcaagaactcgtttagggaggctttgccacagggaacgtatagagcacgtttttcaatcggtgatgaagtatatatttcgactcctttttgcgttatacccggcatagaaacgagtagcaaatatctattgattgaatattggaacgatgaaaagattgcctatccgggtggatttattacaacgggtgcgaacaatgacttccggtatcagatgtatgttcctgcaacgatctgcaaacctaaatacgagtttgaagaagagctaaccaaacgtgccggatacaagtttttggaactgcaaacgtctacgaaggtgtacgcctttacattcgttgcaccggagtttatttgtgacgctatgcgactgattcgcctatctgactatatccgaatttcgcacgatggcgaatattacaacgctctcaacttcgagtttgatgttgattggcaggaacaattatatttggctgctgttgactgccagtttgagacggactcaatcatacaaaaactcccttctttcaatagacgagataaagcgtctttttataatgccctattagcgaacattgatacacctataatgttctctcccgataccgtagggctgtattacaaagagtatcgggaaacagagccagtagtcaagggtaaattgatacgggagttatcccctattgacttgatagatgaaaatacaactattgccgttgatttgggtacaggtgaggcgagaaagtttaacttatatcgaatgttgcaggactacatttctaaaacccatgaagatgcaacagactttttgttacaccttcgtggaggcgcaacgttcggtgagggcataactggttctgccgcttctatcaacgcagtaggagatgcggaggttcaagggctaaacgcacgtgtaaccaaagttaaatcgattgattcggaagattatgtaactgttaataaaacagccttcaccgtaaacaaacaaggtgatacggctttaaatgcgcttaatgcgaggggagattcccacttgcagcaagatgtgtataccggaaacaataccggaaagatcaccaaagaaggacaattgcagtacctatcagccgttatacaggagtttatcacatcacccacctttgttcccggttttttgggtgagggctttaaaatatgggttgagaatggcaattggcatatagaatgtgacaatttgacagtaagacagactatgaatatatttgaactacttatccaaaagataaggagcgttaacggtgcattggtcgtgtcccaatcaaacggcaaattgtcagctgttgaagaagtaggaacgcaatataagctaaccacaggagaggaatttcccacttttcaagagggcgatttagtcaggtgtcagacgtttgcaggctatcaaggtgcgggacttacctttaactttactcagtttgcaaaatatgactattccggtggtgcttttgatagcagcttgattgatgttacacccgactctattagctttaacttgaatgatactggtaattccggttttgcattctataaattttcagagtcaagccctacaccaattgaaataccttcatttaccttgactttggagggaggctatcctggtatgatggcttttgctgccggacttgattcaaatgacagtccggtagagggtgtaggcgtgttgctgcaaaacggtgataatgttattccggctattaaagcagcgcaaggcatacacaactttgctataacaataactggtgattccggtcacggtgatggtaaggttacagtaaagcaaaagaaagcggcaggaagcgcaccaaacaatagcttagttaaattctattgggtggaagttaaagcggttgacgggacttctttctttgcagataaagcggagttcaacggtgttgttccggctgtcggtgatgaagtcgttcagatgggaaatacgaagaaccccgaacggcaggcgttaatttatatcacagcgcaggaaagcggacacccgtacatagagatattgaacggagttaaaactaaatcgttgtccggtacgaatagaacacgtcttggcgatttaagcaacatacaagattctgcatttccggaaggacaacagccatccggtagcggcttgtattgcgataacgctttccttcgtggtatattcttgttgagaaacggaaagtcagttgaggatgaagtaaaccaagcgaagcaagatgcagccaacgcagcaacagatgcggagagagcacaacagacagcgcaggaggcgaaagataggcttaataaatgggctgacgatggttttatatc

**Clone14**

GGGCTGACGATGGTTTTATATCtcctactgaaaagcctgctttgattgatgaaggaaagcgcatacaggcagagtttttgcagataaaaaataacgctgacaaatacggtgtatccgttactgaatataccaaggcttatgaagattatttaaatgaacttagataccattccgcccaacagccggaagatattgcggtgcgtccggaacttgcaaagacgcaaacgatatactacgatcggagaaacggagcgttgaatgctattgcgaacgctgcaaagagctacgtagatgaagctgacaaaaagctaaaggagtatttagatacggagatcacagcaataccgggtaagattgaactcgctgtgcgtagtttgaaagtggctgatgttaacttattgaaaggtgcatataaggagctgaaaaatgctgactatatcgtggggtattacttttatgatgtaccagttatagacggaaaggagtacactttaaccgtatgttacacacttggaagcagcaatacagaaatggcagtatactctaatggtggcacaaatcgtattgctacccttgtgacaaaaggagataaagttgtagaaagttataagattactatggcaggttataaaccgacttcagctatgtatttctttcaattcccaaacggtacatacggctcaaaagtacattgggctgttttgactgatggcaatttaggtgtaaccagttggataccgtctgcaagcgagaaaaatgtggggttaaagaatctttgttcttttaagcgtattactgatgcagggtttacctatgctagaaactacgaagatgatggaagttggtatattaatcctgaacttttgcatagtgaaaccagattagctaataaggatatgtttggtctaacttatgacccgaataagcaatattatctattcatagacgctgctaaaaggactgatacagaaacggacaaaagatgtacttattttataattaaatatactgatggctcagaggatatagtttgtgaggtttattcagataaggtaacacataatttcatcactacacaaaagcctatttctaaaatagtaggtagttatagttatggttatggtacaactattagagtcggtttatacgaaacgaacttccccgtttcttggagtccagcgccggaagatcagttgtatcaatctgttaagtacaccgatacgcagatacttgccgttgacggaaaaatagaactatctgtaaagactaaggtagaaaatttgggaataggagctaacaatttgtatagctacacgagttcaccgcttaatcaaatgagttctgagtcaatgactataacaaggcttataagtgaacacggatttcatttggtgggtgcaaaaggcggtcagtcctttgtaagaatacctaatgttataccgcctatgcccggaaaatacactatttccggatggattaaaggtagccaaaataccccagttggttttagattggatatttgtgactctgaatcgtatattgttaggtctaatgcacaaaatacatggagttattttaagcacactttcgatgttacgaacaatacagaagagcaaagcggtatatataattttgttgatatacaagatatatcatgggccaatatatggattaaagactttaaagtcgaatacggtgaaatagcaaccgcatggagtccaaacgaggctgattcggtgtatttttcaaaagaatatacaacgtcacaaataaacattgttgaaggtaagataacatccaccgttgaaaagattaatgcggttgacgggaaagttaccggacttgcttcacgtgtagaccaaaccgaaaagagtataacgtcagttgttggggatattagtgttattaatagtaccaccaataggcatatatcaaagctaatagatttaagaggatgggacaataataagtttttcccgttggttataaatattccggtttaccacaaaacaagggttgaaataagtagacctcttgatgcgggatacggaaaaccttcatacggtacacatgatggcggtttttctatgaacttaacgtttgagatgtccggttcgggttggggttcgttgccagcagtaactaatatctttgactatactaaagcatggacttctgcgggtgcaaagatagttgttgatttgggacaaataactgagacttctacttgtgtgatgggtatccgtggggggtctaaatatgatgtaaccgtttacgacacaacagaccccaatgtgataaacgtttatcaaacagattatcacggttcgtacggtacatctttccccgttcgcaccgatggtacagagccattccggacgtatggttactactctgagataaaacagacacaagaaagtctctcagcaacggttgcaaaggtagacgatc

**Clone15**

CGGTTGCAAAGGTAGACGATCaaggcaggcggttaagtgcggctgagttaacattatctgccgatcacgcaaaattatcggtggtagaaacgactgcaaacaatgccaattcacttgccggaactgccaacaacaaagcggaagcggcaggcggtcgagttaccgccacacagaacggtttggtcgagacaggaataaacatcacatcccgcaaaatcgtgctaaagtctgataacgtcgttttccaaaacaacgcaggacagcagacatccgctatcaatgcgaacggaaaacttactgcaaacacaatcgaggttggagaagttgttgccggaggttttgcggctcagagaatcactaccgggaacttgactgtgacggatggggcggtgattgcaggaatgactatttcgggaggtgttcttactgggaagaatatacatatcacagacggggcgaagatcggtaactttacgatcgaaagcggtattctttctgctaatggtatggtagcaggaatgcaaatgcgcttatctaataaccttatggagataagtagctcaggacttcgcatagatcacaattccggtggttacgctatcaatgttacaggcaacggacgttgctatatgcaaggtgtagagtcctaccgtaaattcggtgtatatgagtttgctggtgcgacacaatggaaagcccccggagtacattttgcgtgtactatttcctcagcggcagcagtacgccaaaagtggggaaacccctctatcggtgtgagtgccgtaaaacactctaccggaagatatacagttcggtttaccggagttcattcacagggctttattcctatggtgatggctctaaatgctaccaagtgggtaaacgcttgtgtagaggatattgttagtacgacacagttcaccgtcaaacttatggacgttaatactggaatggtagatagcgactttgctatatacatttgcggttttgtttaatagttaatatttgcggtaagttggtttataccttcttaccgcttacctttgtaccaaacattaatcaattaatataaaattatggaaaagaaaagtttagattttgatttaaagtcagtagtttacacgaaagaaacaaaagtgatggactaccatttcgagacggaaaacggcaagtacgtaggtcaattaacaacggtatcgacagagccggacaagtacaacattacccactgtacggctgatgtgtcagagaaacaaatggtagaaatgcctggaacttccggtagtccaattctgcaagaacaatacgttccggtcggatcgcttgccatccgtgacggtcgctttgaggcaaaccagtttcctctatctactaaaacatccgtctatgtgaacgactttcaaaatttcatctttgcgttaaccgcacctaaaacagtagaataatgaatgtcacacaagaacagttaaggttaatgcttgtatcggtgataagtccgatacttgcgtttcttacccctacgagcggttttataaccgcacttgtgttcatgttcggctttaacattgtttgcggtctgcgtgccgatggggttaatttgtcggcaaatggtgttcgtaggttcactatgctgaaattcatctcagccgtgcaggaacttattttgtacatccttgtgataaccgttatcttttcgtctgtggctaagatgggggatcacgacgcagccgttctatcggcaaagacgattacatacgtctttatgtacgtatatctgtcgaacggttttaagaacctttgtatcagctacccggataacaaatctttccgactgatatactacattgtccggttcgagtttaagaggctgatgggagaacgtgccgcaaagatagtcgaggaacacgaagaaaagattgagattgaaactaagtaattaacacgggaggtttaacgcctccctttaaactttatcaaaatgaagtatttcacattaaaagagctaacacgctcaacaacagcaacggcaaaaggcattgataatacgccaacaccggaagttgaaaagaatttgaccttattagtagaaaacgtattagaccctctacgggagatttacggcaaaccgatcacggttaattcgggttatcggtgtcctgagttaaataaagccgtcggaggttctgcaacgtctgaccattgttttgatgaaaaaacagaaattttaactacaaaggggtggagatcatataataatatatccaaagatgatatattatatacatacaatgtagataatgatataatagagaaaaaaactataaataggatcgttataagagaatatgacggggatatgatcggatttaaaaatagcagcgttgacctacttgttacggacgaacatagaatgcttataagttaccaaaggcataagtataaaagaaaggg

**Clone16**

GGCATAAGTATAAAAGAAAGGGtagtagaaaaatatccgaaaagggggctttgtatttcgatagtttgaaaactaataatgacaaatttcattttgagctcgctaaagatgtattaggtaaaagacgaattttcaaatgttcttcttataaaaatggggtgagtagtgatataagagttatgaaaatatgccttgcatttatttgcgatggtttttataacaagaaagcaataggttttagattcaaaaaagaaagaaagataaaagaactcgaatatatattgaataatgttgggtgggaatatacgaagcgagtagataaatgcggagttactaatttctatctaagaaaaaaatactatgatattattttaaatatcgtagggaaggataagaatatacctattgacgtattggaatacaatagcgaactactaaaagaacttttattttactattgttcgtatgacggtcgcttcgacaaaagaaataataatacgggtttcgcaatcggtacaaccaataaacataatgttgatgtgcttcaatccatggcttgtatgtgcggaataagatcaaatatcttatattacaaagaaagagagtataacataaaggggcaaacaggtatagctaaaccttattatattctttcgggttctttgaatactcaatcagatttacaatcatgtggatcttttgtagagaaatacaatggagtcgtatggtgtgttagcaatgataatgaaacggttatcgtgcgaagaaatggtaaggttagcatacaaggtaactgtaaaggttttgcggctgatattacaggagggagcaaggaagagaatgaacggttgtttaatatcattaaacacaactttcatttctcccagttgattgacgagaaaaatttcagttgggttcatgtgtcgtacaacccgaataacttaaaaaaccaaatactaaaactatgaaacgacaattatttgcgtttttagcgacttttgtgctttgccttggcattgtgtcgctattactgataaacgctgatttacggaagaaaaagtctattgcagaaagaaatgttagcgtcctcacaactcagaacgttgcgtatcggacgaaaagcggtcaaagtgccatgaaagtagaggaattgaatctgactttaaagcagtaccggaacaccatacaagggaaggataacactataaaggagctaaagcagtctattaaggacttgaaaagtcacacaagcgttcaaacatcaactgagacgcattttagcacgccagtacgggatagtgttgttcttcgtgatagtttggttatcgacacaatgaaatgcgtaaatatgcgctctaaatggcttgatatatccggctgcatagatagcaacggcacgtttgccggaacaaccgtttcccgtgacagcttagagatacttaatatagagcatagaaagcggtttttgtggtttcgactaaagaaggtgaagtatagggagtttatcgtaacgagcaaaaacccctacactgatataacaggttttaacgtaactacgataataaagtgataattccatgttaaaacagttaatgcacgttaaagtatttgccactgagaaatatatccgtatatttgcagcgtagaagttattactaacgtcactaacagcggttattgattttcataaagttatgtttgtttaattattaatattcacattctcaaactgtcggtatgcgaatatagacagtttttaattcgaacattttcactaactatatatattgggttttgtcataattacatttttccccctccgcttgtgaaagtagaggggttttttattgccttatccgaacacgcctaaaaagttaaattcgtgttaaacattaaacttatgctttgatatttaaaatatctccttaactttgcaacatcaaaaggaaacgaattactaacaataaaacttagagttatggaagaaaaggaatttatttattgcttgaccggagagattaacgtattaggcactgtcaaggctaaaacaataaaaagtgctatgaaacttgtagcggctattcagagaggtgctatattgaatgatccggaaaggaaatcaatcttttggagcgtttcacgtgctgatctcccgtttaaacttggtcgtattgtatacacaatatgctatcccgatgggtctattcgttcacatgtatgctaacaataaaaatttagagttatggaattagtaaaattcagagaggcaaagaggataatggaagaaaaagcttttttgcaaaaaacgcttgaaaggtttcagtccggctatcttagtagaacagatttatatttcagttcgggaagtagcgtaacattttcagaaggggatggcgaattttatgaaggtttgtgcaaagacttagaaaa

**Clone17**

GTCTATAAGAGAATCTATTGaaaagcgtattggctatcttgaatctaaatttgataaactatgatacgatcatttagtaagtcgggttcaacatctatgctgacagataaggaaaaagcgtttaaccgctactgcctaactaacaaggaagtttcatataacttaatgcgtatagaaatggcagttgttcaaatgtcgcattacggcaaccgttcatcagatgtcacgctaacaaccgatagttctgaggttttggatgcaatttatacagtcctaacaaatgaagggtttaaatactctttcaatttacctaataaagtattaaccataagtattttttaatttaaaatttaatcaaaatgaaagaagaagtaaaattgttcagagcgttaatcattgtttttgtgttacttgtgttcaccttcgtgttaacttcgtgcggtgatgatagtgacaatgtgtatcaaacagaatattctattgatgttccggaatggcagacggtttatgttaatggtgaggttacaacgtctatgtctccatatgtttgggaacatgtggacttatcagacaaatgtgttagagtattctcagcagggcatgttagttatcacaaggttacaaaggtgtcacacgatgatttaggctttaccgtttattcaatagaaagtagcaatagcgaaaggtttgcatacaataagaataaaggtatattgcaatattggtgcacaagaaatggcattgaaaccgttgttgtttatcgtgaattaaagtaagtttcattttaccctcacccggtggaggttaaccgggttattaagtatgaaagtaattgttagatttaaagacgaagaagaaattaccaattttgaagctaaatacggtttagaggtagagaaccataacggacaaagatatatcattgagttcgataatatgggaaatttggtggttaatagtccgaacggtgcgttattggtaaaaccacaatgcagtaataaaatatcaattagacccgaataacatgaaagagataaacgaaactcaattacagctatcgactgagggaaaaagacttcccgatatgataaagcaggcgaacggcatacacgaacttgttaagcagaaactttctgagtataactcaatagagtataccgatgataatataaaggtggcaaaagccgatagagccactttaaacaaggcgaaaaagggacttaacgacagccgtatagaacttgaaaaggcttggatgaaaccgttcaacgaactaaaggatgttgttaacgaaacttgtaagctgatcggtgaggcttcttcgcgcatagatagcaagataaaggaaacggaggaaaaggagaagcaaaagaaactggatcaaataagggagtatttcgaggaacacaatgaaaatcttatcttgtttgattttgctttccgtccggattggcttaataagaccaaagcactttcagttgtgaaaatggagatagacgaattatttaaaacggttgacgatgatcttaacagactgaaagagcattttgccggagaggcgttttatattccggttatcgacaaatatacgtctacactcgattataacaagtcgttcgattatggaaatcacctaaaagaagctgcaatacaagccgcaaacagacagtttgaacagagggtgacagataacacgcctcagcaacaaaagcccgaaattaagctccaaaacgagccaaagacgaacgaagaagaagtttatatacgaggttttaaagtccatgtaacgagaaagcaggcttttgcgcttgctgagtttatgaatagccacaatataaagtttgaaagtatatcaatatagacggtagcccaattgggctaccttttttgttttgtttgcaattgttgatctattgttgaaacctaaggtttcacttaaactttcaaataatgtgcttatatttgcagtgtcgaaagaaacaaagtagtaacaattaaaaattagaattatgaatgaaattaaatgcgttaaatgcggatgtgtgattaatacagatgcaggatactatgctgcttttgatggtggcttttgcagaaagtgttggattaaacaatcagacaaatttaagagtgaacagttggtgaaagcactcgaaaagagattcaaaggtaatattaacaataatttattttaaaatggaacagtatttagacttactaaaagagactttaaattatggtgaaaagagatcagatcgaacaggaacgggaactattagcttattcggtttacaacgatcttatgatttgcgtgacggtttcccgcttgtcacaactaagaaggtattcacgaagggaattatatatgagcttctttggatgttaaaaggagacaccaatataaaatacctaaatgaaagtggtgttcatatttgggacgaatgggcaaagcc

**Clone18**

ATTTGGGACGAATGGGCAAAGCCttccggtgatcttggacgtatatacggtaaacaatggcgtgactggcgtataaatagcaagttaagagtagatcaaattgattcagttatagatatgattaagttcaacccgaagtcaagaaggttaattgttagtgcttggaatgttggagaaatacacatgatggcacttcctccgtgtcactgcttttttcagttctatgtgtctgagtccggttatttggatttgaaactgtatcaaagaagtgcagacctatttttaggcgttcctttcaacattgcgtcttattctatcttgctgtctatggtagcgcaggtttgcggcttaaagcctcgtagattcattcacactatcggggacggacatatatatttgaatcacgttgaacaggtgaaagaacaattgagtagagagccgttcgcccttcccaaattggaattaaacccgaatgttcgtaatatattcgattttaagtatgaagatattaagatagtaaattataactgccatccggctataaagggagaggttgcggtatgaatgaaaaagaattttacaggtttttagcctataataaattggtatattttgaaagataccttcacatggaatctgtatattatctgaataacttgctaaagaaaaccgttaattcgtatttgagagattgtatattgaacgctataaatcataaattagcgggattataatttaaaagggatgtgcaacgctttgccatccctttttagtttctatatatcacataccgaaactatcgttgctctatgaaacaaatctaacaatatgtagtaacaagtatgaaagtgatgcaaaggtaggcttttgtcagtttctaatgatcaaaacggatcgttttacatttcattaacaataaaattaaagaatttctttgcgtatttaaagtttatccttaactttgcaacatcaaaaaagaagtagtaacattaaaaacgaataatatgcagattaaaaaagatcaaatttacaaattgcttgtgcaggtttgcaagaatgaaggcattccattctcctacgaaaaacttgttttgtttttgaataagtacattgatgaagatgaagaagattcagtatttggatatacaatatctgatattgatatttcaatcgctaaacatatatcggttgatatttgcggaatgcttacattaagcaatattatcagtcaattaacttgtatcggtgggggagattgcccgaattgcggaggtttactcagattgatagaatcttatcccaaatttagcaaacagtattgcgatcgtgattgtgagccggagagagaggaagaaaatgtatatgaatgtttaacatgtggaaaggaggttgttttatgaatattgaaaacacaatgatccgtatcaatgatgcgattataagcgcacgtatgaacggcaaaaagattacgaaaaaggatattgcggctttgctgtggaaggactcaaagcaaagaacgcaggcggtaaacatgtctgccttgtgtaatcacaaaacccaaactataaaaatagagtgggtgaaagagatatgcgaggctaccggagtcgatgcgaatttcttgtttaatattaaccctaaaaaataaaagttatgattaaaaatttgcctaacattcaaaacgaaatgaatgttcaaaagtcgagatataacaagtttggcggatacaattaccgttcgtgtgaggatattttgcaagaagcgaaaagggtgtgcgaaaaatacggatgttatgttatggtgactgactctatcgaatttatagaagggcgtttttacgtgaaggcaaccgcaaagattgttgaaactgaaaccgggtctattgaaacgtgttcggcttttgcacgtgaagaagacagcaaaaaggggatggactcggcacagctaaccggggcgacatccagttatgcacgaaaatacgccttatgtggactttttgcgatagatgatagcatagatactgattctacgaacggagagccggaagcgaaagaaaaacggcaaaagacagcctcaaaacaagctatcaaccaaagtaatactggaagcaactcaaattatttgggtgtgcttcttgaagaaataaaaaaagcaacaacttataaacatttgggcgatattcacaagaataacgctaattaccatcaaaatagtgagttcatgaacgctttagttgtccgtaaggcggaacttgaaaaggcggaagcggaagcaaagaaagtataaataattcgggggatgcgttcccccaataaaaataaaagcaatatgaaagaactaacattactccccaaattggttaatgctgatgtaacgtatatcagcgaaacacatgaatatttttcaagcgattttagaaagctgagagggataacgggttttatcaatg

**Clone19**

GGGATAACGGGTTTTATCAATGatcaattatttcccggcaaacttgacaatataccggataatattttgagatcggcaactgagagagggaaagcggttcatgatgaagttgagaggatcgacaaagaaggtattgaaccggaaacggtctacggagagaactatttgaatttaaaagccggaagcggtttaattcatatcgcatctgagtatattctaactgataacgagtttatcgcctcaccgaccgataaagtatatttgggtagctctgataaatcagtagttttaggtgatgttaaaactacctataaacttgatttgctttatctgtcttggcagctatcaatatacgcttaccttttcgagagacaaaacccaaatttgaaagtagagggacttatcgcaatttggctaagaggtgacaaggataaggacggaattttctctgttgaacgcataccggacagcgaaatagaattattccttaattgctgtaagaatggagttcgatatgtagacaatgcaagcaaagatagctacgtagcaaaattggaatcattacccgcaaaagtagcacatattgaagaaggcgtttacgaacttcttgaaatgcaaaagaagatagacgaacatttaggcaagttcaaagaacagttgttaggtctgatgtctgaggcgaaagctgataatataaaaggggaacttatttcagtcacaaggaagaaagcgtatagccgtgaatcacttgattctaaagcactgaaagaacaatatccggaaatatacgatcagttcgttaaaacatcaaatgttaaagagtcaattcaattaaaagcgttataattatggttataaatgaagatttagcaaaagaaatcggtttagatgattcagtagtatattcagttatgtttcttatactttgtactgatacatataaggataagtttaagggatgtagggtaaagaaagaacctaataccgtttttattacgatttctaaacttagagaaattattccttttatgtctaagagcaaattatataattctgtaaacagattattaaagcgtgggtatataaaagaggctaattatagactacccggcactaatacaactaagtgttatacaatcggagaactaaaaacagcaaataaatgaaacaatttcatgaagaaaacccaccgattgaggaagattttaacccgtgggatgaaaaagaaagatcagtataattttaataaaactaacgaacaggcaaaggataattatgtacttgaaaacgatttagattatgaagaatagacttatgaaaaggattgttggttattcgcattatgtggataaacttgtatatgcaacggtagacgggggaagaaggaaaataatgaaattaaagccaaggaaaaagaagttttatacttccctcgaatattacagggctatacaaaagatgtttgaaggatgataacaatatctgaaagttttgcgaacgagatcggtttaaatacaacccgatgttaccaacttgttaatgtgtcacaacatttcgagtaacctataatacacccaccaacgttttttagttgtgtgggtgttttttgtacagcactattctgtgcagtctgtctatacctaattatcagatatttgcaaaacaaagaatgttaaatcataaaaaatagatgttcttccattgcagtatataataataaacgctatatttgcagagtaaaatttaaaacagtatgtatatgaaagtagaaaacttagttaagattaagagttatgccgatttaaagggagttacagtaccttggatatggaggcttattaagaggggaaaattagagtatattcaaattgatggtgcatgcttcattgagttaacggatgaagaactgaaaaagtatgcggagtacaaagaacggataagttcatttttgaatagcaaatagtattaatcattaaaattttagaaaatgaaagaattagtttttaaaggagaatcaaatcaagttttaactaacagcttattggtagctgaaaagttcgggaaagagcaccaacatgtattgcgtgatgtgagaaatttaataaatgggggtgtgtccaaaattggtgatacccccgcatttgcggaagcaacttacattcatccgcaaaacggacaagaatacccgatgtttataatgaatagagacggttttacattgttggcgatgggctttaccggagaaaaggcgcttcaatttaaattggagtacattaacgcctttaataaaatggaggaaactattaagaacggaggttttaacgtgcctaaatcgttccgtgaagcattattgcttgcagccgaacagcaagaagttatagagaatcagcaaaagcagatcgaagaaaaaaatgcaaaaatcgaagctgataagccgaaagttctgttcagtgaagcagtc

**Clone20**

GTTCTGTTCAGTGAAGCAGTCtccgcctcgaacaaatctatcttagtgcgtgaacttgcaaaacttatcacacaaaacggttatcagatcggggaaaagcagttatacgagcgattgagaaaagccggatacctttgcagcgttggagaatcacgcaatcaacctacacagacatacatgaatatcggtttgtttgagattaagaagcgtgttattatggacggtgatgaagcaaaggtttacaatacaactgtcgttacgccaaaaggagtacattatttcattaataagtttttagggaagggaatgaaatgacacattgttttgacgataaagtagcaacaaagttaggagttgaagcggcatgcgtattgcacaacttcgctttttggataaacaagaatattgccgataaccacaattattttgagggcagatattggacttataacacaagggaagcgttatctaaactattcccgtatatgagtcaatctaagatatatagagtgataggaaagttggaggaagaaggctatttgttgaaggggaattttaataaatcgggtatagatagaacaacgtggtacgcattaacagataagtgtataaaattcctttttgagtgcggatatacgcttataggctattctgagccgattttgcaaaattgcaaaatgcaagttgcagaaatgaacaatgcaagttgcagaaatgaacaaacaataccagatagtatatatacagatagtaatactaaatctactaacgtagattatagtatagccacgcgcgaagaatctgttttgttcccggttgaaaagaaacctttagcctcagagatatttggctttactgcaaaagccttagatgtgactaagaaagtgatagagcgaacagatagttttttcgatcagctaacattcccgttcgagtcggaggaatttaaaaaagccttttatgtgctaatgactcaaccaaagtggcgggtaaagactaagactctaacagctatgcaagcaaacctaaacgagattgcgcaatttgaagaaggttttgctatgctattgataaatcagagcatatctaagggatgggcttcactggtatacgagtcaacgctaaaacagtatatgcaatggctacgggaaaagacgggagtctccggaaatacacagcctgcaaacaatactaaatcgtattttcagagtgacgaacagcgcaggatgtatcagtcttatttaacggaggattttacatagcattttaaggcttaaatttcaattttaatcactaagacaataaaagtatcatgtatttggagaaaatcgaaaattcgggcggaaaattagcaaaatacgaaggttgcggattgtttatagagaagaaccgaaaattttatgaaagtggcaacttcggacagctatcaaaagtagatcaaaagatattccgtgattctactttgcttttggtgtccgaatgtacagacgaaagaaaaagaatagataatttttctaaggttcttaacggagtatgtttagagactggtttaaaaatgccggatgtccgggacgcaggtagtatattttatgccgtttgcgatgtgatagatatgtatttctctgatctgtcgttcaatgaaattcgtttagcgtggaggttacttgctgtcggggaactcgacccgtttttgccaaaagacagatatggtagtccggataaaaatcattatggctctctttcggttgattacatttcaaaagtcctaaaggcgtataagaaaagaaaggttgaaacgatggaacgagtttctcagattatgccggacgaaaagccaaagccgacacccgaacaggaaaagatgtttttgaatttgcaggcatacaattttattctcgcccttttgaagtataaatattcgggtcgtttccgaatagagcgtgacaggataataaacgagtctacatttgcatacatggaaagattggggtatgatatgtcggtagtaccgacgttggcggacaagaaagaagctttgtttcaatttcaaggtagacccgtaaatagctttgcgcaaattttcgaaaaagagtgtatttcgagatttgggatagaccacgaagcagtttattttcgtgcggtactgatagccaagaaaagaaagttattccagtattgggatgaaatgttagctttctcaaatgaaggtgatagatcagaagataatatttggaagttgtactactacattcaataaaaccaaaagttatgaatagaagaaaagtaaaaaagaacggttatcggataaggcttacaaagccttccgataaattcgtttatgtctctgactcgttaacatacgaaaggagaaaaaaggagggaaagagatgttatactctgtattgcaaatatgcgtctattaactatttgt

**b. Secondary Subclones of clones 5 and 14.** These sequences were synthesised by Geneart (Germany) and ligated directly into pUC19 as EcoRI-BamHI fragments.

**PhiED1clone5/I**

GAATTCTTATGTTGTATTGTAATTGTCCTTTAGGCGAAGCAATTCCGGATATTCCCGCATTTAGCTGTCCAGACAATTTCGGGCAAGTTCAAAAACTTGCTTTTCAGAGACTCGAAAAAACGGCAGGAACTGCAAATACTATGACTGCCGAAAGTATCGTAAAGTTGGCTACATGGACTCCTTTACTGTCAGCAAAAGACGGTACTAAAGTAGTAGTTACGCCTTATATTTACGAGCCGACAGTAGAGGCGGGAGCTGCCCTTACTGGATCC

**PhiED1clone5/II**

GAATTCAGGCGGGAGCTGCCCTTACTTATGGAGGTGGAAACGCAACTCCCGGAGGTATTGTAGAAATTTTGGGGTCGGAGTCGACACCGTTTACGGCTTCGTTCAAGAAGTTGCCGCAAACCATTATCAAAGCTATGAAAGCGTTGATGTGTGAAGCGGGTCAAATCGGTGTGTTCCTTATCAACGGTAACGGACAGATTGCTTGTGATAAGACGGGTAATAATTTGCACGGTTTCCCTGTTTGGTCGCTGTTTATCGGTGATAAGGGATCC

**PhiED1clone5/III**

GAATTCCGCTGTTTATCGGTGATAAGACTATCGGAGGTTTAGAAGCTCCGGATAGCAATGCTATTACGTGGAACTTCATCCCTAATTGGTCGGACAACTTCACTATCGTGAAACCTGAGTTTAACCCTCTGACTCAGTTAGTGCCTTCTACGGGTATAGGCGGATGATAGCTAAAAAAACGTATATTTCCCTCAGTTGTGAAGAACTGGGGGAAACTCGTTTATTCGATATTGAACACGCTGAGAGACTTTTGGGAATGGTTAATAGGATCC

**PhiED1clone5/IV**

GAATTCACTTTTGGGAATGGTTAATAATGGAGGGTGGCATATACCGGAGGACTCAGAATTTAAATTAAACGAAAATGGGAAAATCATTAGACGAAATAAGGGAGATATACAGACATCCGGAGGGGATCAGTCAAATAGCGAAAGCAAAGGAACACGAAGAAAGAATAGCGTTTCACACACGGGTGAGAACGAGTGATGATCGTAATAAGCCAGTAATTGACTTTCTTTCTAAGGTTAAGACGTGGATAGCGAAAGATAAATATGATGGATCC

**PhiED1clone5/V**

GAATTCTAGCGAAAGATAAATATGATATTTTCCTATCTATGTTCCATTTCCCGGTTAAAACAAATGGTGTTACTTCTGAGATATTCGACAAACTGAGCCGTGTTTTCGATGGTAGGAATCCGGTTTATAACTATCAGTTTAAATCATCTGAGGATCGGGATGACTGGGAGTATTACCGAAAGGATGTTTTAAAAGAACCTTCGGTTTGGAGTACGGACGGTTGGGATAATTTCAAGCATAGAATTAACTCTGTTTTGGTCGTTGATGGATCC

**PhiED1clone5/VI**

GAATTCACTCTGTTTTGGTCGTTGATATGCCGGAGGTACAGGTAGGAGAAAAACCAGAGCCGTATTTTTTTTGGTTGCCTATTGCAAACGTACTTTCTTATCGCACATGTGGGAAAGACTGTAATTTGATGGCTTATATCATGTACGTAACGGACGAAAATAAGATCGTCTATATTGATGAAGAACGTTATGTAAGATTTGATAAAACGAGGGGAAACGACTTGATTTTAGAGGTAGACAATATGCACGATTTGGGCTATTGTCCGGGATCC

**PhiED1clone5/VII**

GAATTCACGATTTGGGCTATTGTCCGGCTCGTTTCTTTTGGTCTGACTCTATATCATTGAGTGAACCCGACATTAAAATAAGCCCTATAACGAGCGAACTCGACTCTTTCGACTGGTATCTTTATTATTCCACTGCAAAGAAGCATTTAGATTTATACGCGTCTTATCCGATTTATTCCGGTTATGAACGTGATTGTCACTATGAGTCACACGATGGCAAAGAACGGTGCGATGATGGTTTTTTAAAGAACGAAAAAAACGAGTGGGGATCC

**PhiED1clone5/VIII**

GAATTCAGAACGAAAAAAACGAGTGGATAACAGGTGAGGACGGAAAACCGATGGCGTGCCCGATTTGCTCAAGCAAGCGGTTGCGGGGTGCAGGCTCTTATGTTGAGATACCTATCCCGGACGAAATGCACAACGTCCCCGACTTGAAAAACCCGATCACTATGCTATCCGCTGATACCGGATCACTCGAATATAACGTAAACGAGGAAAAGAGGCTGAGAGAGGAACTTGTAAGATCGGTAACCGGTGGAGAAGGGGAATTAAACGGATCC

**PhiED1clone5/IX**

GAATTCGTGGAGAAGGGGAATTAAACAGGTCTGAGGCTATTAACGAAAAGCAAGTTAAAGCGGGTTTTGAGTCCTTGACTACTAAACTAAACAGAATCAAACGAGGCTTCGAGGAAGCGCAAACATTCGTAGACTCTACTATCTGTTTACTCCGTTATGGTGATAGCTTTGTTTCTTGCAATATTAACTACGGGACTGAGTTCTATATCTATACACCGGAAGAGCTTTCAGAGCGTTATAAGATCATGAAGGAAACCGGAGCGTCCGGATCC

**PhiED1clone5/X**

GAATTCTGAAGGAAACCGGAGCGTCCGAGGCGGAACTTGATGCACTGAGGCAACAGATCATCGAAACGGAGTATCGGAACGACCCTACACAGATGCAAAGGTTATTAATCCTTAACGAGATAGAGCCTTATTCACACTTAACGAGAGAAGAAGCGGTAAATCTGTATAAAGAAAACGTTATAAGTGAGGAAGATTTGCGAGTTAAATTAAACCTTACTACATTTGTGCGTAGATTTGAAAGGGAGAACATGAATATCATTGAGTTCGGTTCTGCACTTGACGGATCC

**PhiED1clone14/I**

GAATTCGGGCTGACGATGGTTTTATATCTCCTACTGAAAAGCCTGCTTTGATTGATGAAGGAAAGCGCATACAGGCAGAGTTTTTGCAGATAAAAAATAACGCTGACAAATACGGTGTATCCGTTACTGAATATACCAAGGCTTATGAAGATTATTTAAATGAACTTAGATACCATTCCGCCCAACAGCCGGAAGATATTGCGGTGCGTCCGGAACTTGCAAAGACGCAAACGATATACTACGATCGGAGAAACGGAGCGTTGAATGGATCC

**PhiED1clone14/II**

GAATTCGGAGAAACGGAGCGTTGAATGCTATTGCGAACGCTGCAAAGAGCTACGTAGATGAAGCTGACAAAAAGCTAAAGGAGTATTTAGATACGGAGATCACAGCAATACCGGGTAAGATTGAACTCGCTGTGCGTAGTTTGAAAGTGGCTGATGTTAACTTATTGAAAGGTGCATATAAGGAGCTGAAAAATGCTGACTATATCGTGGGGTATTACTTTTATGATGTACCAGTTATAGACGGAAAGGAGTACACTTTAACCGTAGGATCC

**PhiED1clone14/III**

GAATTCAGGAGTACACTTTAACCGTATGTTACACACTTGGAAGCAGCAATACAGAAATGGCAGTATACTCTAATGGTGGCACAAATCGTATTGCTACCCTTGTGACAAAAGGAGATAAAGTTGTAGAAAGTTATAAGATTACTATGGCAGGTTATAAACCGACTTCAGCTATGTATTTCTTTCAATTCCCAAACGGTACATACGGCTCAAAAGTACATTGGGCTGTTTTGACTGATGGCAATTTAGGTGTAACCAGTTGGATACCGGGATCC

**PhiED1clone14/IV**

GAATTCGTGTAACCAGTTGGATACCGTCTGCAAGCGAGAAAAATGTGGGGTTAAAGAATCTTTGTTCTTTTAAGCGTATTACTGATGCAGGGTTTACCTATGCTAGAAACTACGAAGATGATGGAAGTTGGTATATTAATCCTGAACTTTTGCATAGTGAAACCAGATTAGCTAATAAGGATATGTTTGGTCTAACTTATGACCCGAATAAGCAATATTATCTATTCATAGACGCTGCTAAAAGGACTGATACAGAAACGGACAAAGGATCC

**PhiED1clone14/V**

GAATTCCTGATACAGAAACGGACAAAAGATGTACTTATTTTATAATTAAATATACTGATGGCTCAGAGGATATAGTTTGTGAGGTTTATTCAGATAAGGTAACACATAATTTCATCACTACACAAAAGCCTATTTCTAAAATAGTAGGTAGTTATAGTTATGGTTATGGTACAACTATTAGAGTCGGTTTATACGAAACGAACTTCCCCGTTTCTTGGAGTCCAGCGCCGGAAGATCAGTTGTATCAATCTGTTAAGTACACCGATGGATCC

**PhiED1clone14/VI**

GAATTCAATCTGTTAAGTACACCGATACGCAGATACTTGCCGTTGACGGAAAAATAGAACTATCTGTAAAGACTAAGGTAGAAAATTTGGGAATAGGAGCTAACAATTTGTATAGCTACACGAGTTCACCGCTTAATCAAATGAGTTCTGAGTCAATGACTATAACAAGGCTTATAAGTGAACACGGATTTCATTTGGTGGGTGCAAAAGGCGGTCAGTCCTTTGTAAGAATACCTAATGTTATACCGCCTATGCCCGGAAAATACGGATCC

**PhiED1clone14/VII**

GAATTCCGCCTATGCCCGGAAAATACACTATTTCCGGATGGATTAAAGGTAGCCAAAATACCCCAGTTGGTTTTAGATTGGATATTTGTGACTCTGAATCGTATATTGTTAGGTCTAATGCACAAAATACATGGAGTTATTTTAAGCACACTTTCGATGTTACGAACAATACAGAAGAGCAAAGCGGTATATATAATTTTGTTGATATACAAGATATATCATGGGCCAATATATGGATTAAAGACTTTAAAGTCGAATACGGTGAAGGATCC

**PhiED1clone14/VIII**

GAATTCTTAAAGTCGAATACGGTGAAATAGCAACCGCATGGAGTCCAAACGAGGCTGATTCGGTGTATTTTTCAAAAGAATATACAACGTCACAAATAAACATTGTTGAAGGTAAGATAACATCCACCGTTGAAAAGATTAATGCGGTTGACGGGAAAGTTACCGGACTTGCTTCACGTGTAGACCAAACCGAAAAGAGTATAACGTCAGTTGTTGGGGATATTAGTGTTATTAATAGTACCACCAATAGGCATATATCAAAGCTAGGATCC

**PhiED1clone14/IX**

GAATTCATAGGCATATATCAAAGCTAATAGATTTAAGAGGATGGGACAATAATAAGTTTTTCCCGTTGGTTATAAATATTCCGGTTTACCACAAAACAAGGGTTGAAATAAGTAGACCTCTTGATGCGGGATACGGAAAACCTTCATACGGTACACATGATGGCGGTTTTTCTATGAACTTAACGTTTGAGATGTCCGGTTCGGGTTGGGGTTCGTTGCCAGCAGTAACTAATATCTTTGACTATACTAAAGCATGGACTTCTGCGGGATCC

**PhiED1clone14/X**

GAATTCCTAAAGCATGGACTTCTGCGGGTGCAAAGATAGTTGTTGATTTGGGACAAATAACTGAGACTTCTACTTGTGTGATGGGTATCCGTGGGGGGTCTAAATATGATGTAACCGTTTACGACACAACAGACCCCAATGTGATAAACGTTTATCAAACAGATTATCACGGTTCGTACGGTACATCTTTCCCCGTTCGCACCGATGGTACAGAGCCATTCCGGACGTATGGTTACTACTCTGAGATAAAACAGACACAAGAAAGTCTCTCAGCAACGGTTGCAAAGGTAGACGATCGGATCC

**c. Final subclones of PhiED1clones 5V and 5IX and PhiED1clone 14IX.** These sequences were synthesised as oligonucleotides (Sigma), annealed as duplexes with sticky ends and ligated into pUC19 as EcoRI-BamHI fragments. The subclones 14/I/A-F and 14/IV/A-F were not required for the current analysis but their sequences are included for a complete description of the library.

**Clone 5/V subcloned.**

....|....| ....|....| ....|....| ....|....| ....|....|

10 20 30 40 50

**5/V/A**  ~~~~~~TAGC GAAAGATAAA TATGATATTT TCCTATCTAT GTTCCATTTC

**5/V/B**  ~~~~~~~~~~ ~~~~~~~~~~ ~~~~~~~~~~ ~~~~~~~~~~ ~~~~~~TTTC

**5/V/C**  ~~~~~~~~~~ ~~~~~~~~~~ ~~~~~~~~~~ ~~~~~~~~~~ ~~~~~~~~~~

**5/V/D**  ~~~~~~~~~~ ~~~~~~~~~~ ~~~~~~~~~~ ~~~~~~~~~~ ~~~~~~~~~~

**5/V/E**  ~~~~~~~~~~ ~~~~~~~~~~ ~~~~~~~~~~ ~~~~~~~~~~ ~~~~~~~~~~

**5/V/F**  ~~~~~~~~~~ ~~~~~~~~~~ ~~~~~~~~~~ ~~~~~~~~~~ ~~~~~~~~~~

....|....| ....|....| ....|....| ....|....| ....|....|

60 70 80 90 100

**5/V/A**  CCGGTTAAAA CAAATG.... .......... .......... ..........

**5/V/B**  CCGGTTAAAA CAAATGGTGT TACTTCTGAG ATATTCGACA AACTGAGCCG

**5/V/C**  ~~~~~~~~~~ ~~~~~~~~~~ ~~~~~~~~~~ ~~~~~~GACA AACTGAGCCG

**5/V/D**  ~~~~~~~~~~ ~~~~~~~~~~ ~~~~~~~~~~ ~~~~~~~~~~ ~~~~~~~~~~

**5/V/E**  ~~~~~~~~~~ ~~~~~~~~~~ ~~~~~~~~~~ ~~~~~~~~~~ ~~~~~~~~~~

**5/V/F**  ~~~~~~~~~~ ~~~~~~~~~~ ~~~~~~~~~~ ~~~~~~~~~~ ~~~~~~~~~~

....|....| ....|....| ....|....| ....|....| ....|....|

110 120 130 140 150

**5/V/A**  .......... .......... .......... .......... ..........

**5/V/B**  TGTTTT.... .......... .......... .......... ..........

**5/V/C**  TGTTTTCGAT GGTAGGAATC CGGTTTATAA CTATCAGTTT AAATCA....

**5/V/D**  ~~~~~~~~~~ ~~~~~~~~~~ ~~~~~~ATAA CTATCAGTTT AAATCATCTG

**5/V/E**  ~~~~~~~~~~ ~~~~~~~~~~ ~~~~~~~~~~ ~~~~~~~~~~ ~~~~~~~~~~

**5/V/F**  ~~~~~~~~~~ ~~~~~~~~~~ ~~~~~~~~~~ ~~~~~~~~~~ ~~~~~~~~~~

....|....| ....|....| ....|....| ....|....| ....|....|

160 170 180 190 200

**5/V/A**  .......... .......... .......... .......... ..........

**5/V/B**  .......... .......... .......... .......... ..........

**5/V/C**  .......... .......... .......... .......... ..........

**5/V/D**  AGGATCGGGA TGACTGGGAG TATTACCGAA AGGATG.... ..........

**5/V/E**  ~~~~~~~~~~ ~~~~~~GGAG TATTACCGAA AGGATGTTTT AAAAGAACCT

**5/V/F**  ~~~~~~~~~~ ~~~~~~~~~~ ~~~~~~~~~~ ~~~~~~~~~~ ~~~~~~~~~~

....|....| ....|....| ....|....| ....|....| ....|....|

210 220 230 240 250

**5/V/A**  .......... .......... .......... .......... ..........

**5/V/B**  .......... .......... .......... .......... ..........

**5/V/C**  .......... .......... .......... .......... ..........

**5/V/D**  .......... .......... .......... .......... ..........

**5/V/E**  TCGGTTTGGA GTACGGACGG TTGGGA.... .......... ..........

**5/V/F**  ~~~~~~TGGA GTACGGACGG TTGGGATAAT TTCAAGCATA GAATTAACTC

....|....| ....|....| ..

260 270

**5/V/A**  .......... .......... ..

**5/V/B**  .......... .......... ..

**5/V/C**  .......... .......... ..

**5/V/D**  .......... .......... ..

**5/V/E**  .......... .......... ..

**5/V/F**  TGTTTTGGTC GTTGAT.... ..

**Clone 5/IX subcloned.**

....|....| ....|....| ....|....| ....|....| ....|....|

10 20 30 40 50

**5/IX/A**  GTGGAGAAGG GGAATTAAAC AGGTCTGAGG CTATTAACGA AAAGCAAGTT

**5/IX/B**  ~~~~~~~~~~ ~~~~~~~~~~ ~~~~~~~~~~ ~~~~~~~~~~ AAAGCAAGTT

**5/IX/C**  ~~~~~~~~~~ ~~~~~~~~~~ ~~~~~~~~~~ ~~~~~~~~~~ ~~~~~~~~~~

**5/IX/D**  ~~~~~~~~~~ ~~~~~~~~~~ ~~~~~~~~~~ ~~~~~~~~~~ ~~~~~~~~~~

**5/IX/E**  ~~~~~~~~~~ ~~~~~~~~~~ ~~~~~~~~~~ ~~~~~~~~~~ ~~~~~~~~~~

**5/IX/F**  ~~~~~~~~~~ ~~~~~~~~~~ ~~~~~~~~~~ ~~~~~~~~~~ ~~~~~~~~~~

120 Common to clone 5*/*XIII

....|....| ....|....| ....|....| ....|....| ....|....|

60 70 80 90 100

**5/IX/A**  AAAGCGGGTT .......... .......... .......... ..........

**5/IX/B**  AAAGCGGGTT TTGAGTCCTT GACTACTAAA CTAAACAGAA TCAAACGAGG

**5/IX/C**  ~~~~~~~~~~ ~~~~~~~~~~ ~~~~~~~~~~ CTAAACAGAA TCAAACGAGG

**5/IX/D**  ~~~~~~~~~~ ~~~~~~~~~~ ~~~~~~~~~~ ~~~~~~~~~~ ~~~~~~~~~~

**5/IX/E**  ~~~~~~~~~~ ~~~~~~~~~~ ~~~~~~~~~~ ~~~~~~~~~~ ~~~~~~~~~~

**5/IX/F**  ~~~~~~~~~~ ~~~~~~~~~~ ~~~~~~~~~~ ~~~~~~~~~~ ~~~~~~~~~~

....|....| ....|....| ....|....| ....|....| ....|....|

110 120 130 140 150

**5/IX/A**  .......... .......... .......... .......... ..........

**5/IX/B**  .......... .......... .......... .......... ..........

**5/IX/C**  CTTCGAGGAA GCGCAAACAT TCGTAGACTC TACTATCTGT ..........

**5/IX/D**  ~~~~~~~~~~ ~~~~~~~~~~ TCGTAGACTC TACTATCTGT TTACTCCGTT

**5/IX/E**  ~~~~~~~~~~ ~~~~~~~~~~ ~~~~~~~~~~ ~~~~~~~~~~ ~~~~~~~~~~

**5/IX/F**  ~~~~~~~~~~ ~~~~~~~~~~ ~~~~~~~~~~ ~~~~~~~~~~ ~~~~~~~~~~

....|....| ....|....| ....|....| ....|....| ....|....|

160 170 180 190 200

**5/IX/A**  .......... .......... .......... .......... ..........

**5/IX/B**  .......... .......... .......... .......... ..........

**5/IX/C**  .......... .......... .......... .......... ..........

**5/IX/D**  ATGGTGATAG CTTTGTTTCT TGCAATATTA .......... ..........

**5/IX/E**  ~~~~~~~~~~ CTTTGTTTCT TGCAATATTA ACTACGGGAC TGAGTTCTAT

**5/IX/F**  ~~~~~~~~~~ ~~~~~~~~~~ ~~~~~~~~~~ ~~~~~~~~~~ ~~~~~~~~~~

....|....| ....|....| ....|....| ....|....| ....|....|

210 220 230 240 250

**5/IX/A**  .......... .......... .......... .......... ..........

**5/IX/B**  .......... .......... .......... .......... ..........

**5/IX/C**  .......... .......... .......... .......... ..........

**5/IX/D**  .......... .......... .......... .......... ..........

**5/IX/E**  ATCTATACAC CGGAAGAGCT .......... .......... ..........

**5/IX/F**  ATCTATACAC CGGAAGAGCT TTCAGAGCGT TATAAGATCA TGAAGGAAAC

....|....|

260

**5/IX/A**  ..........

**5/IX/B**  ..........

**5/IX/C**  ..........

**5/IX/D**  ..........

**5/IX/E**  ..........

**5/IX/F**  CGGAGCGTCC 240260 common to clone 5/X

**Clone 14/I subcloned**

....|....| ....|....| ....|....| ....|....| ....|....|

10 20 30 40 50

**14/I/A**  GGGCTGACGA TGGTTTTATA TCTCCTACTG AAAAGCCTGC TTTGATTGAT

**14/I/B**  ~~~~~~~~~~ ~~~~~~~~~~ ~~~~~~~~~~ ~~~~~~~~~~ TTTGATTGAT

**14/I/C**  ~~~~~~~~~~ ~~~~~~~~~~ ~~~~~~~~~~ ~~~~~~~~~~ ~~~~~~~~~~

**14/I/D**  ~~~~~~~~~~ ~~~~~~~~~~ ~~~~~~~~~~ ~~~~~~~~~~ ~~~~~~~~~~

**14/I/E**  ~~~~~~~~~~ ~~~~~~~~~~ ~~~~~~~~~~ ~~~~~~~~~~ ~~~~~~~~~~

**14/I/F**  ~~~~~~~~~~ ~~~~~~~~~~ ~~~~~~~~~~ ~~~~~~~~~~ ~~~~~~~~~~

120 Common to clone 13

....|....| ....|....| ....|....| ....|....| ....|....|

60 70 80 90 100

**14/I/A**  GAAGGAAAGC .......... .......... .......... ..........

**14/I/B**  GAAGGAAAGC GCATACAGGC AGAGTTTTTG CAGATAAAAA ATAACGCTG.

**14/I/C**  ~~~~~~~~~~ ~~~~~~~~~~ ~~~~~~~~~~ CAGATAAAAA ATAACGCTGA

**14/I/D**  ~~~~~~~~~~ ~~~~~~~~~~ ~~~~~~~~~~ ~~~~~~~~~~ ~~~~~~~~~~

**14/I/E**  ~~~~~~~~~~ ~~~~~~~~~~ ~~~~~~~~~~ ~~~~~~~~~~ ~~~~~~~~~~

**14/I/F**  ~~~~~~~~~~ ~~~~~~~~~~ ~~~~~~~~~~ ~~~~~~~~~~ ~~~~~~~~~~

....|....| ....|....| ....|....| ....|....| ....|....|

110 120 130 140 150

**14/I/A**  .......... .......... .......... .......... ..........

**14/I/B**  .......... .......... .......... .......... ..........

**14/I/C**  CAAATACGGT GTATCCGTTA CTGAATATAC CAAGGCTTAT ..........

**14/I/D**  ~~~~~~~~~~ ~~~~~~~~~~ CTGAATATAC CAAGGCTTAT GAAGATTATT

**14/I/E**  ~~~~~~~~~~ ~~~~~~~~~~ ~~~~~~~~~~ ~~~~~~~~~~ ~~~~~~~~~~

**14/I/F**  ~~~~~~~~~~ ~~~~~~~~~~ ~~~~~~~~~~ ~~~~~~~~~~ ~~~~~~~~~~

....|....| ....|....| ....|....| ....|....| ....|....|

160 170 180 190 200

**14/I/A**  .......... .......... .......... .......... ..........

**14/I/B**  .......... .......... .......... .......... ..........

**14/I/C**  .......... .......... .......... .......... ..........

**14/I/D**  TAAATGAACT TAGATACCAT TCCGCCCAAC .......... ..........

**14/I/E**  ~~~~~~~~~~ TAGATACCAT TCCGCCCAAC AGCCGGAAGA TATTGCGGTG

**14/I/F**  ~~~~~~~~~~ ~~~~~~~~~~ ~~~~~~~~~~ ~~~~~~~~~~ ~~~~~~~~~~

....|....| ....|....| ....|....| ....|....| ....|....|

210 220 230 240 250

**14/I/A**  .......... .......... .......... .......... ..........

**14/I/B**  .......... .......... .......... .......... ..........

**14/I/C**  .......... .......... .......... .......... ..........

**14/I/D**  .......... .......... .......... .......... ..........

**14/I/E**  CGTCCGGAAC TTGCAAAGAC .......... .......... ..........

**14/I/F**  CGTCCGGAAC TTGCAAAGAC GCAAACGATA TACTACGATC GGAGAAACGG

....|....|

260

**14/I/A**  ..........

**14/I/B**  ..........

**14/I/C**  ..........

**14/I/D**  ..........

**14/I/E**  ..........

**14/I/F**  AGCGTTGAAT 240260 common to clone 14/II

**Clone 14/IV subcloned.**

....|....| ....|....| ....|....| ....|....| ....|....|

10 20 30 40 50

**14/IV/A**  ~~~~~~GTGT AACCAGTTGG ATACCGTCTG CAAGCGAGAA AAATGTGGGG

**14/IV/B**  ~~~~~~~~~~ ~~~~~~~~~~ ~~~~~~~~~~ ~~~~~~~~~~ ~~~~~~GGGG

**14/IV/C**  ~~~~~~~~~~ ~~~~~~~~~~ ~~~~~~~~~~ ~~~~~~~~~~ ~~~~~~~~~~

**14/IV/D**  ~~~~~~~~~~ ~~~~~~~~~~ ~~~~~~~~~~ ~~~~~~~~~~ ~~~~~~~~~~

**14/IV/E**  ~~~~~~~~~~ ~~~~~~~~~~ ~~~~~~~~~~ ~~~~~~~~~~ ~~~~~~~~~~

**14/IV/F**  ~~~~~~~~~~ ~~~~~~~~~~ ~~~~~~~~~~ ~~~~~~~~~~ ~~~~~~~~~~

....|....| ....|....| ....|....| ....|....| ....|....|

60 70 80 90 100

**14/IV/A**  TTAAAGAATC TTTGTT.... .......... .......... ..........

**14/IV/B**  TTAAAGAATC TTTGTTCTTT TAAGCGTATT ACTGATGCAG GGTTTACCTA

**14/IV/C**  ~~~~~~~~~~ ~~~~~~~~~~ ~~~~~~~~~~ ~~~~~~GCAG GGTTTACCTA

**14/IV/D**  ~~~~~~~~~~ ~~~~~~~~~~ ~~~~~~~~~~ ~~~~~~~~~~ ~~~~~~~~~~

**14/IV/E**  ~~~~~~~~~~ ~~~~~~~~~~ ~~~~~~~~~~ ~~~~~~~~~~ ~~~~~~~~~~

**14/IV/F**  ~~~~~~~~~~ ~~~~~~~~~~ ~~~~~~~~~~ ~~~~~~~~~~ ~~~~~~~~~~

....|....| ....|....| ....|....| ....|....| ....|....|

110 120 130 140 150

**14/IV/A**  .......... .......... .......... .......... ..........

**14/IV/B**  TGCTAG.... .......... .......... .......... ..........

**14/IV/C**  TGCTAGAAAC TACGAAGATG ATGGAAGTTG GTATATTAAT CCTGAA....

**14/IV/D**  ~~~~~~~~~~ ~~~~~~~~~~ ~~~~~~GTTG GTATATTAAT CCTGAACTTT

**14/IV/E**  ~~~~~~~~~~ ~~~~~~~~~~ ~~~~~~~~~~ ~~~~~~~~~~ ~~~~~~~~~~

**14/IV/F**  ~~~~~~~~~~ ~~~~~~~~~~ ~~~~~~~~~~ ~~~~~~~~~~ ~~~~~~~~~~

....|....| ....|....| ....|....| ....|....| ....|....|

160 170 180 190 200

**14/IV/A**  .......... .......... .......... .......... ..........

**14/IV/B**  .......... .......... .......... .......... ..........

**14/IV/C**  .......... .......... .......... .......... ..........

**14/IV/D**  TGCATAGTGA AACCAGATTA GCTAATAAGG ATATGT.... ..........

**14/IV/E**  ~~~~~~~~~~ ~~~~~~ATTA GCTAATAAGG ATATGTTTGG TCTAACTTAT

**14/IV/F**  ~~~~~~~~~~ ~~~~~~~~~~ ~~~~~~~~~~ ~~~~~~~~~~ ~~~~~~~~~~

....|....| ....|....| ....|....| ....|....| ....|....|

210 220 230 240 250

**14/IV/A**  .......... .......... .......... .......... ..........

**14/IV/B**  .......... .......... .......... .......... ..........

**14/IV/C**  .......... .......... .......... .......... ..........

**14/IV/D**  .......... .......... .......... .......... ..........

**14/IV/E**  GACCCGAATA AGCAATATTA TCTATT.... .......... ..........

**14/IV/F**  ~~~~~~AATA AGCAATATTA TCTATTCATA GACGCTGCTA AAAGGACTGA

....|....| ....|....| ..

260 270

**14/IV/A**  .......... .......... ..

**14/IV/B**  .......... .......... ..

**14/IV/C**  .......... .......... ..

**14/IV/D**  .......... .......... ..

**14/IV/E**  .......... .......... ..

**14/IV/F**  TACAGAAACG GACAAA.... ..

**Clone 14/IX subcloned.**

....|....| ....|....| ....|....| ....|....| ....|....|

10 20 30 40 50

**14/IX/A**  ~~~~~~ATAG GCATATATCA AAGCTAATAG ATTTAAGAGG ATGGGACAAT

**14/IX/B**  ~~~~~~~~~~ ~~~~~~~~~~ ~~~~~~~~~~ ~~~~~~~~~~ ~~~~~~CAAT

**14/IX/C**  ~~~~~~~~~~ ~~~~~~~~~~ ~~~~~~~~~~ ~~~~~~~~~~ ~~~~~~~~~~

**14/IX/D**  ~~~~~~~~~~ ~~~~~~~~~~ ~~~~~~~~~~ ~~~~~~~~~~ ~~~~~~~~~~

**14/IX/E**  ~~~~~~~~~~ ~~~~~~~~~~ ~~~~~~~~~~ ~~~~~~~~~~ ~~~~~~~~~~

**14/IX/F**  ~~~~~~~~~~ ~~~~~~~~~~ ~~~~~~~~~~ ~~~~~~~~~~ ~~~~~~~~~~

....|....| ....|....| ....|....| ....|....| ....|....|

60 70 80 90 100

**14/IX/A**  AATAAGTTTT TCCCGT.... .......... .......... ..........

**14/IX/B**  AATAAGTTTT TCCCGTTGGT TATAAATATT CCGGTTTACC ACAAAACAAG

**14/IX/C**  ~~~~~~~~~~ ~~~~~~~~~~ ~~~~~~~~~~ ~~~~~~TACC ACAAAACAAG

**14/IX/D**  ~~~~~~~~~~ ~~~~~~~~~~ ~~~~~~~~~~ ~~~~~~~~~~ ~~~~~~~~~~

**14/IX/E**  ~~~~~~~~~~ ~~~~~~~~~~ ~~~~~~~~~~ ~~~~~~~~~~ ~~~~~~~~~~

**14/IX/F**  ~~~~~~~~~~ ~~~~~~~~~~ ~~~~~~~~~~ ~~~~~~~~~~ ~~~~~~~~~~

....|....| ....|....| ....|....| ....|....| ....|....|

110 120 130 140 150

**14/IX/A**  .......... .......... .......... .......... ..........

**14/IX/B**  GGTTGA.... .......... .......... .......... ..........

**14/IX/C**  GGTTGAAATA AGTAGACCTC TTGATGCGGG ATACGGAAAA CCTTCA....

**14/IX/D**  ~~~~~~~~~~ ~~~~~~~~~~ ~~~~~~CGGG ATACGGAAAA CCTTCATACG

**14/IX/E**  ~~~~~~~~~~ ~~~~~~~~~~ ~~~~~~~~~~ ~~~~~~~~~~ ~~~~~~~~~~

**14/IX/F**  ~~~~~~~~~~ ~~~~~~~~~~ ~~~~~~~~~~ ~~~~~~~~~~ ~~~~~~~~~~

....|....| ....|....| ....|....| ....|....| ....|....|

160 170 180 190 200

**14/IX/A**  .......... .......... .......... .......... ..........

**14/IX/B**  .......... .......... .......... .......... ..........

**14/IX/C**  .......... .......... .......... .......... ..........

**14/IX/D**  GTACACATGA TGGCGGTTTT TCTATGAACT TAACGT.... ..........

**14/IX/E**  ~~~~~~~~~~ ~~~~~~TTTT TCTATGAACT TAACGTTTGA GATGTCCGGT

**14/IX/F**  ~~~~~~~~~~ ~~~~~~~~~~ ~~~~~~~~~~ ~~~~~~~~~~ ~~~~~~~~~~

....|....| ....|....| ....|....| ....|....| ....|....|

210 220 230 240 250

**14/IX/A**  .......... .......... .......... .......... ..........

**14/IX/B**  .......... .......... .......... .......... ..........

**14/IX/C**  .......... .......... .......... .......... ..........

**14/IX/D**  .......... .......... .......... .......... ..........

**14/IX/E**  TCGGGTTGGG GTTCGTTGCC AGCAGT.... .......... ..........

**14/IX/F**  ~~~~~~TGGG GTTCGTTGCC AGCAGTAACT AATATCTTTG ACTATACTAA

....|....| ....|....| ..

260 270

**14/IX/A**  .......... .......... ..

**14/IX/B**  .......... .......... ..

**14/IX/C**  .......... .......... ..

**14/IX/D**  .......... .......... ..

**14/IX/E**  .......... .......... ..

**14/IX/F**  AGCATGGACT TCTGCG.... ..

**d. Phage lambda fragments ligated into pUC19.** These constructs are all members of the "N" subclass of the pY collection (held at New England Biolabs by Rich Roberts and Iain Murray) which means that they were generated by random cleavage of lambda DNA using a nebulizer, followed by end repair and ligation to SmaI cut and dephosphorylated pUC19 DNA. The insert sequences were checked by DNA sequencing.

**name /** start base / end base / length in bases**)**

**pY33** 3213 4112 899

**pY225** 12375 13278 903

**pY667** 43490 44394 904

**pY627** 40457 41363 906

**pY493** 29816 30727 911

**pY524** 31742 32664 922

**pY124** 8239 9169 930

**pY696** 44863 45807 944

**pY4** 650 1595 945

**pY107** 7366 8336 970

**pY361** 18580 19585 1005

**pY401** 20486 21497 1011

**pY432** 21807 22827 1020

**pY378** 19563 20602 1039

**pY614** 39653 40704 1051

**pY698** 44986 46049 1063

**e. Determination of candidate sites using synthetic duplexes ligated into the EcoRI-BamHI interval of pUC19.**

Candidate site for CC5-1: AGGNNNNNGAT

5’-AATTCTGAGGCATATGATGAA-3’

3’-GACTCCGTATACTACTTTCGA-5’

Candidate CC5-1 site highlighted in yellow.

Candidate site for CC5-2: TACNNNNNNRTG

5’- AATTCTGTACCATATGATGGAA-3’

3’-GACATGGTATACTACCTTTCGA -5’

Candidate CC5-2 site highlighted in yellow.

Candidate site for CC1-1: TTAANNNNNRTGG

5’-AATTCTGTAAGTTAAGGACAGTGGGAA-3’

3’-GACATTCAATTCCTGTCACCCTTTCGA-5’

Candidate CC1-1 site highlighted in yellow.
